# A multimodal intervention of manual therapy, exercise, and psychological management for painful diabetic neuropathy: intervention development and feasibility trial protocol

**DOI:** 10.1080/17581869.2025.2515010

**Published:** 2025-06-11

**Authors:** David Hohenschurz-Schmidt, Sasha Smith, Annina B Schmid, Philip Bright, Jerry Draper-Rodi, Matthew C Evans, Harriet Kemp, Nuno Koch Esteves, Elizabeth Pigott, Whitney Scott, Jan Vollert, Esther Williamson, Esther M Pogatzki-Zahn, Steven Vogel

**Affiliations:** a Imperial Clinical Trials Unit, School of Public Health, Imperial College London, London, UK; b Section of Vascular Surgery, Department of Surgery and Cancer, Imperial College London, London, UK; c Imperial Vascular Unit, Imperial College Healthcare NHS Trust, Charing Cross Hospital, London, UK; d Nuffield Department of Clinical Neurosciences, Oxford University, John Radcliffe Hospital, Oxford, UK; e UCO School of Osteopathy, Health Sciences University, London, UK; f National Council for Osteopathic Research, c/o Health Sciences University, London, UK; g Division of Anaesthetics Pain Medicine & Intensive Care, Imperial College London, London, UK; h THERMOSENSELAB, Skin Sensing Research Group, School of Health Sciences, The University of Southampton, Southampton, UK; i Division of Sport, Health and Exercise Sciences, Department of Life Sciences, Brunel University of London, Uxbridge, UK; j Patient partner, London, UK; k King’s College London, Health Psychology Section, Institute of Psychiatry, Psychology, and Neuroscience, London, UK; l INPUT Pain Management Unit, Guy’s and St Thomas’ NHS Foundation Trust, London, UK; m Department of Clinical and Biomedical Sciences, Faculty of Health and Life Sciences, University of Exeter, Exeter, UK; n Nuffield Department of Orthopaedics, Rheumatology and Musculoskeletal Sciences, University of Oxford, Oxford, UK; o Public Health and Sports Science, University of Exeter, Exeter, UK; p Department of Anaesthesiology, Intensive Care and Pain Medicine, University Hospital Muenster, Muenster, Germany

**Keywords:** Neuropathic pain, physical therapy modalities, rehabilitation, mind-body therapy, osteopathic manipulative treatment, acceptance and commitment therapy, neurodynamic treatment, Placebo

## Abstract

**Background:**

Therapeutic options for people experiencing neuropathic pain from diabetic peripheral neuropathy are limited, and impact can be severe. Physical and psychological interventions remain under-explored but may offer promise, especially in multimodal combination programs.

**Objectives and methods:**

To address this gap, an intervention was developed according to the Medical Research Council’s framework for complex interventions, including research expert and stakeholder input. This will be tested for acceptability and feasibility in a trial.

**Results:**

NeuOst (Neuropathy Optimisation through Self-management and Therapy) is a manual therapy-based intervention, incorporating exercise, psychologically informed training, and education. The protocol for a single-site, parallel, three-arm, partially participant-blinded, randomized controlled trial is presented. The experimental treatment is a 5-week course of NeuOst as adjunct to patients’ usual care. Comparators are a control intervention that lacks pre-specified components of interest as adjunct to usual care and usual care only in adults with painful diabetic peripheral neuropathy. The follow-up period is 16 weeks. Primary outcomes are feasibility measures such as recruitment, eligibility, and consent rates, retention, blinding, fidelity, acceptability, and safety. Secondary and exploratory outcomes involve clinical measures and qualitative feedback. A protocol was prospectively registered (NCT06423391).

**Conclusion:**

After initial intervention development, a feasibility trial will inform intervention refinement and future research steps.

**Clinical trial registration:**

www.clinicaltrials.gov identifier is NCT06423391.

## Plain Language Summary

People with diabetes can develop nerve pain in their feet, which can greatly affect quality of life. Current treatments are limited. Physical and psychological approaches – like exercise, massage, and supportive conversations – may help but are rarely combined or fully tested.

We have developed a new treatment program called NeuOst, which brings together hands-on therapy, exercise, and conversations for pain management. It was designed with people with lived experience, healthcare professionals, and researchers. We are now running a small study to test whether this program is practical to deliver and acceptable to participants.

Adults with painful diabetic neuropathy are randomly placed into one of three groups (in addition to their normal care outside the study):

- NeuOst (5 sessions)
- A comparison treatment (similar to NeuOst but with some elements removed; 5 sessions)
- Usual care only

We follow participants for 16 weeks and look at how the program was delivered, how people experienced it, and any early signs of benefit.

The results will guide improvements to the treatment and inform future research.

## 1. Introduction

Diabetes mellitus is a leading and increasing global health concern [1]. Diabetic peripheral neuropathy (DPN), a common complication, affects 20–40% of patients and often goes undiagnosed, potentially leading to severe foot complications [1,2]. About 26% of those with DPN experience painful diabetic peripheral neuropathy (pDPN), which reduces quality of life [3] and increases the risk of anxiety and depression [4,5]. Musculoskeletal pain is also up to twice as prevalent in people with diabetes [6], linked to higher BMI and sedentary lifestyles [7]. Both diabetes and musculoskeletal pain are disproportionately prevalent in socioeconomically marginalized communities [8,9].

Clinical management guidelines for diabetes and neuropathy recommend combining pharmacotherapy with nonpharmacological options such as lifestyle modifications and cardiovascular risk interventions [10,11]. Experts also emphasize addressing sleep, functionality, and quality of life alongside pain management [11]. In practice, however, pDPN treatment often relies on pharmacological interventions [12,13], despite their limited efficacy and various side effects [14–16]. Calls for more person-centered pain management exist, including physical and psychological approaches [17–19].

Research into nonpharmacological treatments for pDPN is limited. Systematic reviews highlight a lack of high-quality studies, particularly for manual therapy and exercise-based interventions, despite preliminary evidence of efficacy [20,21]. Reviews also point to an absence of trials for psychological interventions, despite the link between neuropathic pain and psychological distress [4]. Authors call for more long-term and high-quality studies, and the exploration of additional nonpharmacological treatments for painful peripheral neuropathies [22–24].

Allied Health Professionals, such as osteopaths or physiotherapists, may be well-placed to address this gap: They are trained in pain management, lifestyle advice, and differential diagnosis [25–27]. Also, combinational or adjunct psychological approaches are increasingly explored for pain management, including psychologically informed physiotherapy [28], and ‘augmentations’ of osteopathic manual therapy with cognitive-behavioral techniques [29]. However, the application of these approaches to pDPN remains underexplored.

This article outlines the development of ‘NeuOst’ (Neuropathy Optimisation through Self-management and Therapy), a multimodal manual-therapy-based and psychologically informed intervention for people living with pDPN, and details the protocol of a feasibility randomized controlled trial (RCT) to inform further research and intervention refinement.

## 2. Methods

**Figure 1.**
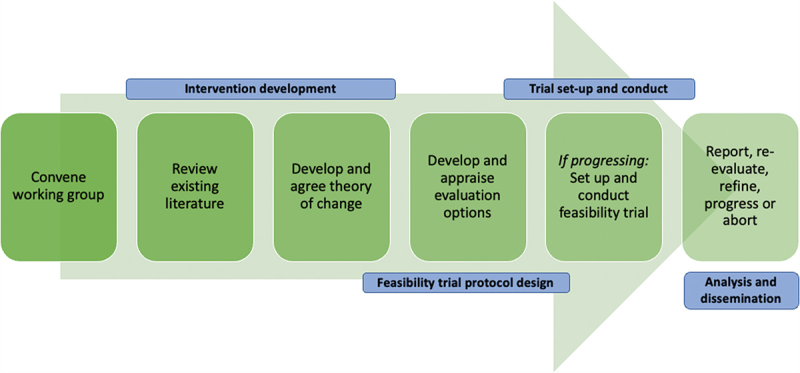
Process flowchart of intervention development and feasibility evaluation. *Adapted from: Craig P, Campbell M. Evaluability assessment: a systematic approach to deciding whether and how to evaluate programmes and policies: a what works Scotland working paper. 2015*. adapted from [30].

The process of intervention development and feasibility testing was structured as illustrated in Figure 1 [30].

### 2.1. Intervention development

Development of the NeuOst intervention drew on the Medical Research Council (MRC) framework for iterative complex interventions development and testing, focusing on the core elements of considering intervention context, identifying and engaging stakeholders, developing and testing program theory, and identifying key uncertainties [31]. The main methods for identifying potential intervention components and defining a program theory were collaboration within an expert working group, stakeholder engagement, and using available systematic literature reviews.

The working group of clinical research experts involved in intervention development included four osteopaths (DHS, SV, JDR, PB), three anaesthetists and pain physicians (HK, EPZ, ASCR, see acknowledgments), a diabetes specialist nurse (KW, see acknowledgments), a clinical psychologist (WS), a neurologist (ME), and two physiotherapists (AS, EW). Most were also pain researchers (DHS, AS, HK, EPZ, ASCR, ME, WS). The group was led by DHS who hosted group meetings, shared collaboration documents, visited individual group members for specific tasks, and ensured that insights from stakeholder engagement informed all discussions.

Based on reviews [20,21,32–35] and key studies found therein, the working group developed a table of potential intervention components, defining each component’s theoretical therapeutic mechanism, its potential role within NeuOst and interactions with other components, and the available evidence of efficacy/effectiveness. During intervention development, this table informed discussions among the research team and during stakeholder engagement.

Stakeholder engagement was facilitated by purposively sampling individuals living with neuropathic pain – recruited via social media and a local flyer campaign – as well as osteopathic or physiotherapy clinicians experienced in the care of such individuals. Stakeholders then discussed with the chief investigator (DHS), using a list of guiding questions that was iteratively refined as intervention development progressed, focusing on their lived experience with neuropathy, care experiences, self-management strategies, and attitudes toward potential NeuOst intervention components. Transcripts of these discussions were analyzed by DHS using thematic coding and categorization methods aligned with reflexive thematic analysis [36]. Individuals with lived experience were also invited to in-person testing of intervention components. Five patients with polyneuropathy participated in online conversations, with one individual interviewed twice. One of these patients also attended an in-person session to test the combined intervention components, joined by a sixth person living with pDPN. All but one participant had Type 2 diabetes with varying degrees of neuropathy, while one had chemotherapy-induced neuropathy. The average age was 74, with three women and three men. In terms of ethnicity, four participants were white, one was Black-Caribbean, and one was Asian. Additionally, three osteopaths and one physiotherapist were interviewed about experiences with neuropathies and chronic pain management. Two osteopaths had worked as tutors in a teaching clinic for people living with HIV (a patient population where peripheral neuropathy is common [37]), while the third had worked over a sustained period treating an individual with chemotherapy-induced neuropathy. The physiotherapist led an outpatient pain management program in Germany. All providers had over 20 years of experience. Recruitment of physical or manual therapists with extensive pDPN experience was unsuccessful.

Methods for a feasibility and acceptability evaluation are detailed in the below trial protocol. Further intervention refinement may occur following the feasibility study, before potentially moving into an evaluation of clinical effects, healthcare economics, and implementation research.

### 2.2. Protocol development

Development of the feasibility trial protocol was done in a similarly iterative and collaborative manner, with more input from experts in trial methodology and experienced trialists (EW, SS, AS, JV, EPZ, WS, NKE, ASCR) and from a patient partner (EP). This included theoretical and practical development of the control intervention, as detailed in Supplementary Material 1.

## 3. Results

### 3.1. The NeuOst intervention

The NeuOst intervention, as developed so far and evaluated in the feasibility trial, is delivered by UK osteopaths in individual treatment sessions. The intervention incorporates case history-taking, neurological and manual therapy examination, complication screening, disease education and lifestyle advice, manual therapy applications and neurodynamic exercises, a structured exercise program performed at home, and experiential exploration of pain management strategies informed by Acceptance and Commitment Therapy (ACT) [38].

In the feasibility trial, fully qualified osteopaths deliver the treatment in five 60-min 1:1 sessions (first session 90 min), using a treatment manual that specifies obligatory and optional elements (Supplementary Material 2). In each session, therapists employ manual therapy techniques, and conduct ACT-informed discussions with patients, structured through dedicated worksheets. During sessions, patients practise physical exercises and are encouraged to follow a progressive home-exercise program. A ‘wobble board’ is provided to facilitate balance exercises at home.

#### 3.1.1. Intervention context and rationale

The NeuOst intervention was designed to address neuropathic pain from DPN within the UK’s socio-political and population health context of rising diabetes prevalence, healthcare disparities, and a lack of specialist services and effective treatments for those living with painful DPN in the UK National Health Service (NHS) [16,39,40]. In this context, NeuOst aims to reduce the personal and societal burden from pDPN [4]. NeuOst targets gaps in nonpharmacological pain management by upskilling qualified osteopaths, typically focused on musculoskeletal care [26], to provide biopsychosocial care for people living with pDPN. NeuOst aims to promote patient wellbeing while addressing structural barriers such as low income and lack of services [40]. In the feasibility trial, this meant that the treatment was delivered for free in a charitable clinic affiliated with a teaching institution, and we aim to explore incorporation into the NHS in future steps. More generally, the intervention also aspires to influence the osteopathic community’s and other Allied Health professions’ roles in an integrative healthcare framework.

#### 3.1.2. Stakeholder engagement

Upon agreeing an initial theoretical framework among the academic working group, NeuOst development involved people living with neuropathic pain from polyneuropathies and osteopaths or physiotherapists experienced in their treatment. Patient and provider engagement highlighted the topics in Table 1.

**Table 1.** Key topics from stakeholder involvement discussions with people living with peripheral polyneuropathy and clinicians experienced in their care, as part of the stakeholder engagement during the NeuOst intervention development phase. Where appropriate, key insights are contextualized with references to external research studies.

Key insight	How this shaped NeuOst development
Diagnostic uncertainty and education gaps	Several patients had not been formally diagnosed with neuropathy or expressed the desire to know more about the condition (also see [41,42]). This informed the incorporation of neurological testing and educational elements into NeuOst to increase patient understanding of their condition and treatment options.
Patient interest in nonpharmacological options	Many patients expressed frustration with medications, particularly due to side effects. They highlighted the need for a nonpharmacological treatment approach (also see [18,19,43]), such as manual therapy, which encouraged us in our approach. At the same time, we are aware of a potential sampling bias and aim to explore care attitudes in a broader population during separate projects. Acknowledging that exercise adherence can be challenging and that patients have different preferences and needs – including a need to feel safe while exercising – we designed NeuOst to incorporate flexible exercise components that accommodate varying levels of patient engagement and confidence.
Impact of symptoms on quality of life	Sleep disturbances, balance problems, fear of falling, and avoidance of foot ulcerations were prominent issues (also see [3,4,42]), leading to the inclusion of therapeutic components aimed at reducing or better managing these symptoms, including manual therapy, psychological approaches, sensorimotor exercises, and foot care education.
Clinician perceptions of manual therapy	Clinicians saw manual therapy as providing short-term relief but not life-changing benefits, and believed that no elaborate theoretical models were required to support manual therapy applications. They related patient stories where even a few hours of relief were deemed worthwhile by patients, but highlighted that this meant regular (e.g., weekly) treatment that was unlikely to be possible in private practice settings or the NHS. This feedback guided the design of NeuOst to incorporate repeated treatment sessions, prolonged mobilization techniques for the lower extremities, and to aim for realistic outcomes, addressing mechanisms beyond those directly relevant for symptomatic relief (such as cognitive-emotional changes, improved knowledge, and lifestyle modifications). We aim to explore incorporation into public health services during future development stages.
Focus on broader care beyond physical treatment	Clinicians emphasized the importance of addressing behavior change and false pain beliefs, which led to the incorporation of elements that promote self-management. They also recognized the need for person-centered support in managing multiple long-term conditions, a perspective that was reinforced by patient narratives highlighting the interconnection between neuropathy and diabetes (also see [43]). This informed the development of NeuOst provider training, aiming to equip clinicians with an understanding of relevant pathophysiology, common clinical management, and the ability to screen for and manage (where appropriate) complications such as ulcers and hypoglycemic episodes. The training also included guidance on signposting patients within the wider healthcare system.

#### 3.1.3. Program theory

With the expert working group and informed by stakeholders, we developed the NeuOst intervention, a multimodal program combining manual therapy with cognitive-behavioral, exercise, and educational components to treat pDPN. The program theory recognizes interplays between neurological, musculoskeletal, cardiovascular, and psychological factors in pDPN and its quality-of-life impacts, aiming to target these mechanisms through various components.

**Figure 2.**
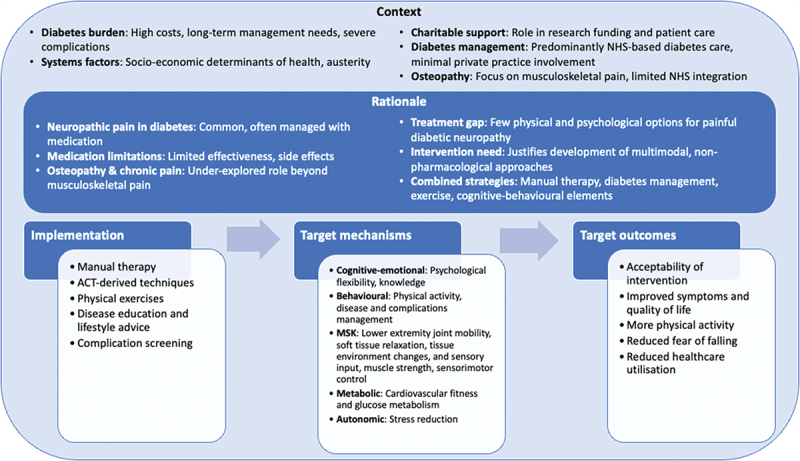
NeuOst logic model, describing the intervention context, rationale, key resources, intervention components, target outcomes and mechanisms, and hypothesized wider impacts. ACT, acceptance and commitment therapy; MSK, musculoskeletal. Created based on available guidance for the development of logic models for complex interventions: https://www.Gov.uk/guidance/evaluation-in-health-and-wellbeing-creating-a-logic-model & https://www.strategyunitwm.nhs.uk/sites/default/files/2018-03/Logic%20models%20%20complex%20programmes%20-%20a%20brief%20guide.pdf [41].

The manual therapy components aim to improve joint mobility, reduce pain, and provide sensory input, while the exercise components focus on enhancing strength, balance, and cardiovascular fitness. Psychological components, informed by ACT, aim to promote psychological flexibility to improve daily functioning and reduce distress in the presence of pain. Educational elements should improve patients’ understanding of diabetes, pain management strategies, and healthy lifestyle choices. Furthermore, NeuOst emphasizes treatment personalization through flexibility in several intervention components, and compatibility with standard medical care. Figure 2 presents the intervention’s logic model, with detailed components and mechanisms in Supplementary Material 2.

The agreed program theory informed a treatment manual for the feasibility trial (Supplementary Material 2) and a provider training course for osteopaths.

#### 3.1.4. Key uncertainties and research perspectives

Key uncertainties after initial intervention development included the intervention’s deliverability and acceptability and the feasibility of conducting future RCTs to assess efficacy, effectiveness, and healthcare economics.

### 3.2. Feasibility trial protocol

Apart from testing acceptability and feasibility, a feasibility RCT is needed to facilitate refinement of treatment components and trial design if necessary [31,42].

This RCT protocol is reported as per 2013 SPIRIT Statement (Defining Standard Protocol Items for Clinical Trials) [43] (Supplementary Material 3) – based on trial protocol version 1.0, dated 22 March 2023 - and interventions according to other relevant reporting guidelines [44,45] (Supplementary Material 4). The trial was registered on ClinicalTrials.gov on 15 May 2024 (NCT06423391) and with a full protocol on the Open Science Framework on 29 May 2024 (https://osf.io/jyftb).

The trial was funded by *The Osteopathic Foundation* and *The Alan and Sheila Diamond Charitable Trust*. Sponsor institution is the Health Sciences University (HSU) – UCO School of Osteopathy.

#### 3.2.1. Research ethics approval

The study was approved by the sponsor’s institutional research ethics committee on 5 April 2024.

#### 3.2.2. Study objectives

This study’s primary objective is to test the feasibility of a single-site, three-parallel-arm, partially participant-blinded RCT of a 5-week course of NeuOst (as adjunct to usual care), compared to a designated control intervention (plus usual care) that lacks pre-specified components of interest to enable their study, and compared to usual care alone in adults with pDPN. Secondary objectives are to obtain indications of the potential efficacy of NeuOst, including estimates of between-group treatment effects and their variance on key pain/disability outcomes. Further, qualitative data about recruitment routes, reasons for exclusion during screening, and participant and provider experiences during the feasibility trial will be collected where possible.

The development and nature of the control intervention, as well as detailed feasibility criteria are described below.

#### 3.2.3. Trial design

**Figure 3.**
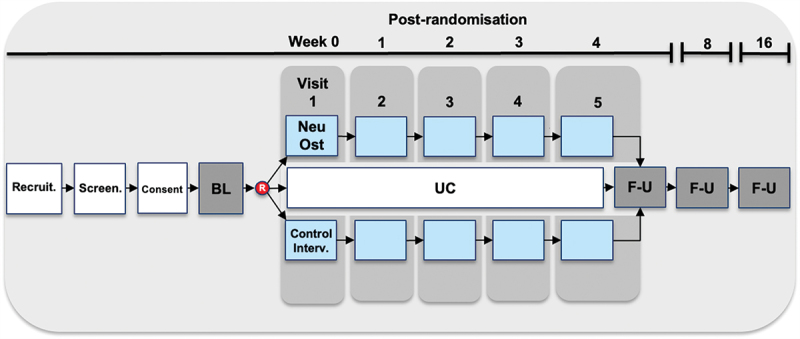
Flow diagram of the NeuOst feasibility trial. Interventions are described in detail in the manuscript text and appendices, with ‘control intervention’ referring to a specifically designed intervention replicating the tested (‘NeuOst’) intervention but omitting components the effects of which are to be studied in the trial. All three trial groups continue their usual care outside the trial. BL, baseline assessment; F-U, follow-up assessment; R, randomization; UC, usual care.

This is a prospective, randomized, parallel-arm, three-group, controlled, partially blinded, clinical feasibility trial, employing a 16-week follow-up period (Figure 3).

#### 3.2.4. Study setting

The NeuOst feasibility trial is conducted at the HSU – UCO School of Osteopathy clinic in central London (UK), an accessible facility serving a diverse local population. Trial-related appointments and clinical notes are integrated into an existing management system.

#### 3.2.5. Study participants

##### 3.2.5.1. Eligibility criteria and screening

Eligible are adults with pDPN, irrespective of most comorbidities and medication use. Eligibility is evaluated during a screening phone call (Figure 3), encompassing a structured interview covering all eligibility criteria (Table 2).

**Table 2.** Eligibility criteria for participants in the NeuOst feasibility trial [46,47].

Inclusion criteria	Exclusion criteria
Medical diagnosis of diabetes mellitus (type 1 or type 2) as per patient self-report. Distal symmetrical peripheral neuropathy (defined as a score of ≥4 on the Michigan Neuropathy Screening Instrument (questionnaire part) [47]). Neuropathic pain in the legs or feet as assessed by DN4 interview questionnaire (defined as a score of ≥3 of 7 self-reported items) [47]. Stable analgesic medication regimen for at least 3 weeks prior to anticipated study enrolment and no revision of regimen planned within the next 3 months. Stable attendance of out-of-study nonpharmacological therapies for neuropathic pain or professional exercise programs (including gym classes and manual therapy) for at least 3 weeks prior to anticipated study enrolment and no revision of routine planned within the next 3 months. Participants able and willing to provide informed consent. Participants able to speak, understand, and read English at conversational levels. Age 18 and older.	Contraindications to manual therapy and conservative pain management (as determined by recruiting staff during screening calls or the trial provider at the initial or any other appointment; May include medical emergencies and suspected severe pathology, advanced osteoporosis, and active foot ulcerations). Past or scheduled amputations. Advanced renal failure. * Recent physical trauma with suspected or unclear fracture. Currently experiencing severe depression or other current and severe/poorly controlled psychopathology such as bipolar/psychosis, and/or presenting with active suicidal risk. Carpal Tunnel Syndrome diagnosis or symptoms as the only source of neuropathic pain. Unable to attend in-person appointments at treatment site (for health/organizational reasons). Knowing other participants signed up to the study (to avoid group contamination). Concomitant participation in another clinical intervention study.

*In a protocol amendment, this exclusion criterion was further specified as not requiring hospital-based renal dialysis, irrespective of the diagnostic stage of renal failure.

##### 3.2.5.2. Recruitment

Participants are recruited through online and print advertising, including social media, newsletters, posters and flyers (Supplementary Material 5) in community spaces and private clinics (mainly osteopathy and podiatry), and the study website. Initially excluding NHS services, recruitment was later expanded to include self-referrals from local NHS diabetes clinics, with staff informing eligible patients about the study during routine appointments without accessing patient records.

#### 3.2.6. Consent

The consent process consists of verbal consent for telephone screening and full written consent for trial participation before the baseline assessment. Upon review of the Participant Information Sheet (PIS, Supplementary Material 6), signed consent is collected via post, email, or in person (Supplementary Material 7). Capacity to consent is assessed through discussion and, if necessary, a validated questionnaire [48].

#### 3.2.7. Sample size and power

We aim to recruit 12 patients per group. This sample size was chosen pragmatically.

For context, a hypothetical two-armed trial would require 100 participants per arm to detect a 1.2-point mean difference in pain intensity (SD 3.0) at 80% power and α = 0.05, and 35 participants per arm to detect the typically cited clinically meaningful 2-point reduction in pain scores (SD 3.0) [49].

However, determining efficacy is not an objective of this feasibility study. Instead, this trial will help refine the sample size estimate for future RCTs, and may also suggest futility [50,51].

#### 3.2.8. Assignment of interventions

Blocked randomization is used with permuted block sizes of 3–6 participants and a 1:1:1 allocation ratio, without stratification or minimization. Participants are randomized upon completion of baseline assessments. The randomization sequence is generated and provided by the web-based system *Sealed Envelope*® (Sealed Envelope Ltd, London, UK), preventing researcher influence over group allocation. To maintain blinding, coded labels are used to inform treatment providers of assignments, the meaning of which is known only to providers.

#### 3.2.9. Interventions

Participants in all groups continue their usual care outside the trial, in addition to trial-related processes.

##### 3.2.9.1. Tested intervention (NeuOst treatment)

The NeuOst treatment is described above, in the appended intervention description following the TIDieR reporting checklist [44], and in the appended treatment manual as used during this trial (Supplementary Material 2). In the trial, patients are encouraged to attend one of the five treatment sessions each week, and a session is considered ‘missed’ if intervals between two sessions exceed 2 weeks.

##### 3.2.9.2. Control intervention

The control intervention was designed to replicate the NeuOst intervention while omitting components of interest for scientific study. While sometimes called ‘placebo’ or ‘sham,’ trial communication and this protocol avoid these terms, in line with current guidance [45].

The control intervention’s objectives are 1) to isolate the effects of intervention components of interest, 2) to blind participants in the test and control intervention arms to their group allocation, and 3) to balance expectancy effects between those two study arms. It was developed upon completion of the NeuOst intervention’s development, following current best-practice guidance for control interventions in mechanistic and efficacy trials [45]. This included conceptual discussions within the working group, and two practical piloting sessions with designated trial providers and the chief investigator and two people with lived experience.

In the control intervention arm, the same trial providers deliver a modified version of NeuOst, using a manual and having regular supervision with the chief investigator. Key differences to the NeuOst test intervention include reduced (minimal) force and amplitude in manual techniques, omission of specific physical exercises, and alterations to psychologically informed strategies (e.g., replacing ACT-based exercises with generic discussions of patients’ pain experiences). Physical exercise advice in the control arm is generalized and minimally supervised. Matched elements include session length, frequency and amount, therapeutic relationship, advice on foot care, provision of a ‘wobble board,’ and the clinical environment. The control intervention is described in more detail, and the trial manual is provided in Supplementary Material 1.

##### 3.2.9.3. Usual care arm

Participants allocated to the usual care (UC) comparator, continue any existing treatments for pDPN or other medical conditions. They do not receive any attention or information as part of the trial, except for attending assessment calls as described below.

#### 3.2.10. Concomitant care

Participants across all trial arms are encouraged to maintain stable medication regimens and out-of-trial activities related to DPN care throughout the study. However, no formal restrictions are applied beyond the eligibility criteria. Usual medical care continues in all trial arms. Data on out-of-trial healthcare use, including healthcare providers, self-management practices, physical activity, and medication use, are collected via patient-report at baseline and follow-ups.

#### 3.2.11. Trial provider training and eligibility

Trial providers are fully qualified, General Osteopathic Council (GOsC)-registered osteopaths trained specifically to deliver NeuOst and the control intervention. Training comprised a 7-h online learning program on diabetes, neuropathic pain, exercise, manual therapy, and trial protocols, followed by an 8 h in-person training with practical modules (e.g., ACT for pDPN, neurological exams, patient-centered communication, manual therapy, and exercise protocols). Additional sessions with the chief investigator focused on refining trial processes and control intervention application, hands-on practice with patient partners, and scripting intervention processes. Providers were encouraged to review materials and practice NeuOst techniques with patients or peers. During the trial, all trial providers attend supervision sessions with the trial’s clinical psychologist who has 10+ years’ experience delivering ACT for persistent pain (WS), occurring every 2–3 months to discuss the ACT-related intervention and control procedures. Supervision sessions take a problem-oriented and questions-and-answer approach, with both the clinicians and the supervisor proposing discussion topics.

Providers are recruited via social media, a professional magazine, and HSU staff outreach. Eligibility required active GOsC registration, at least 1 year of clinical experience, completion of NeuOst training, attendance at trial-specific sessions, and indemnity insurance. Exclusions applied to those with extensive experience treating polyneuropathies, specialized diabetes training, or teaching roles in the HIV-related HSU clinic.

Of 23 trainees, 11 expressed interest and 9 became trial providers. Competency was verified through a quiz, review of engagement with training resources, and chief investigator observation during the training.

#### 3.2.12. Blinding

Blinding is attempted for trial participants (except in the usual care group), administrative staff, and the research team, including outcome assessors and statistician. The control intervention, which mimics the NeuOst intervention, aims to blind to allocation to NeuOst or control intervention. Providers and staff are trained to maintain blinding, with measures like staggered appointments and excluding participants who know others, to minimize contamination. Unblinding occurs only after data analysis or in emergencies with trial oversight committee approval.

#### 3.2.13. Outcome measures

Outcome data are collected at baseline, at the end of the treatment period (approx. 5 weeks), and 3 and 11 weeks thereafter (approx. 8 and 16 weeks after randomization, depending on clinic appointment scheduling) (Figure 3). Data are also collected from participants in the intervention and control intervention groups (but not the Usual Care group) prior to each clinical appointment.

##### 3.2.13.1. Primary (feasibility) outcomes

Feasibility of a full-scale trial will be assessed using pre-specified criteria and progression rules across several feasibility outcomes. Recruitment, eligibility, and consent rates will be evaluated based on the number of participants recruited within defined timeframes, the proportion of screened participants found eligible, and the percentage of eligible participants providing consent. Retention rates will be determined by participant attrition at the 16-week follow-up. Blinding effectiveness will be assessed based on allocation guesses at the first follow-up, using Bang’s blinding index [52]. Interventionist fidelity will be measured by the proportion of fidelity assessments confirming protocol adherence. Data completeness will be assessed by the percentage of missing data at the 16-week follow-up. Treatment acceptability will be measured through participant satisfaction ratings, attendance rates of clinical appointments, and qualitative feedback from interviews and feedback forms. Adverse events will be monitored, with minor related side effects (e.g., transient soreness) considered acceptable in up to 80% of patients, but suspected unexpected serious adverse reactions (SUSARs) prompting reassessment. Progression to a full trial will be guided by a traffic-light system, with predefined thresholds for green (proceed), amber (amendments required), and red (stop) (see full protocol on https://osf.io/jyftb).

##### 3.2.13.2. Secondary outcomes

Secondary outcomes assess clinical measures for potential use as primary endpoints in future trials. These are pain in the feet and disability (measured by the Brief Pain Inventory [53–55] and Diabetic Peripheral Neuropathic Pain Impact Measure [56,57]), and quality of life and function (NeuroQoL [58] and Falls Efficacy Scale-International [59–61]).

##### 3.2.13.3. Exploratory outcomes

Exploratory measures contextualize primary and secondary outcomes, including symptom bothersomeness and tolerability [62–64], patient global impression of change [55,65], medication and intervention use, expectancy and credibility measures (assessed at baseline and prior to visit 2) [66,67], and qualitative feedback from participants and providers on perceived benefits, harms, mechanisms, and trial design suggestions. Additionally, participants and providers will be invited to a dedicated qualitative interview study after completion of their trial involvement (separate ethics approval sought). By discussing their perspectives on trial processes and intervention components, this study seeks to identify enablers and barriers to engagement, and inform adaptations, including for diverse patient populations. By incorporating a theory-based so-called ‘realist evaluation,’ this study also seeks to further refine the NeuOst intervention theory by exploring how, why, and for whom the intervention produced certain outcomes [68].

#### 3.2.14. Data collection methods

Data sources include electronic case report forms (CRFs) capturing patient-reported measures during telephone/videocall interviews, provider-completed paper CRFs, and specific forms for recording safety data. The electronic data capture (EDC) system was self-built using Microsoft Forms and Excel. Clinical questionnaires are administered via patient self-report, either in person or during telephone/videocall interviews with a blinded investigator or as paper copies prior to clinical appointments, which are later entered into the EDC system. Qualitative information is collected as part of follow-up assessments, using dedicated feedback questions, and during a separate qualitative interview study.

#### 3.2.15. Data management and monitoring

A designated data monitoring officer oversees data handling and record keeping, including real-time validation checks within the EDC system, regular audits to verify accuracy, and ongoing personnel training. All data are encrypted during transmission and stored to ensure confidentiality and regulatory compliance. At study conclusion, the sponsor archives original source documents and electronic data for a minimum of 5 years.

#### 3.2.16. Study monitoring

A Trial Steering Committee/Data Monitoring and Ethics Committee (TSC/DMEC) provides independent oversight of data integrity, participant safety, and overall trial conduct. It consists of two independent researchers and a patient partner (EP), convening at least every 3 months, and with a sponsor representative and the chief investigator present. The TSC/DMEC can request data and information for review, including the possibility of interim analyses. In addition, sponsor and chief investigator convene monthly.

#### 3.2.17. Risk and harms reporting

Although the components of the NeuOst intervention are considered safe, the study is classified as of somewhat higher risk than standard medical care because of limited experience with the intervention in the study population.

All adverse events (AEs) are recorded, regardless of severity or perceived relationship to the intervention. Trial providers and outcome assessors actively inquire about AEs at each visit/assessment, and participants are encouraged to report unusual symptoms at any time via the study phone or e-mail. Suspected serious adverse events (SAEs) are immediately reported to the chief investigator. The chief investigator then reports them to the sponsor and the TSC/DMEC within 24 h. The sponsor is responsible for reporting any Suspected Unexpected Serious Adverse Reactions (SUSARs) to relevant regulatory authorities.

#### 3.2.18. Auditing

No external or internal audits are planned, beyond auditing of the EDC for quality assurance and review by the TSC/DMEC.

#### 3.2.19. Statistical methods

A detailed statistical analysis plan (SAP) was developed prior to enrollment, focusing on descriptive statistics for primary (feasibility) outcomes and using pre-specified progression rules for benchmarking (https://osf.io/jyftb). Secondary analyses will explore potential clinical changes, focusing on estimating the variance of effects rather than hypothesis testing. Mean changes from baseline to follow-up for each outcome and group will be presented with several confidence intervals (75% to 95%), illustrated as forest plots with minimally important clinical differences marked. Analyses will be conducted using both intention-to-treat (ITT) and per-protocol approaches. Sensitivity analyses, adjusting for baseline values and potential confounders, will assess the robustness of the findings.

#### 3.2.20. Open science and data access

A full protocol and SAP were registered on the Open Science Framework (OSF (https://osf.io/jyftb)). The final, anonymized dataset will also be shared on OSF.io.

#### 3.2.21. Ethical considerations

##### 3.2.21.1. Payments

Participants are reimbursed up to £11.40 for travel per clinical visit and receive £15 per completed study assessment (i.e., for baseline and follow-up assessments, not for attending clinic visits).

##### 3.2.21.2. Confidentiality and data protection

Data are coded and de-identified, access restricted to authorized personnel, and encryption used during transmission and storage.

#### 3.2.22. Ancillary and post-trial care

While no specific post-trial clinical care is offered due to the feasibility nature of the study, ‘aftercare’ includes communicating findings to participants, disclosing group allocation, inviting them to a qualitative interview study, and exploring the potential for a subsidized community clinic offering the intervention, contingent upon feasibility, funding, and the sponsor’s strategy.

#### 3.2.23. Protocol amendments

The chief investigator can make non-substantial amendments at any time in agreement with the sponsor. Substantial amendments require formal review and approval by the research ethics committee. All amendments will be added to the protocol registration.

#### 3.2.24. Dissemination policy

Dissemination prioritizes open science practices by disseminating findings through peer-reviewed open-access publications, open-access platforms, conference presentations, lay summaries and discussion opportunities for participants and the public, and targeted communication to relevant organizations.

## 4. Discussion and limitations

This project represents an attempt to develop and test a novel multimodal intervention for people living with pDPN, addressing fundamental care gaps [18,19,69] and research priorities [22–24,70]. In line with complex intervention development frameworks [31], this feasibility trial will inform subsequent research steps and possibly further intervention refinement.

Key areas for critical appraisal upon completion of this feasibility trial include the potential for sampling bias, as individuals responding to recruitment calls for research on “a multimodal manual therapy-based intervention” may not represent the broader community’s attitudes toward physical, psychological, and self-management interventions. Generalizability may be further constrained by the single-center design and the trial’s setting in a university-affiliated teaching clinic. However, locating the study in central London is hoped to facilitate engagement with a diverse population. Intervention development may have been enhanced by involving more patient partners and clinicians to increase diversity. Given the limited experience of study clinicians with diabetic neuropathy prior to this trial, fidelity assessments will have to evaluate if provider training was sufficient. To be delivered by members from other professions or in public healthcare settings, the intervention may also require adaptation in the future.

Strengths of this study include the comprehensive approach to intervention development, including formulation of a detailed and testable theoretical model, the evidence- and stakeholder-informed selection of initial intervention components, and collaboration within a multidisciplinary expert working group. The feasibility trial design notably features a dedicated control intervention, developed according to best-practice guidelines [45]. If demonstrated to effectively blind participants to group allocation, this approach could set a higher standard for efficacy testing in complex intervention trials. This is especially relevant for studying psychological intervention components, where such a control intervention represents a novel approach [71]. The additional inclusion of a usual care group can further contextualize findings [45,72].

## 5. Conclusion

A novel intervention for people living with pDPN has been developed. A feasibility RCT will inform intervention refinement and research progression.

## Supplementary Material

Supplemental Material

## Data Availability

A full protocol and SAP were registered on the Open Science Framework (https://osf.io/jyftb). The final, anonymized dataset of the feasibility RCT will also be shared on OSF.io.

## Article highlights

Background and rationale:

- Current interventions for people experiencing painful diabetic neuropathy are limited.
- Physical, psychological, and self-management approaches are promising but require further study and may have to be combined to maximize effects.

Methods:

- A multimodal intervention was developed with stakeholder input and drawing on relevant complex intervention development frameworks.

Intervention:

- The NeuOst intervention combined manual therapy, exercises, and psychologically informed pain management strategies.

Trial protocol:

- A protocol for a 3-arm single-site feasibility randomized controlled trial is presented, aiming to randomize 36 participants with painful diabetic neuropathy to 5 sessions of NeuOst – delivered by osteopaths in an academic setting −5 sessions of a dedicated control intervention aimed at blinding and balancing of context effects or to usual care only.
- The trial’s main objectives are to test intervention feasibility and acceptability and feasibility of trial processes.

Discussion and conclusions:

- This feasibility trial is limited by being conducted in a single academic setting, but it will inform intervention refinement and research progression.
