# Supplementary material for: A multimodal intervention of manual therapy, exercise, and psychological management for painful diabetic neuropathy: intervention development and feasibility trial protocol
Source: Pain Manag. 2025 Jun 11;15(7):387–99. doi: 10.1080/17581869.2025.2515010 (PMC12218422; doi:10.1080/17581869.2025.2515010)
Supplement: Supplemental Material [file IPMT_A_2515010_SM6913.zip › suppl_data/S1 Control_Intervention_NeuOst_Concept_and_Protocol_V1.0_15_March_2024_for_PM.docx]

A multimodal manual therapy-based intervention for people with painful diabetic neuropathy:

Development and description of the control intervention (incl. treatment manual used during feasibility trial)


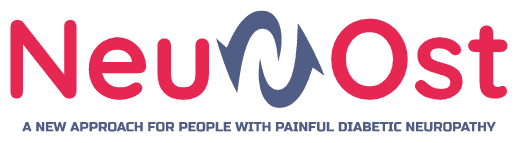


**Authors and collaborators**

David Hohenschurz-Schmidt* and the NeuOst working group

*** Chief investigator:**

Imperial College London, London, UK

University College of Osteopathy, London, UK

V.1.0, 15 March 2024

**Table of contents**

[Reporting of control intervention 3](#_Toc191044845)

[Conceptual background 3](#_Toc191044846)

[Control intervention objectives 3](#_Toc191044847)

[Control intervention rationale and development 3](#_Toc191044848)

[Provider input during co-development 4](#_Toc191044849)

[Detailed description of control intervention 4](#_Toc191044850)

[Matched components 4](#_Toc191044851)

[Components of interest 5](#_Toc191044852)

[Manual therapy components: 5](#_Toc191044853)

[Psychologically informed approaches: 6](#_Toc191044854)

[Physical exercise: 6](#_Toc191044855)

[Protocol boundaries of control intervention 7](#_Toc191044856)

[Communication scripts 7](#_Toc191044857)

[How to introduce the therapeutic objectives and structure of the trials’ interventions. (Session 1, protocol point 3) 7](#_Toc191044858)

[How to give generic exercise advice in the control arm. 7](#_Toc191044859)

[What if patients want more specific advice on exercises in the control arm? 8](#_Toc191044860)

[What if a patient asks for a more forceful or larger range of motion approach during the manual therapy component of the control intervention? 8](#_Toc191044861)

[What if a patient asks why we're talking about a certain topic or performing a certain hands-on technique? 8](#_Toc191044862)

[Providers and provider training 8](#_Toc191044863)

[Fidelity monitoring in control intervention arm 10](#_Toc191044864)

[Control intervention protocol per trial session 11](#_Toc191044865)

[References 26](#_Toc191044866)

# Reporting of control intervention

Here, the specifically designed control intervention (sometimes also called a ‘sham’ or ‘placebo’ intervention) is reported in alignment with the Recommendations for the Development, Implementation, and Reporting of Control Interventions in Efficacy and Mechanistic trials of Physical, Psychological, and Self-management Therapies (the CoPPS Statement) (Hohenschurz-Schmidt et al., 2023).

# Conceptual background

## Control intervention objectives

The objectives of the control intervention are to replicate the contextual elements of the test intervention (in other words, allow the study of the efficacy of intervention components of interest), and to blind participants to group allocation (thereby balancing expectancy and conditioning effects). The development of the control intervention follows the CoPPS Statement (Hohenschurz-Schmidt et al., 2023), and included co-development with practitioners and patient partners. As per CoPPS guidance, the control intervention resembles the tested intervention in all aspects but for the components whose effect the trial – or in this case a future full-scale trial - is designed to study.

# Control intervention rationale and development

The control intervention was developed upon completion of the NeuOst intervention’s development, following current best-practice guidance for control interventions in mechanistic and efficacy trials (Hohenschurz-Schmidt et al., 2023). This included conceptual discussions within the working group, and two practical piloting sessions with designated trial providers and the chief investigator and two people with lived experience.

First, the working group discussed which NeuOst treatment components were of interest to study in a full-scale efficacy trial. It was agreed that these components of interest were the ones for which there was a therapeutic rationale and mechanism of action, such as force parameters during manual therapy application, the elements of the exercise plan aimed at strengthening, stretching, and enhancing balance and cardiovascular fitness, and the elements of Acceptance and Commitment Therapy-derived techniques that aimed at enhancing cognitive flexibility. It was decided that, at this development stage, the combined effect of these intervention components was of greater scientific interest than their individual effects, but it was acknowledged that future intervention development and optimisation may wish to study isolated effects, for example in factorial designs (Collins et al., 2024; Ciolino et al., 2025). Together with working group subject experts, ideas for reducing or replacing these components of interest were explored. Components not of interest include: Attention from providers and a therapeutic relationship, fostered, for example, through the NeuOst intervention components of case history-taking, neurological and manual therapy examination, and complication screening. Disease education and lifestyle advice were also considered not of interest for study at this stage. Touch, including affective touch, and minimal joint or tissue movements are also not considered as components of interest in this study.

Secondly, following this conceptual development, people with lived experienced and designated trial provides were involved in the practical development of the control intervention. This included a one-to-one session of the chief investigator with a patient partner, where control and test interventions’ manual treatment sequences were tested practically, asking for feedback and adapting approaches based on feedback. Similarly, draft materials for exercises and psychologically-informed approaches were reviewed, including their counterparts developed for the control intervention (see below). Next, upon completion of the NeuOst provider training course, designated trial providers joined a practical development session where manual therapy protocols were adapted in line with the conceptual parameters outlined above. As part of this workshop and the training webinar detailed below, the importance of matched behaviours, verbal and non-verbal communication, and of maintaining blinding were discussed and practised in training scenarios. Finally, a practical training and refinement session with trial providers and two people with lived experience took place, where the complete intervention and control intervention protocols were practised in small groups.

## Provider input during co-development

During preparatory meetings and practice sessions with all designated tribe providers, providers also developed their own approach to delivering the controlled intervention within the pre-specified boundaries. They explored ways of delivering the control intervention that felt most authentic, credible, and acceptable to them. These approaches were then further refined in practice clinical appointments with patient partners and documented as part of a trial provider control intervention handbook. During those preparatory sessions, the trial providers also highlighted where they felt communication scripts may be useful and made proposals for their content.

# Detailed description of control intervention

The session-by-session provider manual for the control intervention is provided at the end of the document.

## Matched components

The control intervention matches the following features of the NeuOst intervention: the number, duration, and frequency of sessions; co-interventions; assessments and reassessments; follow-up contact frequency and mode; intervention standardisation and treatment protocol flexibility; fidelity monitoring; intervention tailoring (advice, communication, conversational topics, and physical areas treated are adapted to the patient in both trial arms); application modes and delivery formats; information regarding intervention efficacy; thematic content of conversations and information material (except for differences in topics concerning the Acceptance and Commitment Therapy-derived components, as detailed below); body areas addressed; physical procedures performed (except for differences in manual therapy and exercise components, as detailed below); level of participant participation; procedural steps per session; equipment or tools employed (including provision of a ‘wobble cushion’ in both study arms); treatment ritual (through the same assessment and overall sequence of events from case history conversation, physical assessment, manual components above, to advice and good-bye); treatment-specific sensory cues (except for differences in sensation resulting from altered manual therapy components, as detailed below); treatment environment; personal interactions with therapy providers; and provider characteristics, including education, professional qualifications, experience, trial-specific training, and behaviour (including empathic listening and reassuring attitude and communication) – since all providers deliver interventions in both the NeuOst treatment and the control intervention arm.

## Components of interest

Several components were decided to be ‘of interest’ during the conceptual development phase (Hohenschurz-Schmidt et al., 2023) and are thus not matched between NeuOst intervention and control intervention arms. Modifications (called ‘equivalents’ in the attached control intervention treatment manual), omitted components, and strategies to nonetheless achieve the control intervention’s objectives, are:

### Manual therapy components:

Soft tissue techniques are performed with less force, aiming to move dermal layers only (this is based on the mechanistic assumption that muscles need to be stretched for soft tissue techniques to be effective; touch-based or potential lymphatic mechanisms are therefore not controlled for with this superficial approach and may still have effects).

Peripheral joint articulation / mobilisation techniques are performed with less amplitude (not approaching the end of range of the movements and staying within 50% of the total ROM). This assumes that articulatory techniques have effects through stimulation of stretch receptors located in periarticular tendons and joint capsules (Bialosky et al., 2009), and on neural function through improving blood flow (Cook et al., 2023).

Spinal articulation / mobilisation and manipulation techniques are not performed and replaced by the above superficial movement of dermal layers in spinal and paraspinal areas. This acknowledges that mechanistic studies of manual therapy do not support the importance of specific force parameters so that any force or movement applied to axial joints is controlled for instead (Bialosky et al., 2017; Nim et al., 2025).

Active resisted muscle activation in the lower extremities is not performed.

Neurodynamic techniques are performed with less amplitude (not approaching the end of range of the movements and staying within 50% of the total ROM). This assumes that mechanical stress during ‘slider’ and ‘tensioner’ techniques mobilises the nerve within its sheath (Cook et al., 2023).

### Psychologically informed approaches:

ACT-derived approaches are modified to no longer target the supposed mechanisms but still resemble the original activity. Key mechanisms targeted in the NeuOst intervention and thus omitted in the control intervention are awareness, openness, values-driven action, and cognitive flexibility (McCracken and Morley, 2014).

Rather than exploring patients’ towards and away-moves in response to pain (the original ACT worksheet in session no. 1), patients are asked to reflect on their pain experience and explore pain management strategies they can do by themselves or with the help of others; Metaphors are modified to no longer be relevant to creating ‘openness’ but resemble the original ACT metaphor. For example, the ‘letting go’ metaphor of trying to push a beach ball underneath the water is replaced with a reflection on different strategies of getting the ball out of the water.

Awareness exercises (‘notice five things about your body’) are modified to ‘remember five things you can do when living with diabetes’. (both activities are from session 2).

In session 3, instead of exploring values and goals and setting a value-based SMART goal, patients are asked to remember 3-5 good experiences they had while experiencing pDPN symptoms. They are then invited to remember those while reflecting on their bodily sensations and emotions.

In session 4, instead of a mindfulness exercise to practice ‘awareness’, patients are asked to list symptoms they ascribe to their neuropathy. Together with the provider, they then explore possible pathophysiological explanations for those symptoms.

In the final session, instead of discussing how to plan for continued practice of the acquired skills, patients are asked to recapitulate the previous sessions and consider how they can “move forward from here”, which barriers they might encounter, and how they could deal with those. Instead of providing guidance or advice, providers simply engage in reflective questioning.

In the intervention arm, providers can integrate interoceptive, mindfulness, rhythmic breathing, or other relaxation exercises into their manual therapy treatment. In control intervention arm, they should limit themselves to encouraging patients to report what they are feeling or how pleasant or unpleasant a certain touch or body area feels.

### Physical exercise:

Anaerobic stretch-and-strengthen programme: In the control intervention arm, participants are given unspecific advice and encouragement to “stretch and strengthen more, for example simply getting up out of chairs repeatedly or stretching the calves against a wall”. Exercises may be demonstrated briefly but are not rehearsed.

Aerobic activity is encouraged through unspecific advice and encouragement, such as “try to go for walks more often”, “try to take the stairs instead of the elevator if possible”, or “try to break a sweat more often”.

Apart from this, and instead of a specific training programme, a generic leaflet on the potential benefits of exercise is provided, including some exercise examples but no detailed programme. During treatment sessions, exercise compliance is not followed up beyond a simple verbal enquiry from the provider, and control intervention participants have no exercise diary to self-monitor their activities. (Note that physical activity levels are still monitored as part of the study’s data collection in all study arms.)

## Protocol boundaries of control intervention

Both in the intervention and in the controlled intervention arm, the limits of what is permissible are established by the respective session by session protocols. Only obligatory or optional components listed in those protocols are within bounds.

It is noteworthy that treatment provider’s behaviour is not defined by the protocol, beyond ‘active listening’ and due to the use of an ACT-informed provider ‘stance’, both of which require the demonstration of compassion and a non-judgmental manner. Providers are required to employ the same verbal and nonverbal communication techniques and interpersonal behaviour in both the intervention and the controlled intervention arm.

Since the control intervention aims to produce the same level of positive expectations as the tested intervention, it is permissible to promote positive expectancy in the control intervention arm, albeit only to the level of what the provider also does in the intervention arm. In general, providers are encouraged to abstain from overly assured promises, which may be unbelievable to patients and are also not required since this is a research study where a certain level of uncertainty is presumed. However, positive framing, verbal and nonverbal encouragement, and talking about potential positive outcomes or desirable objectives of techniques and activities, can be used to produce positive and realistic expectations.

## Communication scripts

### How to introduce the therapeutic objectives and structure of the trials’ interventions. (Session 1, protocol point 3)

*With this treatment, we are hoping to help you to better manage your diabetic neuropathy symptoms and cope with them better. This treatment will involve 5 appointments with me, where we will talk about your symptoms but also about how they affect you and maybe about different ways you can look at them or deal with them. We will talk about different things that are important when you live with diabetic neuropathy, although I'm sure you'll be already aware of many of these issues. In each of those treatment sessions, you'll also have hands on treatment from me, where I mainly work on your legs and maybe back to help relieve some of your symptoms. We'd also be talking about the importance of physical activity and exercise from time to time. Before we start do you have any questions?*

### How to give generic exercise advice in the control arm.

*We've discussed the importance of moving for diabetes management and possibly also to stop the progression of neuropathy symptoms. It might be good to try and increase your activity levels somewhat, for example by doing some cycling, going for a walk as a higher speed than usual, or doing some exercise at home or in the gym. Maybe try that until next week?*

### What if patients want more specific advice on exercises in the control arm?

Briefly demonstrate a simple calf stretch and/or hamstring stretch. If required, give generic advice, such as: *these exercises should be held for a few moments and performed regularly*. You can also add: *with regards to exercise that gets you breathless, you could for example a few flights of stairs go for a first walk around the block or do some dancing or running if you can. It really depends on what you like to do, the important thing is that you get your heart rate up and maybe break a little sweat.*

### What if a patient asks for a more forceful or larger range of motion approach during the manual therapy component of the control intervention?

*There are many different osteopathic techniques, some of which are quite forceful, while others are quite gentle and more specific. This research study aims to investigate the more subtle approaches, which is why I'm not able to change what I do with my hands too much. I am however choosing my techniques depending on what I feel where it might be useful to treat you. I'm sure this will also be beneficial to you.*

### What if a patient asks why we're talking about a certain topic or performing a certain hands-on technique?

Here it is important to provide an explanation. Usually these can be held generic and borrowed from the rationale in the intervention arm. For example, when performing the control intervention’s ACT activities, you may wish to point out that talking about one’s experience can produce important new insights and acknowledge that it might not always feel comfortable at first. For the control intervention manual therapy techniques, one could explain that they aim to provide sensory input, relaxation, and movement, which may or may not reduce discomfort or enable patients to move better.

# Providers and provider training

For designated trial providers, the NeuOst training course (see NeuOst intervention protocol) included two obligatory lectures on 1) the Fundamentals of Nonpharmacological Intervention Research and of Placebo Controlled Trials, and 2) the NeuOst Trial Protocol and Considerations for Trial Providers, delivered by the chief investigator. This covered the following learning objectives and content. Course completion was ensured by the chief investigator and understanding was tested by means of a quiz.

Talk 1: The Fundamentals of Nonpharmacological Intervention Research and of Placebo Controlled Trials (Duration: 0.5 hours, Delivery mode: Recorded)

Learning objectives:

- Appreciate the challenges of nonpharmacological / complex intervention clinical research, especially in so-called efficacy trials.
- Appreciate the potential for placebo effects in physical and psychological interventions and their implications for trial design.
- Understand the concepts of blinding and control interventions in efficacy trials.
- Know about current best-practice frameworks for control intervention design.

Content:

- 30-mins recording of a webinar held for the British Pain Society (full recording: <https://www.youtube.com/watch?v=WI4jaqf5_PE>)
- Webinar includes an optional 30-mins talk by Prof David Beard on placebo-controlled surgery trials and a panel discussion.

Talk 2: The NeuOst Trial Protocol and Considerations for Trial Providers (Duration: 0.5 hours, Delivery mode: Synchronous e-learning - live delivery after in-practice training day for selected trial providers)

Learning objectives:

- Understand the basic design elements NeuOst feasibility trial.
- Appreciate the tasks and role of clinical providers in an RCT in general and in NeuOst in particular.
- Reflect on and discuss concerns and challenges as trial providers.
- Gain access to materials for providers, such as detailed session protocol and ACT handbook.
- Commence practical feasibility trial participation planning, such as populating weekly diaries.

Content:

- Key design elements of the NeuOst feasibility trial
- Role and responsibilities of clinical trial providers in sham-controlled trials in general and in the NeuOst feasibility trial in particular.
- Experiences of trial providers from relevant related RCTs.
- Relevant ethical considerations.
- Reflection and discussion amongst participants.
- Training tasks.

Finally, designated trial providers attended a practical 2-hour session with two patient partners, where the complete intervention and control intervention protocols were practised in small groups. Afterwards, providers reflected on the experience, facilitated by the chief investigator.

In between individual training sessions, providers were encouraged to revisit theoretical materials and practice their NeuOst-related skills with their own patients, friends, or colleagues. Trial processes and (control) intervention delivery were refined based on experiences during this work with designated trial providers. This included the development of conversation scripts for clinical appointments (see above). As part of this preparation, trial providers were also trained to recognise the need for a control intervention and maintaining participant blinding in this research study. During the trial, trial providers communicate with one another about challenges in control intervention delivery and attend regular supervision and troubleshooting sessions with the chief investigator and the working group’s clinical psychologist.

# Fidelity monitoring in control intervention arm

Fidelity monitoring processes are the same as in the intervention arm (NeuOst feasibility trial protocol section 8.13), except that the below (control) intervention protocol will be used for fidelity checking of audio- and video recordings and for provider self-report of delivered intervention components.

# Control intervention protocol per trial session

| **Control Intervention content** (providers decide the sequence per session)  Components marked ‘optional’ are non-obligatory; all others must be delivered as part of the respective treatment session. | Fidelity checklist  (to be completed by trial providers) | **Notes** (Further detail such as amount and duration or provider experiences such as problems and opportunities) |
| --- | --- | --- |
| Session 1 |  | Session duration up to 90 mins |
| Communication elements:   1. Active listening to patient narrative 2. Initial case history 3. Introduction to the therapeutic objectives and structure of NeuOst 4. Acknowledgement of medication list 5. Conversation about medications (optional) 6. Communication with primary care / specialist care provider (optional) 7. Signposting to DM/NeuP-related care options (optional) 8. Exploration of patient beliefs (regarding pain and disease) (optional) 9. Education about pain and neuropathic pain, including management strategies, sleep management, and devices (optional)   ACT-informed elements:   1. Exploration of possible self-help and external support strategies (via Session 1 ACT worksheet) 2. Exploration of patient feelings (via Session 1 ACT worksheet) 3. Explorations of additional, personally-relevant self-help or support strategies (via Session 1 ACT worksheet) | Comms:              ACT: | Up to 30 mins |
| Manual therapy:   1. **Assessment** (inspection, active & passive joint movement, palpation) 2. **Peripheral joint articulation equivalent** (feet & ankles at least 2mins per side; then knees & hips any duration) 3. Other peripheral joint articulation equivalents (optional) 4. Spinal joint articulation equivalents (optional) 5. Peripheral soft tissue manipulation equivalent (gastrocnemius at least 1min per side) 6. Other peripheral soft tissue manipulation equivalents (optional) 7. Paraspinal soft tissue manipulation equivalents (optional) 8. **Active muscle activation** (lower extremity: dorsiflexion, plantarflexion, knee flexion and knee extension, at least 10 times each per side, hold 5 seconds per rep.) 9. Neurodynamic technique lower extremity equivalent (active; 10 reps per side) 10. ‘Subtle’ osteopathic technique equivalents (optional) 11. Integration of patient breathing, relaxation, interoceptive awareness (“notice” and “open”), and/or activity-exploration (ACT- equivalents based) into manual treatment (optional) | MT: | Up to 20 mins |
| Neurological:   1. **Provider baseline neurological examination** (in addition to research-related baseline testing; not used as outcome) | Neuro: | Up to 10 mins |
| Physical activity:   1. Raise the topic of physical activity with permission (use motivational interviewing techniques throughout) (5As’: “Ask”) 2. Evaluation of current physical activity levels (“Assess”) 3. Explore barriers to more engagement in physical activity 4. Educate about benefits of different types of physical activity (“Advise”) 5. **Provide “Exercise and diabetes” handout** (by the Diabetes Research & Wellness Foundation) (“Advise”) 6. **Values-based goal setting for aerobic exercise activity** (patient choice of activity, use Borg scale for exertion level) (“Agree”) 7. Give general advice on possible stretch, strength, or stability exercises. 8. Decide on suitable modifications for exercises (optional) 9. **Provide “wobble cushion”** (“Arrange”) (provided as part of trial) | Exx: | Up to 20 mins |
| Diabetes related:   1. **Diabetes complication screening** (red flags for foot health, vision changes, kidney disease etc.) 2. Conversation about dietary DM management (optional) 3. Education about DM (optional) 4. Education about DPN and other complications of DM, including management strategies (optional) 5. Provision of NeuOst educational materials (optional) | DM/DPN: | Up to 10 mins |
|  |  |  |
| PROVIDERS TO COMPLETE AT END OF SESSION:  Please rate your agreement with the following statement with regards to today’s intervention session for the management of the patient’s painful diabetic neuropathy symptoms.  This treatment will be completely effective.     \| Strongly disagree \| Moderately disagree \| Slightly disagree \| Neither agree nor disagree \| Slightly agree \| Moderately agree \| Strongly agree \| \| --- \| --- \| --- \| --- \| --- \| --- \| --- \| \|  \|  \|  \|  \|  \|  \|  \| | | |

| Session 2 |  | 45-60 mins duration |
| --- | --- | --- |
| Communication:   1. Active listening to patient narrative 2. Follow-up case history 3. Conversation about medications (optional) 4. Communication with primary care / specialist care provider (optional) 5. Signposting to DM/NeuP-related care options (optional) 6. Exploration of patient beliefs (regarding pain and disease) (optional) 7. Education about pain and neuropathic pain, including management strategies, sleep management, and devices (optional)   ACT-informed elements:   1. Revisiting last week’s exploration of self-help and external help strategies 2. Exploring the problem with doing it alone “Beach ball metaphor” (via Session 2 ACT worksheet) 3. “5 things to do when you live with diabetes” exercise (via Session 2 ACT worksheet) 4. Encouragement to practice these activities at home |  | Up to 20 mins |
| Manual therapy:   1. **Assessment** (inspection, active & passive joint movement, palpation) 2. **Peripheral joint articulation equivalent** (feet & ankles at least 2mins per side; then knees & hips any duration) 3. Other peripheral joint articulation equivalents (optional) 4. Spinal joint articulation equivalents (optional) 5. Peripheral soft tissue manipulation equivalent (gastrocnemius at least 1min per side) 6. Other peripheral soft tissue manipulation equivalents (optional) 7. Paraspinal soft tissue manipulation equivalents (optional) 8. **Active muscle activation** (lower extremity: dorsiflexion, plantarflexion, knee flexion and knee extension, at least 10 times each per side, hold 5 seconds per rep.) 9. Neurodynamic technique lower extremity equivalent (active; 10 reps per side) 10. ‘Subtle’ osteopathic technique equivalents (optional) 11. Integration of patient breathing, relaxation, interoceptive awareness (“notice” and “open”), and/or activity-exploration (ACT- equivalents based) into manual treatment (optional) | MT: | Up to 20 mins |
| Neurological:   1. Follow-up neurological examination (if indicated, optional) |  |  |
| Physical activity:   1. Revisit experiences and/or exercises of performed / advised at last session 2. Explore if exercises and stretches can now be performed at greater difficulty level or through larger range of movement 3. Reinforce importance of exercise (optional) 4. Explore barriers to adherence and provide advice on overcoming those (optional) 5. Decide on suitable modifications for exercise activities (optional) |  | Up to 10 mins |
| Diabetes management and education:   1. Foot care education and practical run-through (under guidance of below handout) 2. **Provide foot care handout** (“Diabetes and looking after your feet” from Diabetes UK) 3. Conversation about dietary DM management (optional) 4. Education about DM (optional) 5. Education about DPN and other complications of DM, including management strategies (optional) 6. Provision of other NeuOst educational materials (optional) |  | Up to 10 mins |
|  |  |  |
| PROVIDERS TO COMPLETE AT END OF SESSION:  Please rate your agreement with the following statement with regards to today’s intervention session for the management of the patient’s painful diabetic neuropathy symptoms.  This treatment will be completely effective.     \| Strongly disagree \| Moderately disagree \| Slightly disagree \| Neither agree nor disagree \| Slightly agree \| Moderately agree \| Strongly agree \| \| --- \| --- \| --- \| --- \| --- \| --- \| --- \| \|  \|  \|  \|  \|  \|  \|  \| | | |

| Session 3 |  | 45-60 mins duration |
| --- | --- | --- |
| Communication:   1. Active listening to patient narrative 2. Follow-up case history 3. Conversation about medications (optional) 4. Communication with primary care / specialist care provider (optional) 5. Signposting to DM/NeuP-related care options (optional) 6. Exploration of patient beliefs (regarding pain and disease) (optional) 7. Education about pain and neuropathic pain, including management strategies, sleep management, and devices (optional)   ACT-informed elements:   1. Reviewing patient experiences with “5 Things to do when you have diabetes” exercise 2. Providing information on positive experiences (via Session 3 ACT worksheet) 3. Completing “Experience Compass” and planning on recreating positive experiences (Session 3 ACT worksheet) |  | Up to 30 mins |
| Manual therapy:   1. **Assessment** (inspection, active & passive joint movement, palpation) 2. **Peripheral joint articulation equivalent** (feet & ankles at least 2mins per side; then knees & hips any duration) 3. Other peripheral joint articulation equivalents (optional) 4. Spinal joint articulation equivalents (optional) 5. Peripheral soft tissue manipulation equivalent (gastrocnemius at least 1min per side) 6. Other peripheral soft tissue manipulation equivalents (optional) 7. Paraspinal soft tissue manipulation equivalents (optional) 8. **Active muscle activation** (lower extremity: dorsiflexion, plantarflexion, knee flexion and knee extension, at least 10 times each per side, hold 5 seconds per rep.) 9. Neurodynamic technique lower extremity equivalent (active; 10 reps per side) 10. ‘Subtle’ osteopathic technique equivalents (optional)   Integration of patient breathing, relaxation, interoceptive awareness (“notice” and “open”), and/or activity-exploration (ACT- equivalents based) into manual treatment (optional) | MT: | Up to 20 mins |
| Neurological:   1. Follow-up neurological examination (if indicated, optional) |  |  |
| Physical activity:   1. Revisit experiences and/or exercises of performed / advised at last session 2. Explore if exercises and stretches can now be performed at greater difficulty level or through larger range of movement 3. Reinforce importance of exercise (optional) 4. Explore barriers to adherence and provide advice on overcoming those (optional) 5. Decide on suitable modifications for exercise activities (optional) |  | Up to 10 mins |
| Diabetes management and education:   1. Revisit content and experiences with foot care education and handout 2. Conversation about dietary DM management (optional) 3. Education about DM (optional) 4. Education about DPN and other complications of DM, including management strategies (optional) 5. Provision of other NeuOst educational materials (optional) |  | Up to 10 mins |
|  |  |  |
| PROVIDERS TO COMPLETE AT END OF SESSION:  Please rate your agreement with the following statement with regards to today’s intervention session for the management of the patient’s painful diabetic neuropathy symptoms.  This treatment will be completely effective.     \| Strongly disagree \| Moderately disagree \| Slightly disagree \| Neither agree nor disagree \| Slightly agree \| Moderately agree \| Strongly agree \| \| --- \| --- \| --- \| --- \| --- \| --- \| --- \| \|  \|  \|  \|  \|  \|  \|  \| | | |

| Session 4 |  | 45-60 mins duration |
| --- | --- | --- |
| Communication:   1. Active listening to patient narrative 2. Follow-up case history 3. Conversation about medications (optional) 4. Communication with primary care / specialist care provider (optional) 5. Signposting to DM/NeuP-related care options (optional) 6. Exploration of patient beliefs (regarding pain and disease) (optional) 7. Education about pain and neuropathic pain, including management strategies, sleep management, and devices (optional)   ACT-informed elements:   1. Revisiting last week’s positive experiences plan (incl. any progress or barriers) 2. Conducting the “Neuropathic Pain Educational exercise” (via Session 4 ACT worksheet) |  | Up to 20 mins |
| Manual therapy:   1. **Assessment** (inspection, active & passive joint movement, palpation) 2. **Peripheral joint articulation equivalent** (feet & ankles at least 2mins per side; then knees & hips any duration) 3. Other peripheral joint articulation equivalents (optional) 4. Spinal joint articulation equivalents (optional) 5. Peripheral soft tissue manipulation equivalent (gastrocnemius at least 1min per side) 6. Other peripheral soft tissue manipulation equivalents (optional) 7. Paraspinal soft tissue manipulation equivalents (optional) 8. **Active muscle activation** (lower extremity: dorsiflexion, plantarflexion, knee flexion and knee extension, at least 10 times each per side, hold 5 seconds per rep.) 9. Neurodynamic technique lower extremity equivalent (active; 10 reps per side) 10. ‘Subtle’ osteopathic technique equivalents (optional)   Integration of patient breathing, relaxation, interoceptive awareness (“notice” and “open”), and/or activity-exploration (ACT- equivalents based) into manual treatment (optional) | MT: | Up to 20 mins |
| Neurological:   1. Follow-up neurological examination (if indicated, optional) |  |  |
| Physical activity:   1. Revisit experiences and/or exercises of performed / advised at last session 2. Explore if exercises and stretches can now be performed at greater difficulty level or through larger range of movement 3. Reinforce importance of exercise (optional) 4. Explore barriers to adherence and provide advice on overcoming those (optional) 5. Decide on suitable modifications for exercise activities (optional) |  | Up to 10 mins |
| Diabetes management and education:   1. Conversation about dietary DM management (optional) 2. Education about DM (optional) 3. Education about DPN and other complications of DM, including management strategies (optional) 4. Provision of other NeuOst educational materials (optional) |  | Up to 10 mins |
|  |  |  |
| PROVIDERS TO COMPLETE AT END OF SESSION:  Please rate your agreement with the following statement with regards to today’s intervention session for the management of the patient’s painful diabetic neuropathy symptoms.  This treatment will be completely effective.     \| Strongly disagree \| Moderately disagree \| Slightly disagree \| Neither agree nor disagree \| Slightly agree \| Moderately agree \| Strongly agree \| \| --- \| --- \| --- \| --- \| --- \| --- \| --- \| \|  \|  \|  \|  \|  \|  \|  \| | | |

| Session 5 |  | 45-60 mins duration |
| --- | --- | --- |
| Communication:   1. Active listening to patient narrative 2. Follow-up case history 3. Conversation about medications (optional) 4. Communication with primary care / specialist care provider (optional) 5. Signposting to DM/NeuP-related care options (optional) 6. Exploration of patient beliefs (regarding pain and disease) (optional) 7. Education about pain and neuropathic pain, including management strategies, sleep management, and devices (optional)   ACT-informed elements:   1. Exploring thoughts on the Neuropathic Pain education since last session 2. **Reviewing any skills practice patient may be performing** (incl. impact on life, opportunities for further practice, barriers and how to manage those moving forward) 3. Review session 3 “things to do when living with diabetes” and “positive experiences” activities (incl. any progress towards that) 4. Discussing how to deal with difficulties |  | Up to 20 mins |
| Manual therapy:   1. **Assessment** (inspection, active & passive joint movement, palpation) 2. **Peripheral joint articulation equivalent** (feet & ankles at least 2mins per side; then knees & hips any duration) 3. Other peripheral joint articulation equivalents (optional) 4. Spinal joint articulation equivalents (optional) 5. Peripheral soft tissue manipulation equivalent (gastrocnemius at least 1min per side) 6. Other peripheral soft tissue manipulation equivalents (optional) 7. Paraspinal soft tissue manipulation equivalents (optional) 8. **Active muscle activation** (lower extremity: dorsiflexion, plantarflexion, knee flexion and knee extension, at least 10 times each per side, hold 5 seconds per rep.) 9. Neurodynamic technique lower extremity equivalent (active; 10 reps per side) 10. ‘Subtle’ osteopathic technique equivalents (optional) 11. Integration of patient breathing, relaxation, interoceptive awareness (“notice” and “open”), and/or activity-exploration (ACT- equivalents based) into manual treatment (optional) | MT: | Up to 20 mins |
| Neurological:   1. Follow-up neurological examination (this week not optional)   (for providers; not used as outcome during research study) |  | Up to 15 mins |
| Physical activity:   1. Revisit experiences and/or exercises of performed / advised at last session 2. Explore if exercises and stretches can now be performed at greater difficulty level or through larger range of movement 3. Reinforce importance of exercise (optional) 4. Explore barriers to adherence and provide advice on overcoming those (optional) 5. Decide on suitable modifications for exercise activities (optional) |  | Up to 10 mins |
| Diabetes management and education:   1. Explore patient’s plans for future DM and painful DPN management 2. Conversation about dietary DM management (optional) 3. Education about DM (optional) 4. Education about DPN and other complications of DM, including management strategies (optional) 5. Provision of other NeuOst educational materials (optional) |  | Up to 10 mins |
|  |  |  |
| PROVIDERS TO COMPLETE AT END OF SESSION:  Please rate your agreement with the following statement with regards to today’s intervention session for the management of the patient’s painful diabetic neuropathy symptoms.  This treatment will be completely effective.     \| Strongly disagree \| Moderately disagree \| Slightly disagree \| Neither agree nor disagree \| Slightly agree \| Moderately agree \| Strongly agree \| \| --- \| --- \| --- \| --- \| --- \| --- \| --- \| \|  \|  \|  \|  \|  \|  \|  \| | | |
| End of programme | | |

# References

Bialosky, J.E., Beneciuk, J.M., Bishop, M.D., Coronado, R.A., Penza, C.W., Simon, C.B., George, S.Z., 2017. Unraveling the Mechanisms of Manual Therapy: Modeling an Approach. J Orthop Sports Phys Ther 48, 8–18. https://doi.org/10.2519/jospt.2018.7476

Bialosky, J.E., Bishop, M.D., Price, D.D., Robinson, M.E., George, S.Z., 2009. The mechanisms of manual therapy in the treatment of musculoskeletal pain: A comprehensive model. Manual Therapy 14, 531–538. https://doi.org/10.1016/j.math.2008.09.001

Ciolino, J.D., Scholtens, D.M., Bonner, L.B., 2025. Factorial Clinical Trial Designs. JAMA 333, 532–533. https://doi.org/10.1001/jama.2024.25374

Collins, L.M., Nahum-Shani, I., Guastaferro, K., Strayhorn, J.C., Vanness, D.J., Murphy, S.A., 2024. Intervention Optimization: A Paradigm Shift and Its Potential Implications for Clinical Psychology. Annual Review of Clinical Psychology 20, 21–47. https://doi.org/10.1146/annurev-clinpsy-080822-051119

Cook, C.E., Rhon, D.I., Bialosky, J., Donaldson, M., George, S.Z., Hall, T., Kawchuk, G., Lane, E., Lavazza, C., Lluch, E., Louw, A., Mazzieri, A.M., McDevitt, A., Reed, W.R., Schmid, A.B., Silva, A.G., Smart, K.M., Puentedura, E.J., 2023. Developing Manual Therapy Frameworks for Dedicated Pain Mechanism. JOSPT Open 1, 48–62. https://doi.org/10.2519/josptopen.2023.0002

Hohenschurz-Schmidt, D., Vase, L., Scott, W., Annoni, M., Ajayi, O.K., Barth, J., Bennell, K., Berna, C., Bialosky, J., Braithwaite, F., Finnerup, N.B., Williams, A.C. de C., Carlino, E., Cerritelli, F., Chaibi, A., Cherkin, D., Colloca, L., Côté, P., Darnall, B.D., Evans, R., Fabre, L., Faria, V., French, S., Gerger, H., Häuser, W., Hinman, R.S., Ho, D., Janssens, T., Jensen, K., Johnston, C., Lunde, S.J., Keefe, F., Kerns, R.D., Koechlin, H., Kongsted, A., Michener, L.A., Moerman, D.E., Musial, F., Newell, D., Nicholas, M., Palermo, T.M., Palermo, S., Peerdeman, K.J., Pogatzki-Zahn, E.M., Puhl, A.A., Roberts, L., Rossettini, G., Matthiesen, S.T., Underwood, M., Vaucher, P., Vollert, J., Wartolowska, K., Weimer, K., Werner, C.P., Rice, A.S.C., Draper-Rodi, J., 2023. Recommendations for the development, implementation, and reporting of control interventions in efficacy and mechanistic trials of physical, psychological, and self-management therapies: the CoPPS Statement. BMJ 381, e072108. https://doi.org/10.1136/bmj-2022-072108

McCracken, L.M., Morley, S., 2014. The Psychological Flexibility Model: A Basis for Integration and Progress in Psychological Approaches to Chronic Pain Management. The Journal of Pain 15, 221–234. https://doi.org/10.1016/j.jpain.2013.10.014

Nim, C., Aspinall, S.L., Cook, C.E., Corrêa, L.A., Donaldson, M., Downie, A.S., Harsted, S., Hansen, S., Jenkins, H.J., McNaughton, D., Nyirö, L., Perle, S.M., Roseen, E.J., Young, J.J., Young, A., Zhao, G.-H., Hartvigsen, J., Juhl, C.B., 2025. The Effectiveness of Spinal Manipulative Therapy in Treating Spinal Pain Does Not Depend on the Application Procedures: A Systematic Review and Network Meta-analysis. Journal of Orthopaedic & Sports Physical Therapy 55, 109–122. https://doi.org/10.2519/jospt.2025.12707
