# Supplementary material for: A multimodal intervention of manual therapy, exercise, and psychological management for painful diabetic neuropathy: intervention development and feasibility trial protocol
Source: Pain Manag. 2025 Jun 11;15(7):387–99. doi: 10.1080/17581869.2025.2515010 (PMC12218422; doi:10.1080/17581869.2025.2515010)
Supplement: Supplemental Material [file IPMT_A_2515010_SM6913.zip › suppl_data/S2 Intervention_protocol_NeuOst_11_March_2024 (1).docx]

**A multimodal manual therapy-based intervention for people with painful diabetic neuropathy:**

**Development and description of the NeuOst intervention**

**(incl. treatment manual used during feasibility trial)**


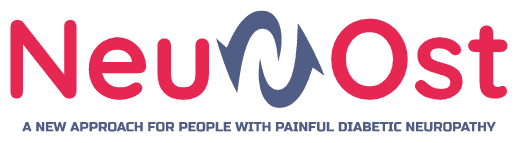


**Authors and collaborators**

David Hohenschurz-Schmidt* and the NeuOst working group

*** Chief investigator:**

Imperial College London, London, UK

University College of Osteopathy, London, UK

Version 3.0, 11 March 2024

**Table of Contents**

[Introduction 3](#_Toc191044625)

[Background and intervention rationale 3](#_Toc191044626)

[Methods of intervention development and evaluation 6](#_Toc191044627)

[Developing NeuOst according to the MRC framework for complex intervention development 6](#_Toc191044628)

[Programme flexibility and specificity 7](#_Toc191044629)

[Intervention refinement and boundaries 8](#_Toc191044630)

[Stakeholders 9](#_Toc191044631)

[Key uncertainties and research perspectives 10](#_Toc191044632)

[Intervention development 11](#_Toc191044633)

[Pathophysiological and clinical changes in painful DPN and target mechanisms of NeuOst 11](#_Toc191044634)

[Neurological mechanisms 11](#_Toc191044635)

[Musculoskeletal mechanisms 12](#_Toc191044636)

[Psychological, social, and behavioural mechanisms 13](#_Toc191044637)

[Intervention theory of NeuOst: context and components 15](#_Toc191044638)

[Context definition 15](#_Toc191044639)

[Programme theory 17](#_Toc191044640)

[Patient and public involvement 18](#_Toc191044641)

[Intervention components table 21](#_Toc191044642)

[Intervention elements per treatment session (treatment protocol for feasibility trial) 31](#_Toc191044643)

[Elaboration of individual programme components 43](#_Toc191044644)

[Manual therapy 43](#_Toc191044645)

[Physical activity and exercise 44](#_Toc191044646)

[Educational components for patients 48](#_Toc191044647)

[Psychological components 49](#_Toc191044648)

[Provider training course 51](#_Toc191044649)

[Course plan 53](#_Toc191044650)

[Aditional training for designated trial providers 59](#_Toc191044651)

[Outlook: Further phases of intervention development 61](#_Toc191044652)

[Developing NeuOst as a complex intervention 61](#_Toc191044653)

[Feasibility 61](#_Toc191044654)

[Evaluation 62](#_Toc191044655)

[Implementation 62](#_Toc191044656)

[Economic considerations 62](#_Toc191044657)

[References 64](#_Toc191044658)

# Introduction

‘Neuropathy-Oriented Skills and Therapy’ (NeuOst) is a complex intervention based on osteopathic manual therapy and incorporating elements of cognitive-behavioural, mind-body, exercise, and educational interventions.

NeuOst is being developed with a multidisciplinary group of researchers and clinicians, and with extensive stakeholder involvement. Key stakeholders are people with painful diabetic peripheral neuropathy (pDPN) as addressees of NeuOst, and osteopathic practitioners as interventionists.

## Background and intervention rationale

With an estimated prevalence of 9.3% of the adult population, diabetes mellitus (DM) is one of the leading health concerns of the modern world and projected to rise over the next decades, especially in younger people and in countries moving from lower to higher incomes (International Diabetes Federation, 2019). Estimates for associated diabetic peripheral neuropathy (DPN), which is often undiagnosed and may result in severe diabetic foot complications, range from 16% to 87% (Sobhani et al., 2014; International Diabetes Federation, 2019). In about 26% of patients, diabetes is associated with a *painful* diabetic neuropathy, contributing to disability and reduced quality of life (Davies et al., 2006) and associated with higher risk of anxiety and depression (Kioskli et al., 2019a; Kec et al., 2021). Often unrecognised is the fact that musculoskeletal pain is also up to twice as common in a diabetic population compared to healthy controls (Themistocleous et al., 2016), associated with typical chronic pain risk factors such as higher body mass index and sedentary life style (Molsted et al., 2012). Both diabetes and low back pain are also more prevalent in socioeconomically disadvantaged communities, highlighting the importance of social determinants of health (Karran et al., 2020; Hill-Briggs et al., 2021).

Nonpharmacological interventions are discussed frequently in the context of diabetes (Pandey et al., 2011; Raveendran et al., 2018), notably physical exercise, dietary interventions (Fjeldsoe et al., 2011; Davies et al., 2015; Yamamoto et al., 2018), bariatric surgery (Maggard-Gibbons et al., 2013), sleep and stress management (Hamid, 2011; Touma and Pannain, 2011; Wagner et al., 2016), and cognitive behavioural interventions (Safren et al., 2014; Castelnuovo et al., 2017). Education, lifestyle interventions, and dietary control form part of clinical guidelines alongside pharmacological therapy (McGuire et al., 2016).

In practice, the treatment and management of people *with pain* and DPN focuses on pharmacological interventions (Tölle et al., 2006; Meisinger et al., 2018), despite the limited effectiveness of many drugs and range of side effects (Dixit and Asiri, 2014; Finnerup et al., 2015; Javed et al., 2015). Contrastingly, clinical guidelines and expert consensus advocate for nonpharmacological options to be considered alongside pharmacotherapy, notably lifestyle modification, optimal diabetes treatment, and multifactorial cardiovascular risk interventions (McGuire et al., 2016; Ziegler et al., 2021). These experts also call for patient management beyond pain relief, also addressing quality of sleep, functionality, and general quality of life (Ziegler et al., 2021). Nonetheless, apart from dietary supplements and neurostimulation, there are few clinical trials investigating non-pharmacological treatments for people with painful DPN. Recent systematic reviews on neuropathic pain (Moisset et al., 2020) found high-quality studies only for mindfulness-based stress reduction in people with DPN (notably (Nathan et al., 2017)), and an absence of research into massage- or exercises-based interventions. The lack of psychological intervention trials is remarkable in light of the high comorbidity of psychological problems and the potentially mutually reinforcing nature of neuropathic pain and psychological risk factors (Kioskli et al., 2019a). A systematic review of various types of manual therapy for DPN concluded that manual therapy was effective short-term, albeit based on only six low- or mid-quality studies comparing interventions against treatment as usual (Hernández-Secorún et al., 2021). This review also found 22 studies of exercise for DPN, again reporting signs of effectiveness of various forms of exercise on functional outcomes and pain. Other reviews dedicated to non-pharmacological therapies for people with DPN (Çakici et al., 2016; Amato Nesbit et al., 2019) or painful peripheral neuropathies (Liampas et al., 2020) “*underscore[…] the lack of good quality studies […]*" (Liampas et al., 2020), concluding that *“[f]urther research should address long-term outcomes and other non-pharmacologic treatments.*" (Amato Nesbit et al., 2019).

Osteopaths and other manual therapists are familiar with treating people experiencing pain (Fawkes and Carnes, 2021). A recent estimate places the proportion of patients with diabetes in UK osteopathic practice at about 4% (Plunkett et al., 2021). Whilst the proportion of patients seeking help for or with painful DPN is unclear, speculations about a potential role of osteopathic manual therapists in the treatment and management of people with diabetes and concomitant pain exist. Treatment rationales include the facilitation of exercise and active lifestyles, the reduction of pain by treating associated neuropathy or concomitant musculoskeletal conditions, and, to a lesser degree, indirect effects on diabetes disease markers via enhanced engagement in activities (Shafer et al., 2020). Existing studies to these effects are, however, sparse and of questionable methodological quality. They include a subgroup analysis of a larger osteopathic trial showing that low back pain in patients with DM improves compared to a sham intervention; as it did in the overall sample irrespective of diabetes (Licciardone et al., 2013). Further, several small studies and student projects on disease parameters or disease complications were performed, including diabetic ulcers (King, 2016), intraocular pressure (Díaz Cerrato, 2008; Kuhmann, 2008), blood glucose (Bono Mira, 2008; Carpenter, 2016; Vermeersch and Quaghebeur, 2018), insulin levels (Pêgas de Oliveira, 2008), and HbA1c (Kiegerl, 2007), suggesting an interest in the field but not allowing for conclusions regarding treatment efficacy.

The sparsity and low quality of research into nonpharmacological treatments and management approaches for people with painful DPN is a great gap, given how common and debilitating this complication of DM is, and the inadequacy of existing drug treatments and their associated side-effects (Dixit and Asiri, 2014; Finnerup et al., 2015). With their expertise in pain management, and provided with additional training in behavioural and dietary interventions, osteopaths may be well-placed to look after people with painful DPN. Combinational or adjunct psychological approaches are increasingly explored for pain management, including psychologically-informed physiotherapy (Keefe et al., 2018). ‘Augmentations’ of osteopathy with cognitive-behavioural techniques have been successfully developed before (Carnes et al., 2017). Whilst the role of manual techniques in a pDPN population is under-researched, limited evidence suggestive of efficacy exists (Hernández-Secorún et al., 2021). It may be hypothesised that manual treatment and other therapeutic components may interact with one another and with patient characteristics to facilitate behavioural change and promote health (Underwood et al., 2007; Bishop et al., 2015, p. 2015; Bialosky et al., 2017; Keefe et al., 2018).

To enhance the applicability of osteopathy to a clinical population of people with neuropathic pain and diabetes, an intervention will have to be developed prior to testing, drawing on core osteopathic therapy components, psychologically informed approaches, and combining them with elements from other interventions relevant for people with diabetes and pain. This intervention will be called ‘Augmented osteopathic care for people with painful diabetic neuropathy’, or *NeuOst*. *NeuOst* components will be taught to fully qualified osteopaths who then deliver the intervention.

Specifically, we have assembled a collaborative network of leading research experts to explore the mechanistic rationale for manual therapy in DPN, develop the combination of osteopathy with other relevant approaches, decide on adequate trial designs for a proof-of-concept study, and feasibility test components of an efficacy final trial. Support from *The Osteopathic Foundation* (oF) will act as a seed fund to develop the intervention and a training for providers and facilitate the successful application for a large-scale trial based on reliable feasibility data.

# Methods of intervention development and evaluation

## Developing NeuOst according to the MRC framework for complex intervention development

This document describes the phases and core elements of the *NeuOst* intervention, drawing on the framework for complex intervention development and evaluation provided by the Medical Research Council (MRC) (**Figure 1**) (Skivington et al., 2021).


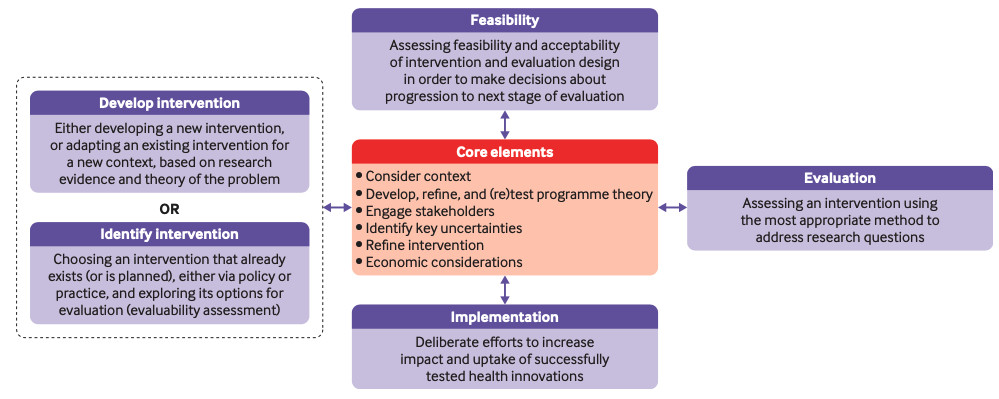


**Figure 1: MRC Framework for developing and evaluating complex interventions**. Context=any feature of the circumstances in which an intervention is conceived, developed, evaluated, and implemented; programme theory=describes how an intervention is expected to lead to its effects and under what conditions—the programme theory should be tested and refined at all stages and used to guide the identification of uncertainties and research questions; stakeholders=those who are targeted by the intervention or policy, involved in its development or delivery, or more broadly those whose personal or professional interests are affected (that is, who have a stake in the topic)—this includes patients and members of the public as well as those linked in a professional capacity; uncertainties=identifying the key uncertainties that exist, given what is already known and what the programme theory, research team, and stakeholders identify as being most important to discover—these judgments inform the framing of research questions, which in turn govern the choice of research perspective; refinement=the process of fine tuning or making changes to the intervention once a preliminary version (prototype) has been developed; economic considerations=determining the comparative resource and outcome consequences of the interventions for those people and organisations affected. (Skivington et al., 2021)

An overview of the process is provided in **Figure 2**; adapted from Craig and Campbell (2015) and focussing on the MRC framework elements “Develop Intervention”, “Core Elements”, and “Feasibility”. Methods for the feasibility evaluation will be described in more detail in a feasibility trial protocol at a later stage.


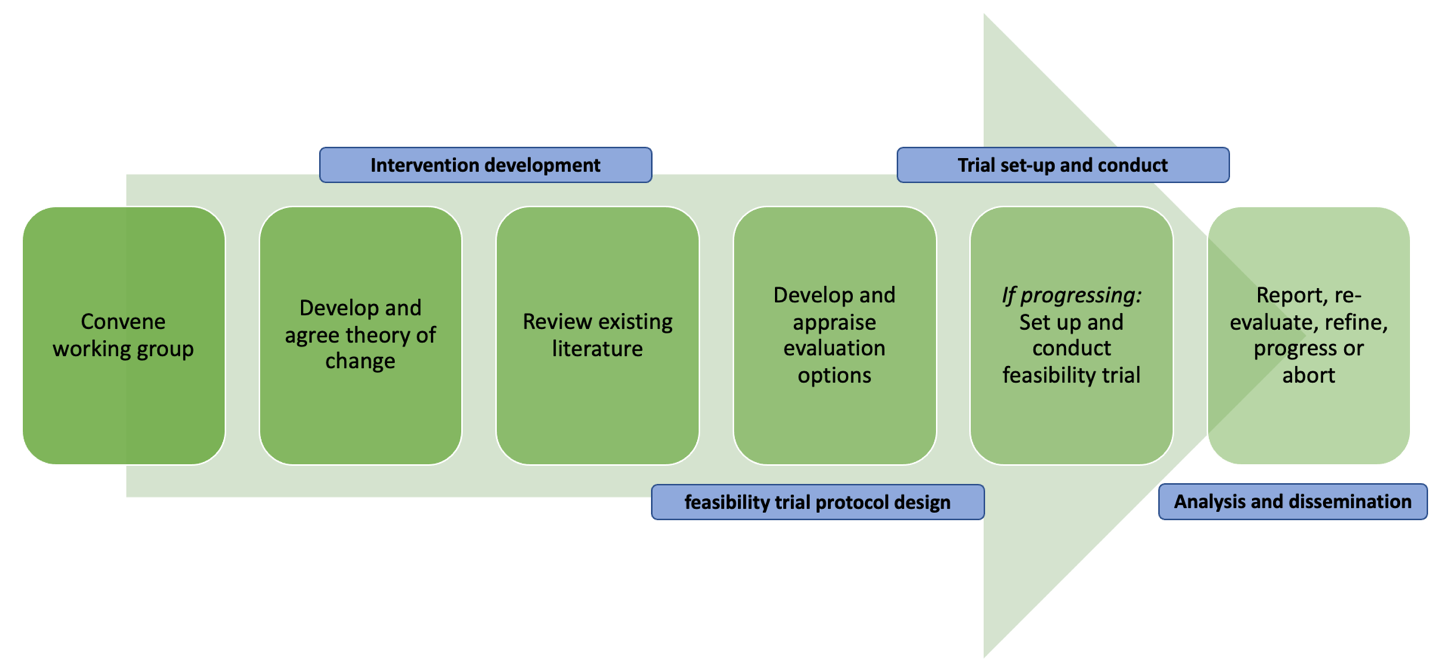


**Figure 2: Process flowchart of intervention development and evaluation**. Adapted from: Craig P, Campbell M. Evaluability Assessment: a systematic approach to deciding whether and how to evaluate programmes and policies: a What Works Scotland Working paper. 2015.

### Programme flexibility and specificity

When trying to understanding *NeuOst* as a complex intervention according to the MRC framework, two further topics need to be discussed: programme flexibility, and a consideration of specific versus contextual components (Skivington et al., 2021).

“*Flexibility in intervention delivery and adherence might be permitted to allow for variation in how, where, and by whom interventions are delivered and received*.” (Skivington et al., 2021, p. 2). At the early stage of intervention development, the “Where” and “by Whom” will remain well-defined, with *NeuOst* delivered in the setting of an osteopathic educational institution (University College of Osteopathy, London) and by fully qualified and specifically-trained osteopaths. The latter will remain the providers, even though adaptations to other manual therapy professions may be feasible. Regarding “How” the intervention is applied, the initial programme theory allows for intervention components to be chosen and adapted depending on patient needs and preferences, and the clinical decision-making of providers. Boundaries are, however, defined below.

During a feasibility trial, and especially a later efficacy trial, intervention flexibility may have to be reduced and patient adherence enhanced for reasons of trial interpretability and bias reduction. At the later stage of pragmatic effectiveness research, such restrictions may be less desirable and can be replaced by the measurement and appraisal of heterogeneity instead (Hohenschurz-Schmidt et al., IMMPACT, almost submitted).

**Table *1*** and **Figure 4** identify the core components of NeuOst and hypothesised or known mechanisms of change. For an adequate understanding of complex interventions and for the subsequent efficacy studies, however, contextual factors need to be identified, too. Current research, however, holds that contextual and specific components are mutually intertwined and likely influence treatment effects in a non-additive non-linear manner (Hall and Loscalzo, 2019). Moving away from the specific/non-specific dichotomy, current consensus on the design, implementation, and reporting of sham controls in trials of physical, psychological, and self-management therapies for pain requires researchers to instead identify the treatment components whose effects they want to study in a trial (Hohenschurz-Schmidt et al., under review). This decision is therefore relegated to the stage of trial development, not intervention development, and will be considered in the respective feasibility trial protocol and sham control development.

### Intervention refinement and boundaries

Advocating continued refinement as complex intervention development moves from one stage to the next, the MRC framework advocates for refinements to be informed by intervention users. To do so, we will gather such information at at two initial stages: During the initial development of the programme theory, using PPI as described above. Secondly, by using quantitative and qualitative methods during a subsequent feasibility evaluation. If the *NeuOst* intervention progresses beyond these stages, PPI will continue to guide refinements.

At the same time, the MRC framework states that “*refinements should be guided by the programme theory, with acceptable boundaries agreed and specified at the beginning of each research phase, and with transparent reporting of the rationale for change*” (Skivington et al., 2021, p. 5). Whilst the initial *NeuOst* programme theory is being developed, such boundaries will be explored and negotiated. For now, potential boundaries include:

- Medico-legal boundaries: Defined by the General Osteopathic Council, the Osteopathic Practice Standards (OPS) set out the professional standards expected from osteopaths (General Osteopathic Council, 2018) to which providers of the *NeuOst* intervention will have to adhere. These standards are grouped in “Communication and patient partnership”, “Knowledge, skills and performance”, “Safety and quality in practice”, and “Professionalism”. Whilst many of these standards are generic, knowledge- and -safety-related themes may require *NeuOst* to ensure adequate diabetes-specific training for providers.
- Medical boundaries: Relatedly, non-pain-related complications of diabetes need to be detected and appropriately referred for, and not treated by *NeuOst* providers. Similarly, referral for psychiatric illness may be required. The aim of *NeuOst* is not for osteopaths to become Jacks of all trades, but to expand the scope of practice by working with people with chronic disease and often multiple comorbidities, whilst maintaining a core set of skills in the realm of musculoskeletal health and pain management.
- Content boundaries: As per **Table *1***, common elements of osteopathic manual therapy practice form a non-negotiable core of the intervention. During the development process, other core elements will be agreed upon, which may include components / techniques informed by
- CBT-type interventions, provision of certain physical or mind-body exercises, etc. With all these added components, training limits may have to be agreed, as the aim is again not to become osteopaths fully trained in psychotherapy or nutritional therapy, but to find ways of augmenting the skillset in a realistic and applicable manner. However, which techniques are ‘borrowed’ from other professions and how providers may be trained in them, may change over time.
- Intervention intensity boundaries: It will have to be evaluated whether *NeuOst* is best provided as a relatively short-term but high-intensity programme or as “maintenance care”, a common treatment model in manual therapy practice (Eklund et al., 2018) and potentially suitable to populations with chronic underlying disease.

### Stakeholders

The main stakeholders of *NeuOst* are people with pDPN and osteopaths. Those affected by pDPN will be the participants of the novel programme which is designed to positively influence these people’s lives. Therefore, people with a lived experience will be involved in the intervention development, trial planning, study oversight, and in the interpretation and dissemination of findings. The aims being pursued are to:

- Ensure relevance of the intervention objectives to the needs and requirements of people with pDPN
- Promote the acceptability of the proposed intervention and of procedures of the feasibility trial
- Improve programme evaluation methods
- Facilitate participant recruitment and retention for a feasibility trial through intervention development and trial planning with close Patient and Public Involvement (PPI)
- Enhance sham comparator acceptability and blinding effectiveness by mean of involving patients in pre-trial development
- Enhance uptake of findings in the patient community

Secondly, the intervention programme will be delivered by specifically trained osteopathic practitioners, building on their existing practice, and possibly integrated into their private practice at a later stage. This requires involvement of practicing osteopaths to assure compliance with practical requirements and preferences. Further, the mechanistic rationale of manual therapies for DPN symptoms is poorly developed in the literature. It is therefore necessary to draw on practical experience of providers working with people with diabetes, to make decisions about intervention content and possibly develop a testable mechanistic hypothesis. Specifically, the aims of involving osteopaths in the project are to:

- Learn from osteopaths who have cared for people with (painful) neuropathies over longer periods of time and in various settings (may include preferred treatment models, manual techniques, and patient management approaches)
- Improve feasibility of intervention delivery from a provider perspective (including sham intervention in feasibility study)
- Where required, ensure alignment of *NeuOst* with the practice, preferences, values, and philosophies of practicing osteopaths
- Where *NeuOst* broadens the scope of osteopathic practice, encourage the positive uptake of novel intervention principles in the osteopathic community

Methodologically, members of the public will be recruited for individual PPI interviews or sustained accompaniment of the study, as per their preference. Depending on public interest, a group of 3-6 core stakeholders will be formed.

PPI meetings will be held at least every six weeks at all stages of the project, usually separating patient and practitioner meetings. One or more people with a lived experience of pDPN will be recruited to an independent trial steering group, monitoring the feasibility trial. Recruitment will be through the Diabetes UK charity, social media posts, and existing PPI networks of collaborators.

Whilst aims and methods of stakeholder involvement were reported here, the final study report will include the presentation of results, reflections, and critical appraisal of PPI activities in this study, following the Guidance for Reporting Involvement of Patients and the Public (GRIPP2) (Staniszewska et al., 2017).

### Key uncertainties and research perspectives

Little is known about the potential role of manual therapy in the care for people with peripheral neuropathy, neuropathic pain, and chronic metabolic disease such as diabetes. Therefore, the first step is to determine the feasibility of integrating additional relevant treatment components into a manual therapy core. Acknowledging the complexity of the proposed intervention, refinements and several development rounds may be required. Here, a mixed method evaluation and parallel process evaluation will be crucial to identify challenges and guide refinement (Skivington et al., 2021).

Having promoted the feasible delivery and acceptability of *NeuOst* through extensive development, PPI, and pre-trial testing, the second key uncertainty involves acceptability of *NeuOst* to patients and providers. This will be studied in a feasibility trial with pre-defined and stakeholder-informed feasibility criteria.

At the same time, testability of the *NeuOst* programme in an efficacy trial needs to be ascertained. Of course, this is linked to the overarching uncertainty of intervention efficacy, manual therapy not having been researched much in pDPN. One of the main challenges in the trial design is the design and implementation of a high-quality control intervention, designed to test the clinical efficacy of certain treatment components. Whether *NeuOst* can be tested in a parallel-group randomised sham-controlled clinical trial will be studied in a feasibility trial with 24 participants, once the initial intervention development is completed.

In line with the MRC framework’s updated comments on research perspectives, questions of implementation, effectiveness, cost-effectiveness, translation to other settings, and scalability are to be addressed if acceptability is ascertained (Skivington et al., 2021) and once signals of potential efficacy are obtained (Freedland et al., 2019).

# Intervention development

## Pathophysiological and clinical changes in painful DPN and target mechanisms of NeuOst

### Neurological mechanisms

DPN can present with gain-of-function or loss-of-function, where patients experience hyperalgesia, allodynia, spontaneous pain, paraesthesia and feelings of heat, or numbness, weakness, and loss of deep tendon reflexes, respectively. As discussed before, about a quarter of people with DPN experience neuropathic pain, often described as burning, shooting, or shock-like. This highly debilitating experience is caused by a combination of abnormalities of nerve conduction (due to combination of axonal degeneration and demyelination), aberrant neural firing, and central neurological mechanisms (Chao et al., 2021). DPN is a peripheral polyneuropathy, characterised by symmetrical progressive length-dependent (i.e., longer nerves affected before shorter ones) nerve compromise. This can affect all fibre types, but their involvement may vary and lead to different phenotypes: Small-fibre dominant neuropathy involves more compromise in pain, temperature sense, and autonomic function, whilst large fibre lesions cause disturbances of sensitivity to light touch, pressure and vibration, and reduced joint position sense. Advanced cases can experience distal muscle weakness and wasting (Said, 2007).

The mechanisms of neuropathic pain in DPN are not entirely understood. Comparing people with painful and painless DPN, neuropathic pain is more prevalent (and likely more severe) in people with less diabetic control (Themistocleous et al., 2016).

The most commonly employed biomarkers to study nerve function in this population are nerve conduction studies (NCS) and vibration perception threshold (VPT) but others have been used, including quantitative sensory testing (QST) (Themistocleous et al., 2016) and structural magnetic resonance imaging (MRI) (Evans et al., 2021). Here, nerve conduction studies do not discriminate between people with or without NeuP and DPN (which is likely unsurprising considering nerve conduction studies are only sensitive to large, not small fibre function), and intraepidermal nerve fibre density is also not different (Themistocleous et al., 2016). On QST testing, many patients with painful DPN have hyposensitivity across a range of small- and large-fibre mediated sensory modalities, with sensory gain signs being rare. Clinical questionnaires for the detection of NeuP are the NeuPSIG screening tool, the DN4, and painDETECT, with the DN4 showing the highest sensitivity and specificity (Themistocleous et al., 2016).

The primary therapeutic objectives with regards to length-dependent DPN are the halting or slowing down of disease progression, the minimization of complication risk (Said, 2007), and symptom management (Gilron et al., 2015).

Treatment mainstays are diabetes control (through exercise, diet, and pharmacological treatment) and drugs for neuropathic pain (Finnerup et al., 2015). There is almost no research on non-pharmacological therapies to modulate disease parameters of symmetrical neuropathies such as nerve conduction (Liampas et al., 2020; Moisset et al., 2020; Hernández-Secorún et al., 2021); Any existing evidence (mainly from traditional Chinese therapies) is of low to moderate quality (Fu et al., 2020; Lin et al., 2021).

### Musculoskeletal mechanisms

Rheumatological diseases are commonly associated with DM, driven mainly by the harmful effects of advanced glycation end products on connective tissues (Abate et al., 2013). Pathophysiological changes of muscle and bone are also common but may present more subtly clinically (Sözen et al., 2018). These are due to metabolic effects of excess insulin, blood sugars, and inflammation as well the effects of neuropathy. In fact, most musculoskeletal complications are more common in people with diabetes *and* neuropathy than those without.

Clinically, fibroproliferative disorders include adhesive capsulitis of the shoulder, carpal tunnel syndrome, limited joint mobility syndrome as well as ‘trigger finger’ and Dupuytren contractures (Abate et al., 2013). There may also thickening of the Achilles tendon and plantar fascia, likely contributing to changes in gait mechanics (reduced ankle mobility) and foot complications. As such, Charcot (osteo)arthropathy of the tarsal and tarso-metatarsal complex is a major long-term complication of DM and, together with foot ulcers, contributes to disability and an increased amputation risk (Sözen et al., 2018). Patients may experience muscle weakness, especially of distal muscles and progressing proximally (Andersen, 2012; Almurdhi et al., 2016; Alam et al., 2017; Parasoglou et al., 2017). Balance problems are also commonly reported (Khan and Andersen, 2022). The combination of altered balance, gait mechanics, and osteoporosis leads to an increased risk of falls in DM (Khan and Andersen, 2022), and hip fractures being 6-9 times more prevalent in people with T1DM compared to healthy matched controls (Sözen et al., 2018). Unsurprisingly, older adults with DM have low balance confidence and high fear of falling (Hewston and Deshpande, 2018).

Consequently, evidence-informed therapeutic objectives for non-pharmacological programmes such as NeuOst are as follows:

- enhance (ankle) mobility
- build strength / muscle, or slow down deterioration
- promote postural balance
- improve glycaemic control
- address fear of falling
- screen for complications early (adh. capsulitis, risk of falling, ulcers etc)

Overall, successful interventions with these objectives should reduce complication risks. Together with other elements of the intervention, the more immediate objectives would be to improve measures of function, disability, and quality of life.

### Psychological, social, and behavioural mechanisms

Compared to people with DPN and no pain, people with moderate to severe NeuP and DPN experience significantly more physical disability, pain-related worrying, depression, anxiety, insomnia, and poorer quality of life (SF-36) (Themistocleous et al., 2016; Kioskli et al., 2019a).

In people with painful DPN, Kioskli et al. (2019b) studied whether the concept of psychological flexibility might be relevant to people’s pain levels, emotional experiences, and daily activities. Psychological flexibility is a fundamental concept of Acceptance and Commitment Therapy (ACT), comprising of six psychological skills, illustrated in **Figure 3** (McCracken and Morley, 2014; Hart and Beckley, 2015; McCracken et al., 2022).


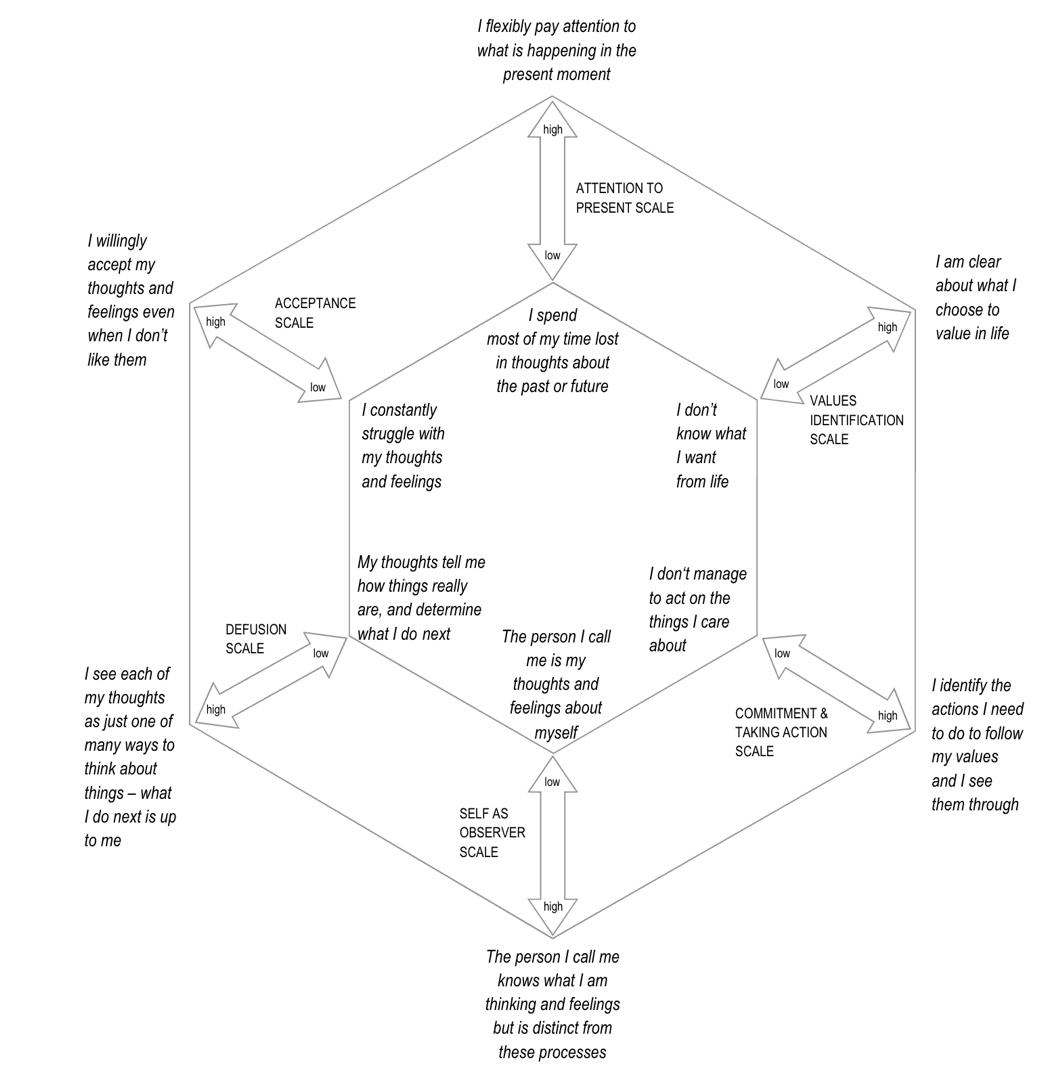


**Figure 3: The Psychological Flexibility Model.** From McCracken, L.M., Morley, S., 2014. The Psychological Flexibility Model: A Basis for Integration and Progress in Psychological Approaches to Chronic Pain Management. The Journal of Pain 15, 221–234. <https://doi.org/10.1016/j.jpain.2013.10.014>

ACT targets psychological flexibility to improve patients’ functioning, and an interdisciplinary ACT-based rehabilitation programme has been shown to beneficially influence these processes in people with persistent pain (Scott et al., 2016). A comparable programme has been developed for online-use for people with HIV-related neuropathic pain and was shown to be acceptable (Scott et al., 2020). Kioskli et al. (2019b) argue that pain severity is associated with psychological functioning for people with pDPN, and that including psychological flexibility components may improve psychological treatments for people with DPN. Treatment adherence during an online feasibility trial with people with pDPN was, however, low (Kioskli et al., 2020).

ACT-informed manual (Carnes et al., 2017) and physical therapy (Godfrey et al., 2016, 2020) have shown to be promising. However, challenges to provider training and high-fidelity implementation are occasionally highlighted (Keefe et al., 2018). However, a trial studying the effects of a physiotherapist-delivered course of ACT-informed physical therapy (3 sessions) showed small effects on pain and disability at three months compared to usual care, and achieved high intervention fidelity (Godfrey et al., 2020). ACT-therapy-derived measures such as acceptance and committed action were, however, not altered by the intervention. Physical therapists believed that the taught skills were fully within their scope of practice, whilst the study authors stress that their programme was “*not designed to turn physical therapists into ACT practitioners*”. Providers were trained in ACT principles during a 2-day face-to-face programme, monthly group supervision, and had a patient manual available.

In the context of NeuOst, a complex or high-fidelity ACT-based component is unlikely to be successfully taught to providers; NeuOst being a multimodal intervention with many other components and limited training period. Nonetheless, it appears feasible to include the following ACT-derived or -related features into the provider training programme:

Through pre-recorded provider training videos:

- Psychosocial implications of living with persistent (neuropathic) pain
- ACT principles
- Mindfulness principles
- Reflective and mindfulness exercises for providers

In face-to-face training:

- Application of ACT-informed communication techniques during roleplay

In regular online meetings:

- Supervision from experienced practitioner

## Intervention theory of NeuOst: context and components

In the following, the context, programme theory, stakeholders, key uncertainties, and plans for intervention refinement of NeuOst will be described.

### Context definition

The socio-political context of NeuOst is given by the situation of people with diabetes, and more specifically pDPN, in the UK. As a disease with strong links to socioeconomic determinants of health, type 2 diabetes has become more prevalent in the UK in the past 12 years of austerity policies (Whicher et al., 2020; Marmot, 2020). Economically, at least 10% of the National Health Service’s (NHS) budget (£10 billion) is spent on diabetes, 80% of which on disease complications.

With multiple complications, including foot ulcerations, blindness, mental health problems, and neuropathic pain, diabetes causes personal suffering and has a large impact on families, carers, and society (Whicher et al., 2020).

In the NHS, diabetes patients are managed by their General Practitioners or referred to specialist services. Whilst management of type 1 diabetes relies on education, blood glucose management, physical activity, and insulin therapy, the treatment of type 2 diabetes can involve medications such as metformin. In the NHS, non-pharmacological management of type 2 diabetes focuses on dietary interventions for weight and blood glucose management, structured education, and screening for and management of disease complications (McGuire et al., 2016). The uptake of many educational and behavioural interventions is, however, poor. Importantly, accessibility of key NHS services is also suboptimal (Whicher et al., 2020).

Amongst the sequelae of diabetes is neuropathic pain (pDPN). Depression, anxiety, low quality of life, and poor sleep are associated with pain in pDPN (Kioskli et al., 2019a). Healthcare options for people with pDPN are largely limited to pharmacological therapy, notably amitriptyline, duloxetine, gabapentin or pregabalin (Tan et al., 2010). These drugs have only moderate effectiveness and can produce side effects such as drowsiness and dizziness (Finnerup et al., 2015), likely reducing their acceptability amongst patients. Non-pharmacological pain management is only provided in specialist NHS pain services, notably by psychologists, physiotherapists and specialist nurses. However, these services may not be accessible to people with pDPN because other patients are usually classed a higher priority (Annina Schmid, personal correspondence). Because of the low average income of people with (type 2) diabetes (Espelt et al., 2011), access to private services is likely limited.

Osteopaths are Allied Healthcare Professionals, trained in medical differential diagnosis, manual therapy techniques, and the conservative biopsychosocial management of people experiencing pain (Institute of Osteopathy, n.d.). This expertise, however, mainly pertains to people with musculoskeletal conditions and non-specific pain, most of whom are treated in private practice (Plunkett et al., 2021). In the UK, osteopathy is a profession regulated by the General Osteopathic Council which also defines practice standards, including the need for osteopaths to recognise whether they have the training, skills and competence to treat a particular patient (General Osteopathic Council, 2018). Taken together, the current focus of osteopathic training on musculoskeletal health and the predominance of private practice models means two things: First, additional postgraduate training may be required to enable osteopaths to safely and competently work with people with diabetes and neuropathic pain. Secondly, low-threshold access to care may require subsidised private practice models, the help of educational institutions (some of which have existing community clinics providing subsidised care), or, eventually integration into NHS pathways.

Within the above-described context, NeuOst is to be provided by specifically trained osteopaths to people with diabetes and neuropathic pain, likely in private practice or teaching institutions and over the course of multiple treatment sessions.

NeuOst can also be illustrated with a logic model, visualising the proposed theory of change (

**Figure *4***). Importantly, the MRC framework also asks how complex interventions may be influenced by, and may themselves influence, their context. Ideally, NeuOst is hoped to alter the relationship of people with pDPN with their social and physical environment, by promoting interaction and initiative. Within the osteopathic community, NeuOst is hoped to stimulate discussion about the role of osteopaths in a post-reductionist paradigm of care, in the wider UK healthcare system but also in society at large. Clearly, the socio-political context of many people with diabetes in the UK imposes limitations on their ability to engage in treatment programmes and is not conducive to the prevention of type 2 diabetes in the first place. Therefore, questions of real-world effectiveness and generalizability to non-trail populations will have to be considered carefully at later stages of the intervention development; Conversely, interaction with this group of people may encourage osteopaths to take on advocacy roles at various levels.


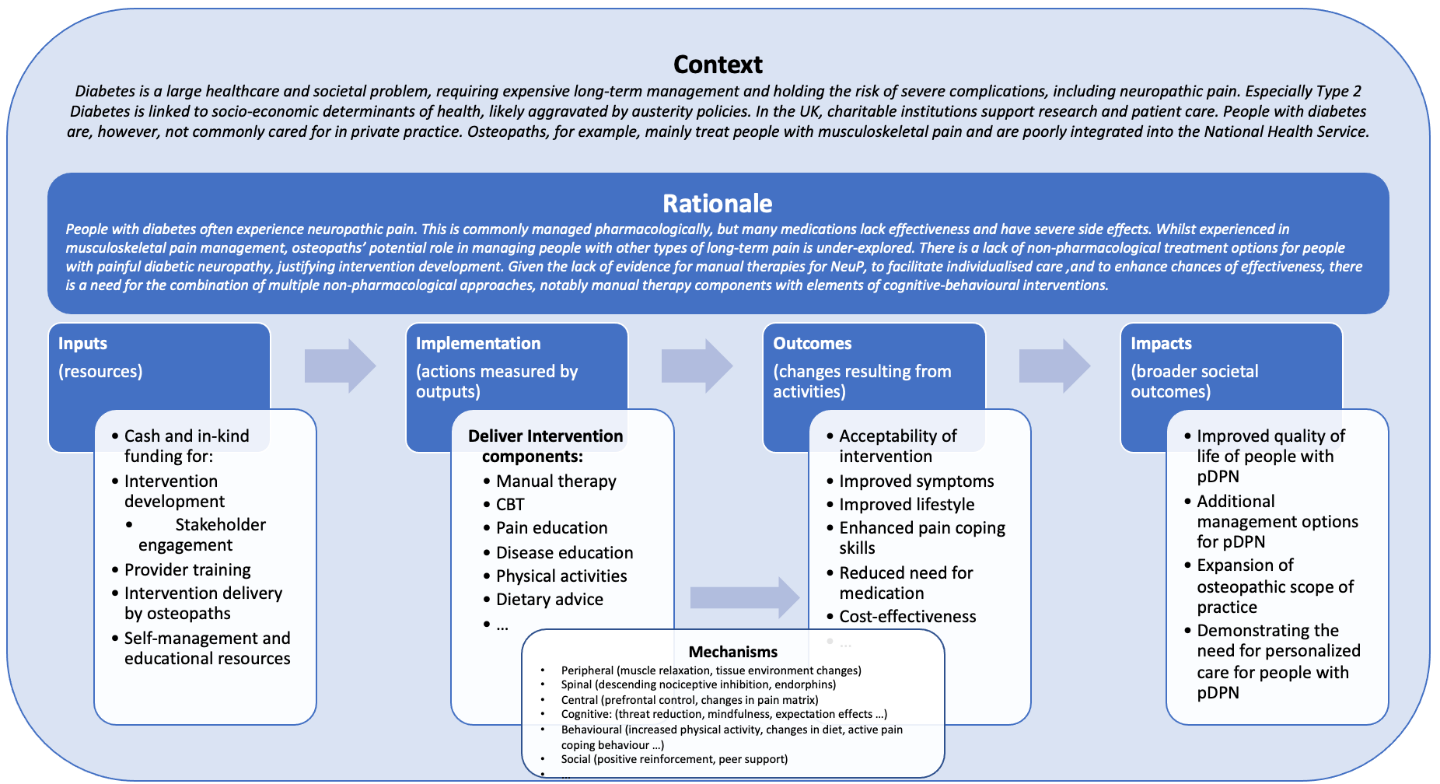


**Figure 4: Logic model of NeuOst complex intervention.** Created based on: https://www.gov.uk/guidance/evaluation-in-health-and-wellbeing-creating-a-logic-model & https://www.strategyunitwm.nhs.uk/sites/default/files/2018-03/Logic%20models%20and%20complex%20programmes%20-%20a%20brief%20guide.pdf

### Programme theory

Programme theory describes the key components of an intervention, how an intervention is expected to have effects, as well as how intervention components are expected to interact with one another and with the intervention context (Skivington et al., 2021) (Figure 4).

The NeuOst intervention has several basic components, defined by current osteopathic practice and curricula, and informed by engagement of practicing osteopaths. Added components were chosen to ‘augment’ osteopathic practice with compatible techniques thought to be effective for people with pDPN (See table 1), based on involvement of subject experts, providers, and people with painful DPN in the intervention development. The basic osteopathic components include individualised biopsychosocial case history taking, manual therapy techniques for diagnosis (palpation, inspection, functional testing) and intervention (soft tissue techniques, joint and spinal articulation and manipulation), and some behavioural or pain management advice, and often anatomical education (Fawkes et al., 2009). Additional components include physical therapy and rehabilitation elements, educational components from diabetes care, and psychologically-informed techniques.

The below table describes the content of individual components of the multimodal NeuOst programme. The table also provides a description of how these components are thought to influence outcomes (mechanism) and interact with other components. It further lists existing evidence of components’ efficacy / effectiveness. Importantly, the table begins to outline how individual components may be integrated into the multimodal programme. This includes considerations of how each component may be “triggered” by individual patient needs (vs. being a standard component of the programme), and processes of adapting components to patient needs (individualisation) versus needs for standardisation. During intervention development, the below table formed the main tool for subject experts to comment on programme components, and for patient and provider stakeholder preferences to be incorporated. In doing so, programme theory “*promotes a shared understanding of the intervention among diverse stakeholders*” (Skivington et al., 2021. p. 4). Once a theory of change had been agreed, the table formed the basis of a programme flowchart and decision-making algorithm, as well as the provider training that conveys NeuOst content to practicing osteopaths. In the subsequent stage, options for the evaluation of NeuOst were developed (feasibility trial protocol design) (Craig and Campbell, 2015).

### Patient and public involvement

The main stakeholders involved in the development of NeuOst were people with pDPN and osteopaths or physiotherapists with experience in working with people with polyneuropathies.

Five people with pDPN were interviewed online, one of them twice. This last person also attended an in-person meeting where the first NeuOst session was tested, including the manual therapy components. All but one patient participants were T2DM with varying degrees of neuropathy (in some cases undiagnosed and even questionable), one person had chemotherapy-induced neuropathy, there were no amputations, the mean age was 74 years, and there were three women and two men (with an further male person participating in a practical development session, detailed in Appendix 2).

Further, three osteopaths and a physiotherapist were interviewed. Two of the osteopaths have worked as clinic tutors in a community clinic for people with HIV for many years; The third osteopath had been treating their partner for chemotherapy-induced neuropathy (interviewed as a couple) and had thus become interested in the treatment of people with neuropathies. The physiotherapist led the physiotherapy component of a regional outpatient pain management programme in Germany. All providers had been practicing for over 20 years.

Patient interviews highlighted the following topics:

1. Diagnostic uncertainty
   1. Not all patients were diagnosed with neuropathy and solely contacted the study team based on the call for people “with diabetes and pain in the legs”.
2. Knowledge variability
   1. Whilst some were aware of neuropathy and its basic mechanism, other had hardly ever heard of the role of their nerves and were glad to be explained some of the principles. Similarly, the link between neuropathy and Charot foot or ulcerations was not apparent to all of them. Some patients also complained that nobody had taken the time to educate them about the basics of diabetes complications, including neuropathy and foot care.
3. Impact of symptoms
   1. Most patients reported considerably reduced quality of life due to the neuropathy symptoms. Pains at night and interference with sleep seemed the most troubling. A single patient suffered from recurrent ulcerations but only had temporary neuropathic pain; Here, the patient was most preoccupied with foot care and the avoidance of wounds. Finally, balance and the fear of falling figured strongly in those with also otherwise more debilitating experiences.
4. Diabetes management
   1. DPN cannot be considered independently from living with diabetes. This was most starkly apparent from a somewhat inconclusive call that the researcher first out down to the old age of the participant, before the person contacted the researcher a few hours later, apologising that they had become “rather low” (in blood glucose) and could not remember much if the conversation. They hoped to “have made any sense”. At this point, the researcher became aware that only repeated work with people with diabetes and / or careful training would make providers to be sensitive to such possibilities and prepare them to provide first-aid hypoglycaemic treatment.
5. Desperation
   1. Most patients were desperate for non-drug treatment options for their pain. Those who had tried available medication disliked it for its side effects and did not take it regularly. Others were trying various alternative creams and ointments.
6. Different attitudes towards exercise
   1. Patients felt overall positive towards physical activity but engaged in it to very variable degrees and felt differentially confident about performing different activities. Again, the fear of falling and of wounds were strong for a person with respective balance problems and previous experiences. This person nonetheless engaged in daily dance exercises at home, broken up into several chunks a day, and advocated for safeguards such as opportunities to hold on to something whilst performing the exercises and to adapt the degree of challenge for balancing exercises. Others considered themselves active but mainly mentioned continued management of their chores at home or regular walks, not really limited by their neuropathy symptoms.
7. Positive attitude to manual therapy
   1. Although only one patient (the partner of an osteopath) had had manual therapy for their symptoms, all were very positive about the possibility and were willing to try it in theory. Nobody voiced concerns regarding safety or practicability. There was a sense of hope at the possibility (likely following from the feeling of desperation described above). The person with chemotherapy-induced neuropathy described improved sleep after foot mobilisations and massage in the evenings, but only for the following night so that their partner was treating their feet most evenings. The person treated by the researcher provided the following written feedback one week later:

“*No adverse effects as in pain. My legs felt great no sign of tiredness until the next day - then I became aware how tired my legs can get. The foot exercises are good. They help release a ‘floppy’ feeling / not having to unknowingly clench muscles in your feet and legs while you are waiting for the inevitable pain to come. So, there is already a chance that if neuropathy pain comes on it may not be as bad as you normally get as your limbs were more relaxed. It would be a great feeling for persons with pain as it was a relief you did not know you needed. Something different to take your mind off the one constant annoyance you endure. As I walked the final part after having taken the bus home I thought ‘yes I needed to go for massage on my shoulders and neck’!*

*That would have completed the experience. I enjoyed the session completely.*”

Provider interviews led to the following insights:

1. Treatment approach and rationale
   1. People did not have an elaborate reasoning behind the application of their techniques other than to “move the feet” and “provide sensory input”. They mainly advocated for a prolonged mobilisation of the feet to “stimulate the Golgi tendon organs” as well as generic articulatory and soft tissue treatment of the lower extremities and potentially lumbar spine.
2. Dampened expectations
   1. Providers did not perceive the manual component as life-altering for patients, but believed they provided short-term relief. They related patient stories where even a few hours relief were deemed worthwhile by patients, but highlighted that this meant regular (e.g., weekly) treatment that was unlikely to be possible in a private practice setting.
3. Effects beyond the musculoskeletal domain
   1. Practitioners highlighted areas of treatment that were often as important to them as the physical side. For one person (the physiotherapist), this was behaviour change and changing unhelpful beliefs around the pain experience. For the osteopaths that had worked in community clinics this meant social support and the ability to manage disease complications (mainly of HIV), for example by integrating with medical care or by signposting accordingly.

### Intervention components table

The below table provides an overview of potential intervention components, amenable to osteopathic practice, and to be combined in *NeuOst*. At the development stage, this table served as the basis for discussion amongst the research team and during stakeholder engagements.

Intervention elements can be split into manual and non-manual groups and pre-defined ‘techniques’ in each group (following the UK Back pain Exercise And Manipulation (UK BEAM) trial approach (Harvey et al., 2003)). During the feasibility trial, practitioners will be expected to deliver certain components in each, with some flexibility as to specific techniques and their mode of delivery, and are not allowed to deliver treatment components not specified in the protocol. ACT-informed techniques were based on the unpublished ‘ACT OPEN Workbook’, used to accompany online ACT treatment delivered for people with HIV-related neuropathy (Scott et al., 2021) and similar to the workbook used in Kioskli et al.'s (2020) study with people with pDPN.

**Table 1: Intervention components of NeuOst.** Manual therapy components and techniques based on Harvey et al. (2003) classification, with ‘subtle’ manual therapy components added.

| **Intervention Type** | **Intervention Components** | **Treatment targets / Mechanisms relevant to pDPN** | **Evidence of Efficacy and / or Effectiveness** *(population, study design, comparator, n total, main findings)* | **Potential role in Multimodal Intervention** | **Expected interaction with other components** |
| --- | --- | --- | --- | --- | --- |
| **Manual therapy-based components** | | | | | |
| **Soft tissue components** | Massage of painful and non-symptomatic areas; deep vs superficial; painful vs pleasant techniques  (Techniques: cross-fibre stretch, longitudinal stretch, direct pressure, deep friction) | counter-pain mechanisms; overall / ANS relaxation; muscle relaxation; expectation effects | A review that included (six) manual therapy trials found Thai foot massage, Gua Sha, reflexology and aromatherapy to be more effective on pain and function parameters than education controls, TAU, or no treatment. Also included trials on CTS in people with DM (Hernández-Secorún et al., 2021). Largest trial apparently Chatchawan et al. (2015) on six sessions of Thai foot massage vs educational control (total n = 60), finding benefits in functional and sensation tests.  Looking at primary pain-outcomes and >3-week follow-up in neuropathies, Moisset et al. (2020) identified no eligible studies from most non-drug fields, including physiotherapy, therapeutic patient education, Tai Chi or Chinese acupuncture.  For spinal manipulation for peripheral (poly-)neuropathies, no human studies could be found. (See Song et al., (2016); Onifer et al., (2018) for rodent studies)  Here you can cite the literature on immediate neurophys effects of spinal mobilisation, see also our (now rather old Systematic review in manual therapy 2008). Mostly short term effects though, but pain modulatory plus sympathetic NS. | List of eligible techniques that can be applied freely depending on provider choice and patient preferences. | Forming the basis of ‘treatment’ during in-person sessions; Hoped to facilitate engagement in more active self-management components through short-term relief.  Arguably enhancing the therapeutic relationship / alliance through touch (McGuirk, 2012; Kerr et al., 2019). |
| **Articulatory / mobilisation components** | (Techniques: low- through high-amplitude passive movements of lumbar spine and sacroiliac joints (and necessarily hips); flexion, extension, rotation, side-bending, manual traction; oscillation) |  |  |  |  |
| **‘Subtle’ Manual Therapy components** | (Myofascial, craniosacral, and visceral techniques)  Gentle hands-on approaches, usually based on structural models of health and disease or palpation of tissue tension. | ANS relaxation; muscle relaxation; expectation effects |  |  |  |
| **Thrust components (‘manipulations’)** | (Techniques: high or low velocity; low amplitude; direct or leverage; directed at central lumbar, zygapophysial or sacroiliac joints; unilateral or bilateral; at one or more locations)  Thrust force; Cavitation; joint loading and tension; sensory experience | muscle relaxation; descending pain inhibition; temporarily enhanced joint mobility; expectation effects |  |  |  |
| **Soft tissue component 2 (Neurodynamic techniques)** | Manual techniques that aim at mobilising a nerve in its peripheral course. Can also be provided as home-programme. | Usually applied to suspected entrapment neuropathies. Role in pDPN to be explored. Techniques for sciatic and distal nerves likely most relevant. | Single trial on “nerve mobilisation” (Singh et al., 2012): n = 30, DM2, 21 days tibial nerve stretch (Dorsiflexion + Eversion + SLR, 10 mins per day) compared to no treatment. Found sign. b/w group differences in changes in vibration thresholds. High RoB and 4/11 PEDro score according to (Hernández-Secorún et al., 2021).  In chemotherapy-induced neuropathic pain, a recent exploratory RCT suggests usefulness of upper extremity home-based programme (daily 5-10 minutes starting prior to chemotherapy) (Andersen Hammond et al., 2020) | Introduced during in-person treatment sessions and provided as part of home-exercise programme | Potentially reinforcing benefits of manual therapy; Linking in with activity-increasing aspect of home-exercise programme. |
| **Non-manual therapy-based components** | | | | | |
| **Physical activity / Exercise components** | | | | | |
| **Exercise, Sensorimotor / balance advice and prescription** | Advice and provision of exercise plans, including detail on form, sets, and repetitions, frequency, and individualisation.  Type of exercises:  **Sensorimotor / balance training**; regimen provided as training plan (see Appendix in (Ahmad et al., 2020)) | Sensorimotor / balance training:  Supposed altered motor programming and movement patterning through repeated proprioceptive and somatosensory input (Ahmad et al., 2020). Fall prevention and avoidance of pressure areas on feet (risk reduction for ulcerations) (Hernández-Secorún et al., 2021). | Sensorimotor / balance training: Hernández-Secorún et al. (2021) reviewed 12 moderate-to-high-quality trials of various balance programmes in DPN, one with vibration elements. They report largely significant benefits on various proprioceptive, some neurophysiological and EMG, and few functional or disability outcomes, compared to attention or TAU controls. E.g., some indication from small attention-controlled RCT (n = 38) that proprioception, EMG, and potentially nerve conduction velocity may be improved (Ahmad et al., 2020). Ankle-reaching task + virtual obstacle crossing (2x per week, 4 weeks; n = 39, elderly) showed some effects on balance over no-treatment control (Grewal et al., 2015). Note on **dosage**: All studies have session schedules of at least 40-60mins once per week, usually more frequent. | Could be either provided to those people who express an interest in a home-based exercise plan, or as generic take-home programme. Equipment would have to be provided or improvised or exercises adapted; Initial practice within clinic session; Safety concerns? | May interact with below advice on general activity, for example by setting the basis for starting some form of physical activity or allowing for movement even during very painful episodes, or by improving proprioception and making daily activities easier. Also interact with mindfulness and pain education components, trying to reframe movement as beneficial and non-threatening. Interaction with manual components: providing additional sensory input. |
| **Exercise (Physical, Stretching)** | Static and / or dynamic lengthening of muscle groups of varying duration and sequences. |  | No studies found that examined solely stretch exercises, but stretches were included in most regimes reviewed by (Hernández-Secorún et al., 2021) | Can be provided at very low intensity and may thus be suitable for people with high levels of pain or high kinesiophobia as introductory exercises. Practiced during in-person sessions and provided as “homework”. | May be facilitated through short-term relief from manual therapy and enabled through cognitive changes from CBT-based and pain education components (incl. goal setting techniques). Could be combined with elements of mindfulness or controlled breathing. |
| **Exercise (Physical, anaerobic / Strength)** | Strengthening exercises with body-weight, resistance bands, or weights. Variable repetitions; load parameters; addressed muscle groups; May include social components. | Autonomic; muscle strength; coordination; proprioception; endorphins | Reviewing 4 trials in DPN, all of which deemed strength training effective in balance and strength variables compared with no-treatment control groups (Hernández-Secorún et al., 2021). | Provided depending on patient preference and disability levels; Potentially practiced during in-person sessions and provided as “homework”; | May be facilitated through short-term relief from manual therapy and enabled through cognitive changes from CBT-based and pain education components (incl. goal setting techniques). |
| **Exercise (Physical, aerobic)** | Cardiovascular exercises from fast walking to running / cycling / swimming. Variable in duration; intensity; social; whole-body vs legs only | Cardiovascular and autonomic; proprioception; endorphins; (Diabetes-related mechanisms?); Weight loss | Hernández-Secorún et al. (2021) reviewing 7 aerobic-type training programmes, finding most of them to be effective than no-treatment controls for function, pain, and balance in DPN. Positive findings for both HIIT and moderate intensity training. Also, treadmill compared favourably to balance training. No meta-analysis and primary studies not always great. | Provided depending on patient preference, access to equipment and training facilities, and disability levels. | Interactions with dietary components; May be facilitated through short-term relief from manual therapy and enabled through cognitive changes from CBT-based and pain education components (incl. goal setting techniques and value-driven action). |
| **Breathing exercises** | OR breathing re-training. Controlled diaphragmatic breathing, usually with specific duration of in- and outbreaths. | Stress-reduction (ANS); prefrontal control; Control over physical reaction to pain. |  | Potentially practiced during in-person sessions and provided as “homework” | Can be incorporated in all physical exercises and forms part of many mindfulness techniques; May increase ability to engage in movement through altered attention focus (from pain experience to breathing) |
| **Communication, Education, and ACT-informed components** | | | | | |
| **Pain self-management advice** | Advice on rest, ice, heat, over-the-counter medication, and physical activity. | Pharmacological analgesia during flare-ups; Local and central effects on nociceptive processing. |  | Providers have access to list of best-practice advice for MSK and neuropathic pain flare-ups. Can be provided verbally and / or via an information sheet. | May complement intervention sessions by easily applicable, active, and healthy coping strategies. Basis of more specific exercise / regular engagement in activities. Links with pain education to understand value of active coping strategies and rationale for pharmacological pain relief in facilitating movement and (possibly) preventing chronification. |
| **General physical activity guidance** | Recommendations on starting or modifying physical activities. May include daily walking, taking stairs, interrupt sedentary periods, or exercise programmes such as gym, yoga, swimming, cycling, speed walking running, etc.; Could consider use of accelometre / pedometric app.  Technique: Can be delivered in 5A’s manner (Estabrooks et al., 2003), a structured communication approach to evaluate and promote physical activity in primary care. | - slow muscle atrophy  - maintain gait mechanics  - reduce risk of falls  - cardiovascular benefits and enhanced glycaemic control  Statement of the American Diabetes Accoc.: “*Exercise improves blood glucose control in type 2 diabetes, reduces cardiovascular risk factors, contributes to weight loss, and improves well-being. Regular exercise may prevent or delay type 2 diabetes development. Regular exercise also has considerable health benefits for people with type 1 diabetes (e.g., improved cardiovascular fitness, muscle strength, insulin sensitivity, etc.).”* Should be tailored to individual needs (Colberg et al., 2016) | Activity promotion studied often alongside dietary intervention in diabetes risk prevention (Balk et al., 2015). Recent 3-armed RCT found little benefit on metabolic function from pedometer-based activity promotion, with or without additional group support for behaviour change and diabetes specialist 1:1 support, and any effects waning after intervention termination (Rossen et al., 2021). Reviews conclude that pedometers increase activity levels, with less evidence to suggest metabolic improvements (Baskerville et al., 2017; Franssen et al., 2020), and some indication that combination with in-person consultation may add benefit (Hodkinson et al., 2021). | Aim could be to build on patient’s preferences and existing habits. Needs to be individualised; “Advice” would be provided using below CBT-informed communication strategies. | Hoped to be enhanced through osteopaths’ training in CBT-informed techniques. |
| **Mindfulness-based Stress Reduction, MBSR** | Meditation, Rest, Breathing … | Stress-reduction (ANS); prefrontal control; empowerment | High-intensity 8-week group programme improved function, better health-related quality of life, and reduced pain intensity, pain catastrophizing, and depression compared to those receiving usual care (individual RCT, pDPN, n = 66) (Nathan et al., 2017). | Self-management component; Instructed through provider / other resources |  |
| **Education (Pain Education)** | Learning about pain mechanisms; experiential and individualised components (incl. reflection on own experience); Neuropathic pain-related information | Reducing kinesiophobia and catastrophizing. Possibly reframing threat value of pain. | Systematic review finding improvements in kinesophobia and catastrophising but not pain intensity or quality of life (Watson et al., 2019). Also see (Ashar et al., 2021) regarding these cognitive mediators. | As part of provider interaction at outset (to set the scene for improved pain self-management); As (optional) component provided through external resources (online) | Possibly allowing for better engagement in physical activity and / or manual therapy through reductions in negative pain connotations. |
| **Sleep Management (Education)** | Learning about best-practice strategies to improve sleep | Addressing one of the most salient QoL disturbances (as identified during PPI and congruent with literature (Ferini-Strambi, 2017; Naranjo et al., 2019)) | … | Provided as simple hand-out and discussed with provider | Possibly buffering the impact of NeuP on sleep and addressing relevant effect of symptom; thereby making intervention more logical and hopefully effective. |
| **Acceptance and Comittment Therapy (ACT) – informed components** | Techniques / exercises (from Scott & McCracken, n.d., ACT OPEN workbook), selection:  1. Focus on your breathing (Mindfulness)  2. Passenger on the bus Metaphor and reflective questions (Shifting attentional focus)  3. Notice five things (Awareness)  4. Unwanted party guest Metaphor and reflective questions (Openness)  5. Observe-Breathe-Open Up Meditation (combination of skills)  6. Walking Meditation | Mechanisms:  Learning to tolerate distressing experience and to learn self-acceptance (Lau and McMain, 2005)  More control over emotional aspect of pain experience | Moisset et al. (2020) included five open-label RCTs of psychological studies in their review on pain in (any) peripheral neuropathy, concluding: There is a weak recommendation for the use of CBT and mindfulness as an add-on therapy for neuropathic pain (moderate final quality of evidence).  Another review (Racaru et al., 2021) included nine RCTs with both TAU and attention control comparators. Meta-analyses of 2-4 studies each showed significant effects of interventions over controls for short- and medium-term pain intensity, pain interference, and depressive symptoms, and for short-term quality of life. | Several:  1. Combined mindfulness-based exercises during manual treatment  2. Dedicated mindfulness exercises during treatment session, such as body scans etc.  3. Homework-based practice possible for all (if programme inflexible) / some patients (if individualised)  In training programme:  Cultivate accepting and mindful provider state of mind (provider practice?) | Promoting an ‘aware mode’ characterised by a freedom of choice (Lau and McMain, 2005), possibly allowing for increased activity and engagement despite pain, fear of pain, or associated negative thoughts.  May interact with pain and psychoeducation, “about [pain]-related thoughts and symptoms can facilitate earlier detection of these experiences, thereby increasing the chance of timely interventions like a previously created relapse-prevention plan.” (Lau and McMain, 2005), p. 865 |
| **Relaxation (PMR)** | Guided sequence of contraction and relaxation of subsequent muscle groups. | Stress-reduction (ANS) | (Izgu et al., 2020) 3-arm parallel-group trial of 12-week self-managed PMR vs mindfulness vs single-session educational attention control (T2DM with NeuP, n total = 77), finding sign. b/w-group differences in favour of PMR and MM over control for pain intensity but not QoL. | Step-by-step protocol provided as audio-file onto patient’s phone (see Izgu et al. supplement). Could be standard component or according to patient preference. |  |
| **Mindfulness meditation** | Guided mindfulness tasks involving sustained awareness of bodily sensations, breathing, etc. | Stress-reduction (ANS); prefrontal control; | (Izgu et al., 2020), see above.  Evidence synthesis of *brief* mindfulness-based interventions on pain-related outcomes is inconclusive (McClintock et al., 2019). Longer programmes seem not to have consistent effects on pain, QoL, or mental health in acute or chronic pain (Ball et al., 2017); Effects on physical function in chronic pain are questionable (Jackson et al., 2019). No evidence of effects from n=20 pilot by Teixeira (2010): After 4-week pre-recorded meditation programme compared to nutrition diary for neuropathic pain, NP-related quality of life, and sleep quality. |  | Mindfulness elements could also be integrated into manual and exercise-based intervention components. Refocus of attention from pain experience to, eg, breathing may facilitate engagement in physical activity despite pain. Supported by positive effects of pain education and CBT / goal setting. |
| **Diabetes management components** | | | | | |
| **Diabetes complication screening and management** | Providers trained to routinely screen for diabetes complications and doing so at regular intervals.  Providers trained to recognise hypo and deliver hypo treatment in emergency situations | Prevention of complications and / or aggravation. | Part of NICE guideline for Type 2 Diabetes (NICE, 2015). Further guidelines exist for individual complications. | Incorporated into baseline assessments and regular check-ups. | Hoped to enhance therapeutic relationship through professionalism and reduce risk of disease complications alongside more active components. Comply with best-practice guidelines; role as first contact practitioner; Raising patients’ awareness to signs and symptoms of potential complications (linked to education components). |
| **Education (Diabetes and its complications)** | Providers trained in pathophysiological knowledge and clinical presentations; |  | Systematic review of 42 RCTs finding that self-management education reduces all-cause mortality risk compared to usual care (He et al., 2017) | Could be short checking in with patient regarding their disease understanding and addressing of misconceptions if present | Basic knowledge to facilitate communication and management, and enhance therapeutic relationship. |
| **Dietary components** | | | | | |
| **Dietary (Advice or Planning)** | Intervention could range from simple encouragement to change eating habits, to online education session or peer-support group, to individualised dietary plans. |  |  | Basic checking of dietary habits and existing management strategies; qualified comment or further advice only if needed.  Could also be (optional) online or group-based component. | Similar to above, but could play larger role and be easier / more relevant to deliver |
| **Integration with standard medical care** | | | | | |
| **Integration with pharmacological and other medical DM management** | Acknowledgement of medication list; Provider awareness of common side effects and prescribing guidelines. Communication with prescribing clinician if side effects suspected and desired by patient.  Providers aware of standard medical management, incl. medications and side effects.  Providers know the standard clinical steps / stages of patient management and can communicate with respective specialists. | Potential removal of side-effect-producing medication. |  | Part of initial consultation. Need to acknowledge professional boundaries and competence of osteopaths. Medication changes could be considered as exploratory outcome measure in feasibility study, will have to be monitored as potential confounder / mechanisms during definite trials. | Hoped to enhance patients’ trust in provider competency as well as ability to communicate with other medical professionals if needed. Knowledge of common medications and their potential side-effects relates to education and choice of self-management advice; Pain relief from other sources maybe allowing for reduction in medication. |
| **Integration with pharmacological and other medical pain management** |  |  |  |  |  |
| **Excluded elements** | | | | | |
|  | **Justification** |  |  |  |  |
| **High-velocity thrusts to the neck**  (Cervical manipulation with a rotary component, taking the neck beyond its normal range**)** | To limit any risk of serious adverse effects and because not indicated for pDPN |  |  |  |  |
| **Printed or digital materials outside the dedicated NeuOst material** | To reduce heterogeneity during a trial and avoid possible contradiction with NeuOst management approach |  |  |  |  |
| **Conservative treatment modalities occasionally provided by osteopaths**: Acupuncture, bed rest, biofeedback, electrotherapy. | To reduce heterogeneity during a trial and avoid possible contradiction with NeuOst management approach |  |  |  |  |
| **Appliances and devices**:  Belts, Straps, Braces, Splints, Tape, Massage guns, Laser and Ultrasound, Digital Therapeutics, Wellness smartphone or PC applications.  (**Not excluded** is NeuP-relevant advice on e.g., compression gloves or socks, heated or low-sensory mittens, wrist splints for Carpal Tunnel Syndrome, desensitization and stereognosis exercises (Andersen Hammond et al., 2020)) | To reduce heterogeneity during a trial and avoid possible contradiction with NeuOst management approach  To maintain pragmatic treatment provision |  |  |  |  |

*Note: Consider splitting programme theory into core components and co-interventions for the purpose of trial and control intervention design*

### Intervention elements per treatment session (treatment protocol for feasibility trial)

The intervention components will be delivered in a step-wise programme, delivered over six sessions. This approach was chosen to provide practitioners with an initial structure to deliver NeuOst. It will also facilitate high-fidelity intervention delivery during the feasibility trial. Clinically, graded exposure to physical activity, a step-by-step approach to psychological interventions, and repeated sessions of manual therapy are also common.

Briefly, target mechanisms of the NeuOst intervention are (detailed in above table):

- Behaviour change towards more physical activity
- Lower extremity muscle strength and sensorimotor control
- Cardiovascular fitness and downstream glucose metabolism
- Behaviour change towards more effective disease and complications management
- Cognitive-emotional changes towards more psychological flexibility and reduction of unhelpful pain beliefs
- Lower extremity joint mobility, soft tissue relaxation, and sensory input
- Autonomic nervous system control / stress reduction

| **Intervention content** (providers decide the sequence per session)  *Components marked ‘optional’ are non-obligatory; all others must be delivered as part of the respective treatment session.* | **Fidelity checklist**  (to be completed by trial providers) | **Notes** (Further detail such as amount and duration or provider experiences such as problems and opportunities) |
| --- | --- | --- |
| **Session 1** |  | Session duration up to 90 mins |
| Communication elements:   1. **Active listening to patient narrative** 2. **Initial case history** 3. **Introduction to the therapeutic objectives and structure of NeuOst** 4. **Acknowledgement of medication list** 5. Conversation about medications (optional) 6. Communication with primary care / specialist care provider (optional) 7. Signposting to DM/NeuP-related care options (optional) 8. Exploration of patient beliefs (regarding pain and disease) (optional) 9. Education about pain and neuropathic pain, including management strategies, sleep management, and devices (optional)   ACT-informed elements:   1. **Exploration of currently employed pain control strategies** (via Session 1 ACT worksheet) 2. **Exploration of what matters to patient** (via Session 1 ACT worksheet) 3. **Explorations of “away” and “toward moves”** (via Session 1 ACT worksheet) 4. **Exploration of physcial activities and connection to what is important to patient** | Comms:              ACT: | Up to 30 mins |
| Manual therapy:   1. **Assessment** (inspection, active & passive joint movement, palpation) 2. **Peripheral joint articulation** (feet & ankles at least 2mins per side to end range-of-motion; then knees & hips any duration) 3. Other peripheral joint articulation (optional) 4. Spinal joint articulation (optional) 5. Spinal joint manipulation, except cervical spine (optional) 6. **Peripheral soft tissue manipulation** (gastrocnemius at least 1min per side) 7. Other peripheral soft tissue manipulation (optional) 8. Paraspinal soft tissue manipulation (optional) 9. **Active resisted muscle activation** (lower extremity: dorsiflexion, plantarflexion, knee flexion and knee extension, at least 10 times each per side, hold 5 seconds per rep.) 10. **Neurodynamic technique lower extremity** (active; 10 reps per side) 11. ‘Subtle’ osteopathic techniques (optional) 12. Integration of patient breathing, relaxation, interoceptive awareness (“notice” and “open”), and/or values- and activity-exploration (ACT-based) into manual treatment (optional) | MT: | Up to 20 mins |
| Neurological:   1. **Provider baseline neurological examination** (in addition to research-related baseline testing; not used as outcome) | Neuro: | Up to 10 mins |
| Physical activity:   1. **Raise the topic of physical activity with permission (use motivational interviewing techniques throughout)** (5As’: “Ask”) 2. **Evaluation of current physical activity levels** (“Assess”) 3. **Explore barriers to more engagement in physical activity** 4. **Educate about benefits of different types of physical activity** (“Advise”) 5. **Provide “Exercise and diabetes” handout** (by the Diabetes Research & Wellness Foundation) (“Advise”) 6. **Values-based goal setting for aerobic exercise activity** (patient choice of activity, use Borg scale for exertion level) (“Agree”) 7. **Practice session 1 home-based sensorimotor, stretch, strength (SSS) programme content** (have patient go through all exercises and reps each time!) 8. Decide on suitable modifications for SSS programme (optional) 9. **Provide exercise plan handout / link** (“Arrange”) 10. **Provide “wobble cushion”** (provided as part of trial) | Exx: | Up to 20 mins |
| Diabetes related:   1. **Diabetes complication screening** (red flags for foot health, vision changes, kidney disease etc.) 2. Conversation about dietary DM management (optional) 3. Education about DM (optional) 4. Education about DPN and other complications of DM, including management strategies (optional) 5. Provision of NeuOst educational materials (optional) | DM/DPN: | Up to 10 mins |
| Home programme content:  *Cardio:*  (Duration or distance, and level of exhaustion agreed in session and documented on handout; grade difficulty using Borg Rating Scale of Perceived Exertion to achieve “somewhat hard” to “hard” physical activity [13-15/20])   1. 3x p.w.: Engage in agreed amount of aerobic activity   *SSS (sensorimotor, stretch, strength) exercises:*  (Possible modification: support with handhold on desk etc if needed – safety; Eyes open vs. closed for difficulty; With or without wobble board)   1. 2x p.d.: 10x slump ‘tensioner’ neurodynamic movement 2. 1x p.d.: 10x sit-to-stand 3. 30-60 secs: Wobble cushion exercise (modifiable from simple two-legged standing to single-leg reaching) |  |  |
| **Also see next page!**  **PROVIDERS TO COMPLETE AT END OF SESSION:**  Please rate your agreement with the following statement with regards to today’s intervention session for the management of the patient’s painful diabetic neuropathy symptoms.  *This treatment will be completely effective.*     \| Strongly disagree \| Moderately disagree \| Slightly disagree \| Neither agree nor disagree \| Slightly agree \| Moderately agree \| Strongly agree \| \| --- \| --- \| --- \| --- \| --- \| --- \| --- \| \|  \|  \|  \|  \|  \|  \|  \| | | |

| **Session 2** |  | 45-60 mins duration |
| --- | --- | --- |
| Communication:   1. **Active listening to patient narrative** 2. **Follow-up case history** 3. Conversation about medications (optional) 4. Communication with primary care / specialist care provider (optional) 5. Signposting to DM/NeuP-related care options (optional) 6. Exploration of patient beliefs (regarding pain and disease) (optional) 7. Education about pain and neuropathic pain, including management strategies, sleep management, and devices (optional)   ACT-informed elements:   1. **Revisiting last week’s exploration of what matters to patient and away/towards moves** 2. **Exploring the problem with control via “Beach ball metaphor”** (via Session 2 ACT worksheet) 3. **“Notice 5 things” exercise** (via Session 2 ACT worksheet) 4. **Encouragement to practice awareness exercise at home** |  | Up to 20 mins |
| Manual therapy:   1. **Assessment** (inspection, active & passive joint movement, palpation) 2. **Peripheral joint articulation** (feet & ankles at least 2mins per side to end range-of-motion; then knees & hips any duration) 3. Other peripheral joint articulation (optional) 4. Spinal joint articulation (optional) 5. Spinal joint manipulation, except cervical spine (optional) 6. **Peripheral soft tissue manipulation** (gastrocnemius at least 1min per side) 7. Other peripheral soft tissue manipulation (optional) 8. Paraspinal soft tissue manipulation (optional) 9. **Active resisted muscle activation** (lower extremity: dorsiflexion, plantarflexion, knee flexion and knee extension, at least 10 times each per side, hold 5 seconds per rep.) 10. **Neurodynamic technique lower extremity** (active; 10 reps per side) 11. ‘Subtle’ osteopathic techniques (optional) 12. Integration of patient breathing, relaxation, interoceptive awareness (“notice” and “open”), and/or values- and activity-exploration (ACT-based) into manual treatment (optional) |  | Up to 20 mins |
| Neurological:   1. Follow-up neurological examination (if indicated, optional) |  |  |
| Physical activity:   1. **Revisit experiences and/or exercises of SSS programme** 2. **Explore if exercises and stretches can now be performed at greater difficulty level or through larger range of movement** 3. Reinforce importance of exercise (optional) 4. Explore barriers to adherence and provide advice on overcoming those (optional) 5. **Practice session 2 home-based sensorimotor, stretch, strength (SSS) programme content** (have patient go through all exercises and reps each time!) 6. Decide on suitable modifications for SSS programme (optional) 7. **Ensure patient has access to exercise plan** (“Arrange”) |  | Up to 10 mins |
| Diabetes management and education:   1. **Foot care education and practical run-through** (under guidance of below handout) 2. **Provide foot care handout** (“Diabetes and loking after your feet” from Diabetes UK) 3. Conversation about dietary DM management (optional) 4. Education about DM (optional) 5. Education about DPN and other complications of DM, including management strategies (optional) 6. Provision of other NeuOst educational materials (optional) |  | Up to 10 mins |
| **Home programme content Week 2:**  *Cardio:*   1. 3x p.w.: Engage in agreed amount of aerobic activity   *SSS (sensorimotor, stretch, strength) exercises:*   1. 2x p.d.: 10x slump ‘tenser’ neurodynamic movement 2. 1x p.d.: 10x sit-to-stand 3. 30-60 secs: Wobble cushion exercise (modifiable from simple two-legged standing to single-leg reaching) 4. **1x p.d.: 10x heel-to-toe raises (new)** 5. **1x p.d.: 10x per side: Standing knee raises or Step-up-and-down on stairs (new)** |  |  |
| **PROVIDERS TO COMPLETE AT END OF SESSION:**  Please rate your agreement with the following statement with regards to today’s intervention session for the management of the patient’s painful diabetic neuropathy symptoms.  *This treatment will be completely effective.*     \| Strongly disagree \| Moderately disagree \| Slightly disagree \| Neither agree nor disagree \| Slightly agree \| Moderately agree \| Strongly agree \| \| --- \| --- \| --- \| --- \| --- \| --- \| --- \| \|  \|  \|  \|  \|  \|  \|  \| | | |

| **Session 3** |  | 45-60 mins duration |
| --- | --- | --- |
| Communication:   1. **Active listening to patient narrative** 2. **Follow-up case history** 3. Conversation about medications (optional) 4. Communication with primary care / specialist care provider (optional) 5. Signposting to DM/NeuP-related care options (optional) 6. Exploration of patient beliefs (regarding pain and disease) (optional) 7. Education about pain and neuropathic pain, including management strategies, sleep management, and devices (optional)   ACT-informed elements:   1. **Reviewing patient experiences with “Noticing 5 things” exercise** 2. **Providing information on values vs goals** (via Session 3 ACT worksheet) 3. **Completing “Values Compass” and setting SMART goal** (Session 3 ACT worksheet) |  | Up to 30 mins |
| Manual therapy:   1. **Assessment** (inspection, active & passive joint movement, palpation) 2. **Peripheral joint articulation** (feet & ankles at least 2mins per side to end range-of-motion; then knees & hips any duration) 3. Other peripheral joint articulation (optional) 4. Spinal joint articulation (optional) 5. Spinal joint manipulation, except cervical spine (optional) 6. **Peripheral soft tissue manipulation** (gastrocnemius at least 1min per side) 7. Other peripheral soft tissue manipulation (optional) 8. Paraspinal soft tissue manipulation (optional) 9. **Active resisted muscle activation** (lower extremity: dorsiflexion, plantarflexion, knee flexion and knee extension, at least 10 times each per side, hold 5 seconds per rep.) 10. **Neurodynamic technique lower extremity** (active; 10 reps per side) 11. ‘Subtle’ osteopathic techniques (optional) 12. Integration of patient breathing, relaxation, interoceptive awareness (“notice” and “open”), and/or values- and activity-exploration (ACT-based) into manual treatment (optional) |  | Up to 20 mins |
| Neurological:   1. Follow-up neurological examination (if indicated, optional) |  |  |
| Physical activity:   1. **Revisit experiences and/or exercises of SSS programme** 2. **Explore if exercises and stretches can now be performed at greater difficulty level or through larger range of movement** 3. Reinforce importance of exercise (optional) 4. Explore barriers to adherence and provide advice on overcoming those (optional) 5. **Practice session 3 home-based sensorimotor, stretch, strength (SSS) programme content** (have patient go through all exercises and reps each time!) 6. Decide on suitable modifications for SSS programme (optional) 7. **Ensure patient has access to exercise plan (“Arrange”)** |  | Up to 10 mins |
| Diabetes management and education:   1. **Revisit content and experiences with foot care education and handout** 2. Conversation about dietary DM management (optional) 3. Education about DM (optional) 4. Education about DPN and other complications of DM, including management strategies (optional) 5. Provision of other NeuOst educational materials (optional) |  | Up to 10 mins |
| **Home programme content Week 3:**    *Cardio:*   1. 3x p.w.: Engage in agreed amount of aerobic activity   *SSS (sensorimotor, stretch, strength) exercises:*   1. 2x p.d.: 10x slump ‘tenser’ neurodynamic movement 2. 1x p.d.: 10x sit-to-stand 3. 30-60 secs: Wobble cushion exercise (modifiable from simple two-legged standing to single-leg reaching) 4. 1x p.d.: 10x heel-to-toe raises 5. 1x p.d.: 10x per side: Standing knee raises or Step-up-and-down on stairs 6. **1x p.d.: 10x: Kneeling lunges or Getting-up-from-floor, with or without support (new)** |  |  |
| **PROVIDERS TO COMPLETE AT END OF SESSION:**  Please rate your agreement with the following statement with regards to today’s intervention session for the management of the patient’s painful diabetic neuropathy symptoms.  *This treatment will be completely effective.*     \| Strongly disagree \| Moderately disagree \| Slightly disagree \| Neither agree nor disagree \| Slightly agree \| Moderately agree \| Strongly agree \| \| --- \| --- \| --- \| --- \| --- \| --- \| --- \| \|  \|  \|  \|  \|  \|  \|  \| | | |

| **Session 4** |  | 45-60 mins duration |
| --- | --- | --- |
| Communication:   1. **Active listening to patient narrative** 2. **Follow-up case history** 3. Conversation about medications (optional) 4. Communication with primary care / specialist care provider (optional) 5. Signposting to DM/NeuP-related care options (optional) 6. Exploration of patient beliefs (regarding pain and disease) (optional) 7. Education about pain and neuropathic pain, including management strategies, sleep management, and devices (optional)   ACT-informed elements:   1. **Revisiting last week’s SMART goal** (incl. any progress or barriers) 2. **Practicing “opening up” skill via “Leaves on a stream” exercise** (via Session 3 ACT worksheet) 3. **Encouragement to practice “openness” exercises at home** |  | Up to 20 mins |
| Manual therapy:   1. **Assessment** (inspection, active & passive joint movement, palpation) 2. **Peripheral joint articulation** (feet & ankles at least 2mins per side to end range-of-motion; then knees & hips any duration) 3. Other peripheral joint articulation (optional) 4. Spinal joint articulation (optional) 5. Spinal joint manipulation, except cervical spine (optional) 6. **Peripheral soft tissue manipulation** (gastrocnemius at least 1min per side) 7. Other peripheral soft tissue manipulation (optional) 8. Paraspinal soft tissue manipulation (optional) 9. **Active resisted muscle activation** (lower extremity: dorsiflexion, plantarflexion, knee flexion and knee extension, at least 10 times each per side, hold 5 seconds per rep.) 10. **Neurodynamic technique lower extremity** (active; 10 reps per side) 11. ‘Subtle’ osteopathic techniques (optional) 12. Integration of patient breathing, relaxation, interoceptive awareness (“notice” and “open”), and/or values- and activity-exploration (ACT-based) into manual treatment (optional) |  | Up to 20 mins |
| Neurological:   1. Follow-up neurological examination (if indicated, optional) |  |  |
| Physical activity:   1. **Revisit experiences and/or exercises of SSS programme** 2. **Explore if exercises and stretches can now be performed at greater difficulty level or through larger range of movement** 3. Reinforce importance of exercise (optional) 4. Explore barriers to adherence and provide advice on overcoming those (optional) 5. **Practice session 4 home-based sensorimotor, stretch, strength (SSS) programme content** (have patient go through all exercises and reps each time!) 6. Decide on suitable modifications for SSS programme (optional) 7. **Ensure patient has access to exercise plan (“Arrange”)** |  | Up to 10 mins |
| Diabetes management and education:   1. Conversation about dietary DM management (optional) 2. Education about DM (optional) 3. Education about DPN and other complications of DM, including management strategies (optional) 4. Provision of other NeuOst educational materials (optional) |  | Up to 10 mins |
| **Home programme content Week 4:**    *Cardio:*   1. 3x p.w.: Engage in agreed amount of aerobic activity   *SSS (sensorimotor, stretch, strength) exercises:*   1. 2x p.d.: 10x slump ‘tenser’ neurodynamic movement 2. 1x p.d.: 10x sit-to-stand 3. 30-60 secs: Wobble cushion exercise (modifiable from simple two-legged standing to single-leg reaching) 4. 1x p.d.: 10x heel-to-toe raises 5. 1x p.d.: 10x per side: Standing knee raises or Step-up-and-down on stairs 6. 1x p.d.: 10x: Kneeling lunges or Getting-up-from-floor, with or without support 7. **1x p.d.: 10x per side: Star-steps (front, side, back large steps) (new)** |  |  |
| **PROVIDERS TO COMPLETE AT END OF SESSION:**  Please rate your agreement with the following statement with regards to today’s intervention session for the management of the patient’s painful diabetic neuropathy symptoms.  *This treatment will be completely effective.*     \| Strongly disagree \| Moderately disagree \| Slightly disagree \| Neither agree nor disagree \| Slightly agree \| Moderately agree \| Strongly agree \| \| --- \| --- \| --- \| --- \| --- \| --- \| --- \| \|  \|  \|  \|  \|  \|  \|  \| | | |

| **Session 5** |  | 45-60 mins duration |
| --- | --- | --- |
| Communication:   1. **Active listening to patient narrative** 2. **Follow-up case history** 3. Conversation about medications (optional) 4. Communication with primary care / specialist care provider (optional) 5. Signposting to DM/NeuP-related care options (optional) 6. Exploration of patient beliefs (regarding pain and disease) (optional) 7. Education about pain and neuropathic pain, including management strategies, sleep management, and devices (optional)   ACT-informed elements:   1. **Exploring experiences with practicing “Leaves on a stream” since last session** 2. **Reviewing any skills practice patient may be performing** (incl. impact on life, opportunities for further practice, barriers and how to manage those moving forward) 3. **Review session 3 SMART goal** (incl. any progress towards that) 4. **Discussing how to deal with difficulties** |  | Up to 20 mins |
| Manual therapy:   1. **Assessment** (inspection, active & passive joint movement, palpation) 2. **Peripheral joint articulation** (feet & ankles at least 2mins per side to end range-of-motion; then knees & hips any duration) 3. Other peripheral joint articulation (optional) 4. Spinal joint articulation (optional) 5. Spinal joint manipulation, except cervical spine (optional) 6. **Peripheral soft tissue manipulation** (gastrocnemius at least 1min per side) 7. Other peripheral soft tissue manipulation (optional) 8. Paraspinal soft tissue manipulation (optional) 9. **Active resisted muscle activation** (lower extremity: dorsiflexion, plantarflexion, knee flexion and knee extension, at least 10 times each per side, hold 5 seconds per rep.) 10. **Neurodynamic technique lower extremity** (active; 10 reps per side) 11. ‘Subtle’ osteopathic techniques (optional) 12. Integration of patient breathing, relaxation, interoceptive awareness (“notice” and “open”), and/or values- and activity-exploration (ACT-based) into manual treatment (optional) |  | Up to 20 mins |
| Neurological:   1. **Follow-up neurological examination (this week not optional)**   (for providers; not used as outcome during research study) |  | Up to 15 mins |
| Physical activity:   1. **Revisit experiences and/or exercises of SSS programme** 2. **Explore if exercises and stretches can now be performed at greater difficulty level or through larger range of movement** 3. Reinforce importance of exercise (optional) 4. Explore barriers to adherence and provide advice on overcoming those (optional) 5. **Practice previous sessions’ home-based sensorimotor, stretch, strength (SSS) programme content** (have patient go through all exercises and reps each time!) 6. Decide on suitable modifications for SSS programme (optional) 7. **Ensure patient has access to exercise plan (“Arrange”)** |  | Up to 10 mins |
| Diabetes management and education:   1. **Explore patient’s plans for future DM and painful DPN management** 2. Conversation about dietary DM management (optional) 3. Education about DM (optional) 4. Education about DPN and other complications of DM, including management strategies (optional) 5. Provision of other NeuOst educational materials (optional) |  | Up to 10 mins |
| **Home programme content Week 5:** (same as previous week)    *Cardio:*   1. 3x p.w.: Engage in agreed amount of aerobic activity   *SSS (sensorimotor, stretch, strength) exercises:*   1. 2x p.d.: 10x slump ‘tenser’ neurodynamic movement 2. 1x p.d.: 10x sit-to-stand 3. 30-60 secs: Wobble cushion exercise (modifiable from simple two-legged standing to single-leg reaching) 4. 1x p.d.: 10x heel-to-toe raises 5. 1x p.d.: 10x per side: Standing knee raises or Step-up-and-down on stairs 6. 1x p.d.: 10x: Kneeling lunges or Getting-up-from-floor, with or without support 7. 1x p.d.: 10x per side: Star-steps (front, side, back large steps) |  |  |
| **PROVIDERS TO COMPLETE AT END OF SESSION:**  Please rate your agreement with the following statement with regards to today’s intervention session for the management of the patient’s painful diabetic neuropathy symptoms.  *This treatment will be completely effective.*     \| Strongly disagree \| Moderately disagree \| Slightly disagree \| Neither agree nor disagree \| Slightly agree \| Moderately agree \| Strongly agree \| \| --- \| --- \| --- \| --- \| --- \| --- \| --- \| \|  \|  \|  \|  \|  \|  \|  \| | | |
| End of programme |  |  |

## Elaboration of individual programme components

This section summarises the take-home messages from intervention table, with justification and brief overview of NeuOst content.

### Manual therapy

Massage, spinal manipulation, joint mobilisation, and other forms of manual therapy are poorly studied in people with painful diabetic neuropathy, or even peripheral neuropathies in general. Hernández-Secorún et al. (2021) summarised the current evidence as follows: “*As for manual therapy, there seems to be a short-term effect of the different types of massage after treatment in comparison with standard-care and treatment-free control groups, with high methodological quality of the studies*.” (p. 27-28). Whilst suggesting some effectiveness, it needs to be noted that this enthusiastic assessment is based on a heterogeneous sample of non-sham controlled small-to-medium-sized RCTs. For some forms of manual therapy, such as spinal manipulation, evidence relevant to peripheral neuropathies is lacking entirely. For others, such as neurodynamic stretching, it is based on individual trials only. Therefore, manual therapy elements included in NeuOst are only partly based on scientific evidence. Mainly, they are informed by involvement of osteopathic practitioners who have experience treating people with pDPN.

Manual elements of NeuOst include:

- Global joint **mobilization** sequences (“General osteopathic treatment”, GOT)
- Specific joint mobilization of lower extremity joints, notably ankle and foot joints
- Passive muscle **stretching** (“Muscle energy technique”, MET)
- **Spinal manipulation** of lumbar and thoracic spinal segments
- **Neurodynamic** passive stretching
- **Soft tissue** techniques (incl. cross-fibre massage, longitudinal stretch, direct pressure / trigger point inhibition, deep friction)
- **‘Subtle’** manual therapy techniques such as craniosacral or myofascial approaches
- **Active-resisted muscle strengthening** (active resistance programme, see image below)

Links to other NeuOst components:

- Mindfulness elements / biofeedback approach to passive manual therapy
- Integration with breathing exercises
- Transition to self-directed strengthening and stretching physical activity elements through active resistance and passive stretching elements

**Figure 5: Active resistance programme.** Exemplary indication of procedure principles and activities, and not exhaustive. In each movement, resistance is provided by the practitioner at a level to allow the patient to generate 5-6 RPE on the Borg scale.

### Physical activity and exercise

A statement of the American Diabetes Association holds that “*[e]xercise improves blood glucose control in type 2 diabetes, reduces cardiovascular risk factors, contributes to weight loss, and improves well-being. Regular exercise may prevent or delay type 2 diabetes development. Regular exercise also has considerable health benefits for people with type 1 diabetes (e.g., improved cardiovascular fitness, muscle strength, insulin sensitivity, etc.).*” In their document, the ADA lists a large range of metabolic changes and clinical benefits of increased physical activity relevant for blood sugar control and reduced risk of diabetes complications. The authors further highlight that any activity programme should be tailored to individual needs (Colberg et al., 2016). The broad range of benefits for people with diabetes justifies the addressing of people’s activity levels in any multimodal programme; In the subgroup of people with painful diabetic neuropathy, most trials focus on improving balance through sensorimotor and other training, with some evidence to suggest effectiveness. Evidence of effectiveness on pain and nerve function is limited. Effects of any physical activity on disease markers of diabetes are as relevant in programmes with people with neuropathy as for people without. However, a patient’s ability to engage in exercise programmes may be limited due to pain, balance problems, or foot complications.

Mechanistically, patients’ prevalent pathophysiological changes, and associated symptoms or functional limitations, ought to guide intervention development. PPI and existing literature also point to the fear of falling as a major concern, requiring the possibility to adapt the training programme for safety and comfort.

Therefore, NeuOst includes exercise components that can be adapted to individual patients’ abilities, preferences, and access to equipment / facilities:

- General **education, advice, and encouragement of any physical activity**, with the opportunity to individualise behavioural change techniques and specifics of recommended activity
- Training programme for paced **aerobic** activities performed at home
- Standardised and progressive training programme for home-exercise, focusing on **sensorimotor** exercises, **strengthening**, and including **neurodynamic and general stretching**
- Provision of a ‘wobble cushion’ to facilitate balancing exercises and enable the addition of balance elements to all anaerobic exercises

The physical activity components will follow a pacing approach, adding intensity / duration and new exercises each week. It is hoped that patients can retain the principles of pacing for their activities after the termination of the study intervention. Decisions are made in a shared manner between patient and provider, the latter of which will also draw on ACT-informed communication and techniques throughout.

E.g. for balance programme:

­­­­­­
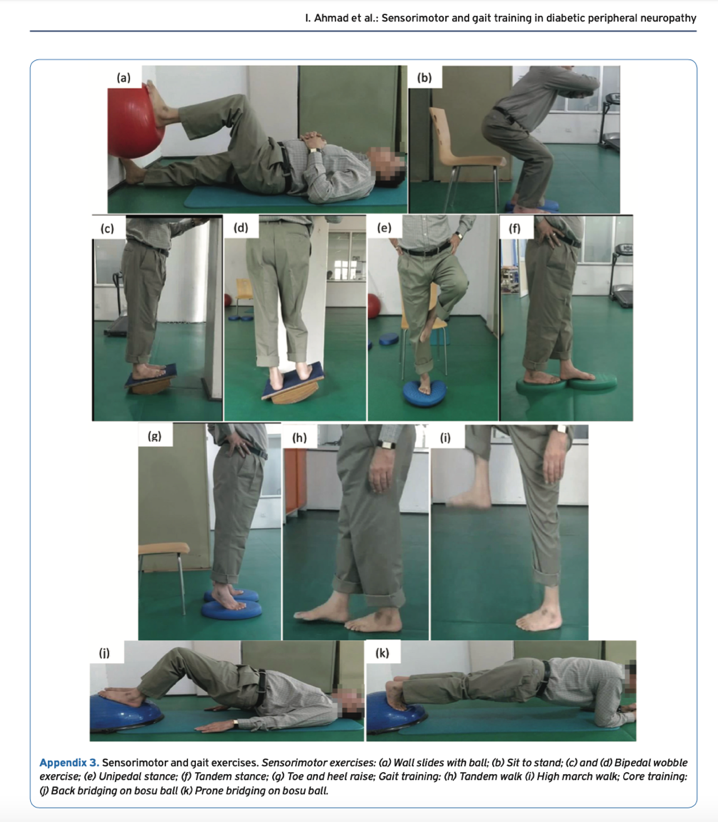

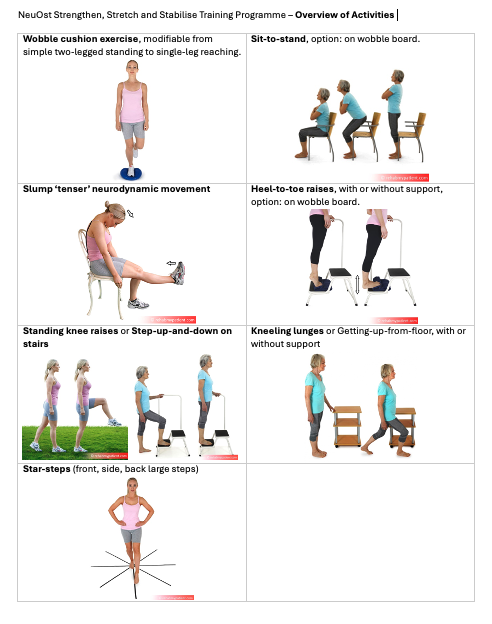


(image soruces: Rehab My Patient ®)

### Educational components for patients

In the context of NeuOst, several forms of patient education are relevant: General education on disease (diabetes) self-management and risk reduction is an integral part of guidelines and is supported by good evidence. Secondly, people with persistent pain may benefit from education about pain mechanisms (with little evidence for neuropathy-specific populations) and pain-related self-management strategies. Further potentially relevant informational components include dietary advice, sleep hygiene, ACT-informed concepts, and stress management.

Primarily, NeuOst providers will have to be able to integrate with the messaging patients receive from diabetes specialists such as nurses and doctors. Providers should be able to reinforce and support such messaging if needed. Secondly, osteopaths will have to be able to confidently answer questions related to pain mechanisms, self-management strategies, diet, sleep, and stress.

The patient-centred educational content of NeuOst will be delivered through providers in the treatment sessions, potentially supported by provision of educational material (leaflets / video links) and reinforced by discussions with providers during the in-person treatment sessions. Two leaflets will be provided to all patients prior to pre-defined treatments.

Core educational leaflets:

- “Exercise and diabetes” (Diabetes Research & Wellness Foundation)
- “Diabetes and loking after your feet” (Diabetes UK)

Optional educational videos / leaflets:

- Diet
- Sleep management
- Stress management
- Diabetes education, self-management, and risk reduction

### Psychological components

The rationales of psychological interventions in the context of persistent pain vary, including the addressing of negative thoughts and emotions around pain, promoting relaxation and mindful awareness, fostering self-efficacy, and facilitating behavioural change. The goal of many cognitive-behavioural interventions is to help people manage pain, distress and disability (Williams et al., 2020). In Acceptance and Commitment Therapy (ACT), a mechanism is to increase ‘psychological flexibility’, a concept comprising of psychological skills such as being acceptant, present, and engaging in value-driven actions (McCracken and Morley, 2014). Pain reduction is not commonly a direct aim of psychological interventions but may result from upstream mechanisms.

Further, persistent pain is often associated with other psychological problems (Ref) and so is pDPN (Kioskli et al., 2019a), making it relevant to screen for these concomitant presentations and include them into the management (namely depression, anxiety, and worse sleep and quality of life).

In a diabetic neuropathy population, behaviour change is relevant both for more effective diabetes management (which is heavily reliant on blood sugar control and thus diet, lifestyle change, and medication compliance), as well as for neuropathy-related aspects. The latter include engagement in beneficial activities for pain management as well as activities for risk reduction. Here, regular and conscientious foot care plays a major role.

For NeuOst, providers can be equipped with a) an understanding of psychological problems and cognitive processes potentially involved when living with persistent pain, and b) psychologically-informed tools to acknowledge these processes in their patient management. Further, providers need to be able to screen for psychiatric disease potentially requiring referral.

We draw on input from expert collaborators, work around psychologically-informed physiotherapy (Keefe et al., 2018), communication skills training, and experience from previous ‘augmentations’ of osteopathy with cognitive-behavioural techniques (Carnes et al., 2017). Overall, the NeuOst approach is to incorporate patient attitudes, beliefs, and emotional responses into patient management through deliberate provider actions, whilst facilitating the use simple cognitive-behavioural and other psychological skills by patients. Clinically, the aim is to reduce the impact of psychological barriers on well-being, quality of life, and beneficial value-determined behaviour.

NeuOst providers will be introduced to the psychological impact and psychotherapeutic concepts during a 1-hour lecture. They will be encouraged to engage in ACT-informed reflective exercises and mindfulness-based relaxation training themselves prior to attending the in-person training. At the in-person provider training event, a practitioner experienced in psychological interventions or psychologically-informed practice will facilitate communication training and engaging patients in ACT-informed techniques, including through role play. Providers will make further experience with the integration of mindfulness approaches into hands-on manual therapy sessions, and engage in reflective exercises themselves.

*Supervision*

Supervision through experienced providers plays an important role in psychotherapy training. Capacities for supervision during the NeuOst provider training phase are limited, but there will be intermittent supervision opportunities during the feasibility trial. A precedent for this approach is given by the Physical Therapy Informed by Acceptance and Commitment Therapy (PACT) study, where physiotherapists had monthly group supervision and authors commented that this “seems to have provide good support (…) to deliver PACT with high fidelity” (Godfrey et al., 2016, 2020).

## Provider training course

V 1.0, 08 March 2024 (final version for training course as delivered in January 2024)

Authors: Dr David Hohenschurz-Schmidt, Steve Vogel

**Course dates:** 27 January 2024, 9am until end, University College of Osteopathy, Borough High Street

**Delivery mode:** 1 day in person + Asynchronous e-learning materials to be provided beforehand + live e-learning with trial providers after in-person training day.

**Course Aims**

This course aims to equip fully qualified UK osteopaths with the knowledge and practical skills to work with people living with painful diabetic neuropathy, and to do so safely and in accordance with the NeuOst intervention theory.

Further, this course aims to prepare participants as intervention providers on the NeuOst feasibility study.

**Intended Learning Outcomes**

The overall intended learning outcomes (ILOs) of the provider training are as follows. Each individual lesson will define learning objectives that align with the broader ILOs of the course.

At the end of the NeuOst training course, providers will:

1. Understand disease mechanisms, pathophysiological changes, complications, and clinical presentations of Diabetes Mellitus (DM), Diabetic Polyneuropathy (DPN), and painful DPN (pDPN).
2. Understand current medical options and pathways for people with pDPN in the UK.
3. Appreciate the patient perspective, and the psycho-emotional and social impact of pDPN.
4. Be able to perform and interpret a bedside neurological examination specific for people with pDPN as well as complication screening for diabetic foot disease and complications.
5. Know the conceptual basics of Acceptance and Commitment therapy (ACT), be able to draw on simple ACT-based techniques in their communication and introduce patients to ACT-informed self-management techniques.
6. Have a plan for possible manual therapy techniques and their potential rationale when working with people with pDPN. -
7. Understand the benefits of exercise in pDPN and appreciate ways of guiding patients in a pDPN-focused graded exercise programme.
8. Be able to educate patients about the basics of DM, diabetes management, pain mechanisms in pDPN, pain self-management, foot care, and DM complication screening and management.
9. Appreciate the importance of maintaining blinding and balancing of contextual factors in clinical research and be able to implement the NeuOst feasibility trial’s test and control intervention with high fidelity and conviction.

Assessment

Whether course participants have achieved each session’s learning objectives will be assessed by means of multiple-choice assessments after the training course and through observations of practical exercises during the course

### Course plan

| **Pre-course materials (asynchronous e-learning)** | **In-person 1 (synchronous)** |
| --- | --- |
| Introduction to the NeuOst project and CPD course  Speaker: Dr David Hohenschurz-Schmidt  Duration: 25 mins  Delivery mode: Recorded.  Learning objectives:   - Familiarise yourself with the NeuOst project, including its objectives, underlying intervention development processes, the involved multidisciplinary team, and the basic design of the NeuOst feasibility trial. - Learn about the learning outcomes, content, and delivery mode of the NeuOst training course.   Content:   - Brief overview presentation | Introduction to the Practical Training Day  Speaker: Dr David Hohenschurz-Schmidt  Duration: 10 mins  Delivery mode: Face-to-face  Learning objectives:   - Reacquaint yourself with the Intended Learning Outcomes of the NeuOst course. - Learn about the practical procedures of the in-person day.   Content:   - Brief overview presentation of plan on the day - Fire safety - Q&A |
| Theory Lesson 1:  Diabetes pathophysiology, risks, and medical management  Speaker: Dr David Hohenschurz-Schmidt  Duration: 1 hour  Delivery mode: Recorded  Learning objectives:   - Gain a comprehensive understanding of the epidemiology, pathogenesis and pathophysiological changes associated with Diabetes Mellitus (DM) - Relate DM pathophysiology to common disease risks. - Appreciate medical management and risk screening procedures for T2DM, as aligned with current UK NICE guidelines.   Content:   - DM basics - Epidemiology and disease burden - Diagnosis - Risk factors and risk screening - Pathophysiology (T1, T2, Prediabetes) - Acute and chronic complications - T2DM treatment (NICE guidance & nutrition) - NHS management - T2DM prevention - Future directions - Resources | Practical Lesson 1:  Expert Q&A Session: Managing Patients with Diabetes and Diabetic Foot Disease  Speaker: *This session was not held in the course’s iteration on 27 Jan 2024 due to unsuccessful speaker recruitment. The overall course validity was not deemed to have been affected due to the largely experiential learning objectives (objective no. 3) and having covered objectives 1 and 2 in theoretical sessions and through Practical Lesson 4.*  Duration: 0.5 hours  Delivery mode: Face-to-face / Speaker via videocall  Learning objectives:   - Appreciate the clinical complexity of diabetes and its complications. - Understand the current best-practice pathways and their implementation in the NHS. - Engage with the clinician’s perspective on diabetes management.   Content:   - Semi-structured conversation and Q&A with the speaker. |
| Theory Lesson 2:  Diabetic Polyneuropathy  Speaker: Sasha Smith  Duration: 1 hour  Delivery mode: Recorded  Learning objectives:   - Understand the epidemiology and pathophysiology of DPN. - Recognize clinical presentations and complications. - Appreciate diagnostic criteria and methods. - Explore medical management and evidence of effectiveness.   Contents:   - Terminology - Prevalence - Risk factors - Cellular mechanisms - Pathophysiological changes - Symptoms - Complications - Diagnosis - Management | Theory Lesson 8:  An Brief Introduction to Acceptance and Commitment Therapy (ACT) for Persistent Pain and its Application within NeuOst  Speaker: Dr Whitney Scott  Duration: 1 hour  Delivery mode: face-to-face  Learning objectives (TBC):   - Understand key aspects of the psychological flexibility model and the focus within ACT. - Experience and practice ACT-based methods that will be delivered within NeuOst. - Support reflective practice and noticing one’s own responses and behaviours as a clinician.   Content:   - Psychosocial elements of living with persistent pain - Principles of ACT - Psychological risk screening and decision- and referral-processes |
| Theory Lesson 3:  Neuropathic Pain in Painful Diabetic Neuropathy  Speaker: Dr Harriet Kemp  Duration: 30 mins  Delivery mode: Recorded  Learning objectives:   - Understand the epidemiology and pathophysiology of neuropathic pain (NeuP) in general and painful DPN in particular. - Recognize clinical presentations and complications of pDPN. - Appreciate diagnostic criteria and methods. - Explore medical management and evidence of effectiveness.   Contents:   - Categorisation of Pain - Epidemiology - Physiology of pain & painful DPN - Clinical Presentation - Diagnostic Criteria - Medical Management | Practical Lesson 2  An Brief Practical Introduction to ACT for Persistent Pain and its Application within NeuOst  Speaker: Dr Whitney Scott  Duration: 1 hour  Delivery mode: Face-to-face  Learning objectives:   - Understand key aspects of the psychological flexibility model and the focus within ACT. - Experience and practice ACT-based methods that will be delivered within NeuOst. - Support reflective practice and noticing one’s own responses and behaviours as a clinician.   Content:   - Practical exercises of ACT-based methods. |
| Theory Lesson 4  Physical Activity and Exercise for People with Diabetes and Neuropathy  *Talk 1: The Role of Physical Activity and Exercise in the Management of Diabetes and DPN*  Speaker: Dr David Hohenschurz-Schmidt  Duration: 0.75 hour  Delivery mode: Recorded  Learning objectives:   - Appreciate neurophysiological and metabolic mechanisms through which physical activity and exercise provides benefits in Diabetes (DM) and Diabetic Neuropathy (DPN). - Understand the current evidence and official recommendations for various types of physical activity. - Appreciate the challenges of exercise prescription in people living with painful DPN. - Learn about the exercise component of the NeuOst programme.   Content:   - Principles of physical activity and therapeutic exercise - Exercise in DM - Exercise for DPN and neuropathic pain - Challenges of exercise prescription in pDPN - The NeuOst exercise component   *Talk 2: Principles of Exercise Adherence*  Speaker: Dr Daniel Bailey  Duration: 0.5 hour  Delivery mode: Recorded  Learning objectives:   - Understanding the concepts and importance of exercise adherence and its promotion in clinical practice. - Relate to and reflect on the challenges of exercise adherence from provider, patient, and system perspectives. - Appreciate the peculiar challenges of physical activity for peP2ople with multimorbidity, physical limitations, and for the elderly. - Learn about evidence-based principles and practical strategies of encouraging exercise adherence. - Apply the learned principles to hypothetical patient cases with painful Diabetic Neuropathy, developing exercise adherence strategies tailored to the specific challenges presented in these cases. - Engage in and reflect on the practical application of exercise adherence principles in participants’ next clinical session.   Content:  TBC | Practical Lesson 3:  The Neurological Assessment of People with Suspected Peripheral Neuropathy  Speaker: Dr Matthew Evans & Dr David Hohenschurz-Schmidt  Duration: 1 hour  Delivery mode: Hybrid: Recorded & Face-to-face  Learning objectives:   - Translate previously gained knowledge into a case history for people with suspected pDPN. - Understand the nature, rationale, and validity of common bedside screening tests. - Apply screening tests expertly.   Content:   - Go through questionnaire completion in roleplay. - Practical bedside neurological testing |
| Theory Lesson 5:  Manual Therapy in pDPN: Rationale and Evidence  Speaker: Dr David Hohenschurz-Schmidt  Duration: 0.5 hour  Delivery mode: Recorded  Learning objectives:   - Comprehend the Rationale Behind Manual Therapy in Painful Diabetic Neuropathy (pDPN): - Explore neurological and musculoskeletal changes in people living with pDPN that may be amenable to manual therapy. - Explore the potential mechanisms and target outcomes of manual therapy in pDPN. - Appreciate the views of manual therapy providers and of patients on the potential role of manual therapy in the management of people with pDPN. - Evaluate the Existing Evidence for Manual Therapy in pDPN. - Explore the Patient-Centred Application of Manual Therapy: - Learn about the core manual therapy techniques proposed as part of the NeuOst intervention. - Understand how manual therapy can be tailored to individual patients. - Appreciate how Manual Therapy Integrates with Other Components of the multimodal NeuOst Intervention. - Develop an Awareness of Potential Risks and Contraindications Associated with Manual Therapy in Individuals with pDPN.   Content:   - Changes in pDPN relevant for MT - Rationale for MT techniques in pDPN - Integration within NeuOst intervention - Research evidence and gaps - Provider and patient views - Techniques - Safety considerations | Practical Lesson 4:  Expert Q&A Session: The Experience of Living with DPN and Neuropathic Pain  Speaker: Mrs Elizabeth Piggott  Duration: 1 hour  Delivery mode: face-to-face  Learning objectives:   - Appreciate and reflect on the personal experience and impact of living with DPN and pDPN. - Engage with a person living with pDPN. - Reflect on the implications for clinical management.   Content:   - Informal presentation of patient partner - Open conversation / Q&A in plenum   - How did your condition develop?   - When and how does it affect you most?   - What do you do to manage it?   - What was / is your experience with your medical management?   - What have you learnt from talking to other people with this condition? |
| Theory Lesson 6 (**optional**):  Placebo Effects in Osteopathy  Speaker: Dr David Hohenschurz-Schmidt  Duration: 1 hour  Delivery mode: Recorded  Learning outcomes:   - Appreciate the historical development of the concepts: placebo, nocebo, placebo effects, and placebo response and understand their current use and challenges. - Understand the neurophysiological and psychological mechanisms underpinning placebo effects, especially regarding pain. - Relate the context-rich nature of osteopathic clinical practice to the potential for placebo and nocebo effects. - Reflect on the implications of the current science of the placebo effect for our mechanistic understanding and research of osteopathy and for clinical practice.   Content:   - Placebo and nocebo effects: description, science, and mechanisms - Implications for osteopathic practice, education, and research | Practical Lesson 5  Patient-Centred Communication when Working with People with Persistent Pain  Speaker: Steven Vogel  Duration: 2 hours  Delivery mode: Face-to-face  Learning objectives:   - To explore the practicalities and core communication skills when working in partnership with patients with persistent pain. - To explore opportunities to enhance person centred care and communication.   Content:   - Characteristics of person-centred care - Role plays with observer feedback and peer discussions. - Communication skills: Active listening, initiating the consultation, verbal and nonverbal communication, self-awareness. |
| Theory Lesson 7 (**only required from NeuOst trial providers**):  Nonpharmacological Intervention Research and the NeuOst Feasibility Trial  *Talk 1: The Fundamentals of Nonpharmacological Intervention Research and of Placebo Controlled Trials*  Speaker: DHS  Duration: 0.5 hours  Delivery mode: Recorded  Learning objectives:   - Appreciate the challenges of nonpharmacological / complex intervention clinical research, especially in so-called efficacy trials. - Appreciate the potential for placebo effects in physical and psychological interventions and their implications for trial design. - Understand the concepts of blinding and control interventions in efficacy trials. - Know about current best-practice frameworks for control intervention design.   Content:   - 30-mins recording of a webinar held for the British Pain Society - Includes an optional 30-mins talk by Prof David Beard on placebo-controlled surgery trials and a panel discussion.   *Talk 2: The NeuOst Trial Protocol and Considerations for Trial Providers*  Speaker: DHS  Duration: 0.5 hours  Delivery mode: Synchronous e-learning (live delivery after in-practice training day for selected trial providers)  Learning objectives:   - Understand the basic design elements NeuOst feasibility trial. - Appreciate the tasks and role of clinical providers in an RCT in general and in NeuOst in particular. - Reflect on and discuss concerns and challenges as trial providers. - Gain access to materials for providers, such as detailed session protocol and ACT handbook. - Commence practical feasibility trial participation planning, such as populating weekly diaries.   Content:   - Key design elements of the NeuOst feasibility trial - Role and responsibilities of clinical trial providers in sham-controlled trials in general and in the NeuOst feasibility trial in particular. - Experiences of trial providers from relevant related RCTs. - Relevant ethical considerations. - Reflection and discussion amongst participants. - Training tasks. | Practical Lesson 6  Manual Therapy and Exercise Components of NeuOst  Speaker: DHS  Duration: 1 hour  Delivery mode: Face-to-face  Learning objectives:   - Learn the content and practical delivery of NeuOst manual therapy components. - Learn the content and practical delivery of NeuOst physical activity components. - Understand the flexibility in the programme contents and how to tailor recommendations / content. - Practice how to integrate communication skills and psychologically informed methods into the delivery of manual and exercise components   Content:   - Summary of intervention theory and elaboration on intervention component interactions in theory and practice - Detailed introduction to the manual therapy and exercise components of NeuOst. - Practical on manual therapy and exercise administration amongst peers. - Reflection on challenges for NeuOst administration in clinical practice. |

### Aditional training for designated trial providers

For designated trial providers, the NeuOst training course included two obligatory lectures on 1) the Fundamentals of Nonpharmacological Intervention Research and of Placebo Controlled Trials, and 2) the NeuOst Trial Protocol and Considerations for Trial Providers, delivered by the chief investigator. This covered the following learning objectives and content. Course completion was ensured by the chief investigator and understanding was tested by means of a quiz.

Talk 1: The Fundamentals of Nonpharmacological Intervention Research and of Placebo Controlled Trials (Duration: 0.5 hours, Delivery mode: Recorded)

Learning objectives:

- Appreciate the challenges of nonpharmacological / complex intervention clinical research, especially in so-called efficacy trials.
- Appreciate the potential for placebo effects in physical and psychological interventions and their implications for trial design.
- Understand the concepts of blinding and control interventions in efficacy trials.
- Know about current best-practice frameworks for control intervention design.

Content:

- 30-mins recording of a webinar held for the British Pain Society (full recording: <https://www.youtube.com/watch?v=WI4jaqf5_PE>)
- Webinar includes an optional 30-mins talk by Prof David Beard on placebo-controlled surgery trials and a panel discussion.

Talk 2: The NeuOst Trial Protocol and Considerations for Trial Providers (Duration: 0.5 hours, Delivery mode: Synchronous e-learning - live delivery after in-practice training day for selected trial providers)

Learning objectives:

- Understand the basic design elements NeuOst feasibility trial.
- Appreciate the tasks and role of clinical providers in an RCT in general and in NeuOst in particular.
- Reflect on and discuss concerns and challenges as trial providers.
- Gain access to materials for providers, such as detailed session protocol and ACT handbook.
- Commence practical feasibility trial participation planning, such as populating weekly diaries.

Content:

- Key design elements of the NeuOst feasibility trial
- Role and responsibilities of clinical trial providers in sham-controlled trials in general and in the NeuOst feasibility trial in particular.
- Experiences of trial providers from relevant related RCTs.
- Relevant ethical considerations.
- Reflection and discussion amongst participants.
- Training tasks.

Finally, designated trial providers attended a practical 2-hour session with two patient partners, where the complete intervention and control intervention protocols were practised in small groups. Afterwards, providers reflected on the experience, facilitated by the chief investigator.

# Outlook: Further phases of intervention development

Having defined the core elements of *NeuOst*, this document now contextualises the intervention in the broader process model of the MRC, from intervention development, to feasibility, evaluation, and implementation.

## Developing NeuOst as a complex intervention

The above sections on the programme’s core elements have discussed the approach to the development of *NeuOst* extensively. At this stage, it is noteworthy that *NeuOst* is a newly developed intervention that, however, draws on the *adaptation* of existing approaches from other fields which are then used as components in a novel intervention. Adaptation is defined as "*the intentional modification of interventions to meet the needs of a new context, where there is an evidence-base of effectiveness in the original context*." (Evans et al., 2021, p. 41). The new context of, for example, cognitive behavioural or mind-body intervention components is given by the manual therapy context. Conversely, manual therapy components need to be adapted to the context of a new patient population.

Beyond adapting intervention components, this also must consider the potential need to modify aspects of the new context to accommodate the intervention. In our case, osteopathic practice may have to adapt to accommodate the new intervention components and patient population, as much as the introduced intervention components will have to be adapted to work within osteopathic practice. Importantly, in adapting interventions to new settings, core components need to be retained, referring to those "*features in the intent and design of an intervention deemed responsible for the effectiveness of the intervention*." (Evans et al., 2021, p. 41). At the same time, the "*misapplication or a mistaken application of an intervention involving technical errors, abandonment of core components or introduction of counterproductive elements resulting in a loss of intervention benefits*" (Evans et al., 2021, p. 41) (also called 'drift') needs to be considered as a threat to successful adaptation. As we progress through the intervention development, these risks will be considered by the research team and stakeholders, and potentially included as feasibility outcomes in the next stage.

### Feasibility

According to the MRC framework, "*[a] feasibility study should be designed to assess predefined progression criteria that relate to the evaluation design (e.g., reducing uncertainty around recruitment, data collection, retention, outcomes, and analysis) or the intervention itself (e.g., around optimal content and delivery, acceptability, adherence, likelihood of cost effectiveness, or capacity of providers to deliver the intervention). If the programme theory suggests that contextual or implementation factors might influence the acceptability, effectiveness, or cost effectiveness of the intervention, these questions should be considered*." (Skivington et al., 2021, p. 6). Key concepts of feasibility studies were defined by Eldridge et al. (2016), including for later-stage pragmatic trials (Chan et al., 2021), and reporting guidelines are available (Eldridge et al., 2016a).

A detailed feasibility trial protocol has been developed and is provided elsewhere.

### Evaluation

The new MRC framework emphasises the need for evaluation beyond efficacy/effectiveness, to include interactions between intervention and context, broader systemic impact, and questions of healthcare or policy decision-making (Skivington et al., 2021). In keeping with other methodological recommendations, efficacy questions will dominate initially (Hohenschurz-Schmidt, IMMPACT, almost submitted), but such trials may be accompanied by mixed-method evaluation of other potential impacts, also improving the understanding of how *NeuOst* might work, if it does.

### Implementation

Initially set in an osteopathic educational institution, successful implementation beyond this setting will consider the following:

- Interest in the *NeuOst* provider training programme once offered to the osteopathic community as Continued Professional Development course
- Compatibility with or adaptability to osteopathic private practice
- Interest of National Health Service decision-makers in adopting *NeuOst*
- Availability or development of alternative non-private funding models to enhance *NeuOst* accessibility
- Importantly, findings from earlier research stages and successful progression to an implementation stage

Promoting the chances of successful implementation, comprehensive stakeholder involvement and process evaluations will again be fundamental. For example, before expanding *NeuOst* beyond the academic realm, barriers and facilitators to the implementation in osteopathic private practice may have to be identified and mitigated.

### Economic considerations

“*Economic evaluation—the comparative analysis of alternative courses of action in terms of both costs (resource use) and consequences (outcomes, effects)—should be a core component of all phases of intervention research. Early engagement of economic expertise will help identify the scope of costs and benefits to assess in order to answer questions that matter most to decision makers.”* (Skivington et al., 2021, p. 6).

A formal cost-effectiveness analysis will not be possible at the early stage of feasibility testing since no reliable data on effectiveness will be gathered. We will, however, be able to specify the cost of provider training per individual (time for training development, testing, provision), and of the intervention delivery (including provider time cost, room rent, cost of used computer programmes and physical tools, administration costs). Indicators of variables relevant to healthcare utilisation and cost may be obtained so that they can be collected in subsequent trials (e.g., changes in medication use, use of NHS resources, etc.). Finally, the feasibility study will enable us to define the cost of further research, thus informing funders’ and the research team’s decisions on whether a full-scale evaluation is worthwhile.

# References

Abate, M., Schiavone, C., Salini, V., Andia, I., 2013. Occurrence of tendon pathologies in metabolic disorders. Rheumatology 52, 599–608. https://doi.org/10.1093/rheumatology/kes395

About osteopathy | Institute of Osteopathy [WWW Document], n.d. URL https://www.iosteopathy.org/about-osteopathy/ (accessed 12.3.21).

Ahmad, I., Verma, S., Noohu, M.M., Shareef, Mohd.Y., Hussain, M.E., 2020. Sensorimotor and gait training improves proprioception, nerve function, and muscular activation in patients with diabetic peripheral neuropathy: a randomized control trial. J Musculoskelet Neuronal Interact 20, 234–248.

Alam, U., Riley, D.R., Jugdey, R.S., Azmi, S., Rajbhandari, S., D’Août, K., Malik, R.A., 2017. Diabetic Neuropathy and Gait: A Review. Diabetes Ther 8, 1253–1264. https://doi.org/10.1007/s13300-017-0295-y

Almurdhi, M.M., Reeves, N.D., Bowling, F.L., Boulton, A.J.M., Jeziorska, M., Malik, R.A., 2016. Reduced Lower-Limb Muscle Strength and Volume in Patients With Type 2 Diabetes in Relation to Neuropathy, Intramuscular Fat, and Vitamin D Levels. Diabetes Care 39, 441–447. https://doi.org/10.2337/dc15-0995

Amato Nesbit, S., Sharma, R., Waldfogel, J.M., Zhang, A., Bennett, W.L., Yeh, H.-C., Chelladurai, Y., Feldman, D., Robinson, K.A., Dy, S.M., 2019. Non-pharmacologic treatments for symptoms of diabetic peripheral neuropathy: a systematic review. Curr Med Res Opin 35, 15–25. https://doi.org/10.1080/03007995.2018.1497958

Andersen, H., 2012. Motor dysfunction in diabetes. Diabetes/Metabolism Research and Reviews 28, 89–92. https://doi.org/10.1002/dmrr.2257

Andersen Hammond, E., Pitz, M., Steinfeld, K., Lambert, P., Shay, B., 2020. An Exploratory Randomized Trial of Physical Therapy for the Treatment of Chemotherapy-Induced Peripheral Neuropathy. Neurorehabil Neural Repair 34, 235–246. https://doi.org/10.1177/1545968319899918

Ashar, Y.K., Gordon, A., Schubiner, H., Uipi, C., Knight, K., Anderson, Z., Carlisle, J., Polisky, L., Geuter, S., Flood, T.F., Kragel, P.A., Dimidjian, S., Lumley, M.A., Wager, T.D., 2021. Effect of Pain Reprocessing Therapy vs Placebo and Usual Care for Patients With Chronic Back Pain: A Randomized Clinical Trial. JAMA Psychiatry. https://doi.org/10.1001/jamapsychiatry.2021.2669

Balk, E.M., Earley, A., Raman, G., Avendano, E.A., Pittas, A.G., Remington, P.L., 2015. Combined Diet and Physical Activity Promotion Programs to Prevent Type 2 Diabetes Among Persons at Increased Risk: A Systematic Review for the Community Preventive Services Task Force. Ann Intern Med 163, 437–451. https://doi.org/10.7326/M15-0452

Ball, E.F., Nur Shafina Muhammad Sharizan, E., Franklin, G., Rogozińska, E., 2017. Does mindfulness meditation improve chronic pain? A systematic review. Current Opinion in Obstetrics and Gynecology 29, 359–366. https://doi.org/10.1097/GCO.0000000000000417

Baskerville, R., Ricci-Cabello, I., Roberts, N., Farmer, A., 2017. Impact of accelerometer and pedometer use on physical activity and glycaemic control in people with Type 2 diabetes: a systematic review and meta-analysis. Diabet Med 34, 612–620. https://doi.org/10.1111/dme.13331

Bialosky, J.E., Beneciuk, J.M., Bishop, M.D., Coronado, R.A., Penza, C.W., Simon, C.B., George, S.Z., 2017. Unraveling the Mechanisms of Manual Therapy: Modeling an Approach. J Orthop Sports Phys Ther 48, 8–18. https://doi.org/10.2519/jospt.2018.7476

Bishop, M.D., Torres-Cueco, R., Gay, C.W., Lluch-Girbés, E., Beneciuk, J.M., Bialosky, J.E., 2015. What effect can manual therapy have on a patient’s pain experience? Pain Management 5, 455–464. https://doi.org/10.2217/pmt.15.39

Bono Mira, M., 2008. EFFECTS OF THE MANIPULATION OF D6 IN THE GLUCEMIA OF DIABETIC PATIENTS [WWW Document].

Çakici, N., Fakkel, T.M., van Neck, J.W., Verhagen, A.P., Coert, J.H., 2016. Systematic review of treatments for diabetic peripheral neuropathy. Diabet Med 33, 1466–1476. https://doi.org/10.1111/dme.13083

Carnes, D., Mars, T., Plunkett, A., Nanke, L., Abbey, H., 2017. A mixed methods evaluation of a third wave cognitive behavioural therapy and osteopathic treatment programme for chronic pain in primary care (OsteoMAP). International Journal of Osteopathic Medicine 24, 12–17. https://doi.org/10.1016/j.ijosm.2017.03.005

Carpenter, M., 2016. can an osteopathic rib-raising technique influence blood glucose levels in type 2 diabetic patients? An ABAB single case research design. [WWW Document].

Castelnuovo, G., Pietrabissa, G., Manzoni, G.M., Cattivelli, R., Rossi, A., Novelli, M., Varallo, G., Molinari, E., 2017. Cognitive behavioral therapy to aid weight loss in obese patients: Current perspectives. Psychology Research and Behavior Management 10. https://doi.org/10.2147/PRBM.S113278

Chan, C.L., Taljaard, M., Lancaster, G.A., Brehaut, J.C., Eldridge, S.M., 2021. Pilot and feasibility studies for pragmatic trials have unique considerations and areas of uncertainty. Journal of Clinical Epidemiology 138, 102–114. https://doi.org/10.1016/j.jclinepi.2021.06.029

Chao, C.-C., Tseng, M.-T., Hsieh, P.-C., Lin, C.-H. (Janice), Huang, S.-L., Hsieh, S.-T., Chiang, M.-C., 2021. Brain Mechanisms of Pain and Dysautonomia in Diabetic Neuropathy: Connectivity Changes in Thalamus and Hypothalamus. The Journal of Clinical Endocrinology & Metabolism. https://doi.org/10.1210/clinem/dgab754

Chatchawan, U., Eungpinichpong, W., Plandee, P., Yamauchi, J., 2015. Effects of Thai Foot Massage on Balance Performance in Diabetic Patients with Peripheral Neuropathy: A Randomized Parallel-Controlled Trial. Med Sci Monit Basic Res 21, 68–75. https://doi.org/10.12659/MSMBR.894163

Colberg, S.R., Sigal, R.J., Yardley, J.E., Riddell, M.C., Dunstan, D.W., Dempsey, P.C., Horton, E.S., Castorino, K., Tate, D.F., 2016. Physical Activity/Exercise and Diabetes: A Position Statement of the American Diabetes Association. Diabetes Care 39, 2065–2079. https://doi.org/10.2337/dc16-1728

Craig, P., Campbell, M., 2015. Evaluability Assessment: a systematic approach to deciding whether and how to evaluate programmes and policies: a What Works Scotland Working paper. https://doi.org/10.13140/RG.2.1.2007.4725

Davies, B., Cramp, F., Gauntlett-Gilbert, J., Wynick, D., McCabe, C.S., 2015. The role of physical activity and psychological coping strategies in the management of painful diabetic neuropathy – A systematic review of the literature. Physiotherapy 101, 319–326. https://doi.org/10.1016/j.physio.2015.04.003

Davies, M., Brophy, S., Williams, R., Taylor, A., 2006. The Prevalence, Severity, and Impact of Painful Diabetic Peripheral Neuropathy in Type 2 Diabetes. Diabetes Care 29, 1518–1522. https://doi.org/10.2337/dc05-2228

Díaz Cerrato, I., 2008. MODIFICACIONES EN LA PIO Y EN LA TA EN DIABÉTICOS TIPO I TRAS LA MANIPULACIÓN GLOBAL OAA SEGÚN FRYETTE.(ECA) [WWW Document].

Dixit, S., Asiri, F., 2014. Pharmacological and nonpharmacological therapies in the management of diabetic peripheral neuropathy in type 2 diabetes: A comprehensive review. Journal of Cardiovascular Disease Research 5.

Eklund, A., Jensen, I., Lohela-Karlsson, M., Hagberg, J., Leboeuf-Yde, C., Kongsted, A., Bodin, L., Axén, I., 2018. The Nordic Maintenance Care program: Effectiveness of chiropractic maintenance care versus symptom-guided treatment for recurrent and persistent low back pain—A pragmatic randomized controlled trial. PLOS ONE 13, e0203029. https://doi.org/10.1371/journal.pone.0203029

Eldridge, S.M., Chan, C.L., Campbell, M.J., Bond, C.M., Hopewell, S., Thabane, L., Lancaster, G.A., 2016a. CONSORT 2010 statement: extension to randomised pilot and feasibility trials. BMJ 355, i5239. https://doi.org/10.1136/bmj.i5239

Eldridge, S.M., Lancaster, G.A., Campbell, M.J., Thabane, L., Hopewell, S., Coleman, C.L., Bond, C.M., 2016b. Defining Feasibility and Pilot Studies in Preparation for Randomised Controlled Trials: Development of a Conceptual Framework. PLoS One 11. https://doi.org/10.1371/journal.pone.0150205

Espelt, A., Arriola, L., Borrell, C., Larranaga, I., Sandin, M., Escolar-Pujolar, A., 2011. Socioeconomic Position and Type 2 Diabetes Mellitus in Europe 1999- 2009: a Panorama of Inequalities. Current Diabetes Reviews 7, 148–158. https://doi.org/10.2174/157339911795843131

Estabrooks, P.A., Glasgow, R.E., Dzewaltowski, D.A., 2003. Physical activity promotion through primary care. JAMA 289, 2913–2916. https://doi.org/10.1001/jama.289.22.2913

Evans, M.C., Wade, C., Hohenschurz-Schmidt, D., Lally, P., Ugwudike, A., Shah, K., Bangerter, N., Sharp, D.J., Rice, A.S.C., 2021. Magnetic Resonance Imaging as a Biomarker in Diabetic and HIV-Associated Peripheral Neuropathy: A Systematic Review-Based Narrative. Frontiers in Neuroscience 15, 1169. https://doi.org/10.3389/fnins.2021.727311

Evans, R.E., Moore, G., Movsisyan, A., Rehfuess, E., 2021. How can we adapt complex population health interventions for new contexts? Progressing debates and research priorities. J Epidemiol Community Health 75, 40–45. https://doi.org/10.1136/jech-2020-214468

Fawkes, C., Carnes, D., 2021. Patient reported outcomes in a large cohort of patients receiving osteopathic care in the United Kingdom. PLOS ONE 16, e0249719. https://doi.org/10.1371/journal.pone.0249719

Fawkes, C., Leach, J., Matias, S., Moore, A., 2009. The standardised data collection project. Standardised data collection within osteopathic practice in the UK: development and first use of a tool to profile osteopathic care in 2010.

Ferini-Strambi, L., 2017. Neuropathic Pain and Sleep: A Review. Pain Ther 6, 19–23. https://doi.org/10.1007/s40122-017-0089-y

Finnerup, N.B., Attal, N., Haroutounian, S., McNicol, E., Baron, R., Dworkin, R.H., Gilron, I., Haanpää, M., Hansson, P., Jensen, T.S., Kamerman, P.R., Lund, K., Moore, A., Raja, S.N., Rice, A.S.C., Rowbotham, M., Sena, E., Siddall, P., Smith, B.H., Wallace, M., 2015. Pharmacotherapy for neuropathic pain in adults: a systematic review and meta-analysis. The Lancet Neurology 14, 162–173. https://doi.org/10.1016/S1474-4422(14)70251-0

Fjeldsoe, B., Neuhaus, M., Winkler, E., Eakin, E., 2011. Systematic review of maintenance of behavior change following physical activity and dietary interventions. Health Psychology 30, 99–109. https://doi.org/10.1037/a0021974

Franssen, W.M.A., Franssen, G.H.L.M., Spaas, J., Solmi, F., Eijnde, B.O., 2020. Can consumer wearable activity tracker-based interventions improve physical activity and cardiometabolic health in patients with chronic diseases? A systematic review and meta-analysis of randomised controlled trials. Int J Behav Nutr Phys Act 17, 57. https://doi.org/10.1186/s12966-020-00955-2

Freedland, K.E., King, A.C., Ambrosius, W.T., Mayo-Wilson, E., Mohr, D.C., Czajkowski, S.M., Thabane, L., Collins, L.M., Rebok, G.W., Treweek, S.P., Cook, T.D., Edinger, J.D., Stoney, C.M., Campo, R.A., Young-Hyman, D., Riley, W.T., 2019. The selection of comparators for randomized controlled trials of health-related behavioral interventions: recommendations of an NIH expert panel. Journal of Clinical Epidemiology 110, 74–81. https://doi.org/10.1016/j.jclinepi.2019.02.011

Fu, Q., Yang, H., Zhang, L., Liu, Yang, Li, X., Dai, M., Yang, Y., Yang, S., Xie, Y., Liu, Ying, Fu, L., Liu, Z., Zhang, Q., 2020. Traditional Chinese medicine foot bath combined with acupoint massage for the treatment of diabetic peripheral neuropathy: A systematic review and meta-analysis of 31 RCTs. Diabetes/Metabolism Research and Reviews 36, e3218. https://doi.org/10.1002/dmrr.3218

General Osteopathic Council, 2018. Standards of practice [WWW Document]. URL https://www.osteopathy.org.uk/standards/osteopathic-practice/ (accessed 4.20.20).

Gilron, I., Baron, R., Jensen, T., 2015. Neuropathic Pain: Principles of Diagnosis and Treatment. Mayo Clinic Proceedings 90, 532–545. https://doi.org/10.1016/j.mayocp.2015.01.018

Godfrey, E., Holmes, M.G., Wileman, V., McCracken, L., Norton, S., Moss-Morris, R., Pallet, J., Sanders, D., Barcellona, M., Critchley, D., 2016. Physiotherapy informed by Acceptance and Commitment Therapy (PACT): protocol for a randomised controlled trial of PACT versus usual physiotherapy care for adults with chronic low back pain. BMJ Open 6, e011548. https://doi.org/10.1136/bmjopen-2016-011548

Godfrey, E., Wileman, V., Galea Holmes, M., McCracken, L.M., Norton, S., Moss-Morris, R., Noonan, S., Barcellona, M., Critchley, D., 2020. Physical Therapy Informed by Acceptance and Commitment Therapy (PACT) Versus Usual Care Physical Therapy for Adults With Chronic Low Back Pain: A Randomized Controlled Trial. The Journal of Pain 21, 71–81. https://doi.org/10.1016/j.jpain.2019.05.012

Grewal, G.S., Schwenk, M., Lee-Eng, J., Parvaneh, S., Bharara, M., Menzies, R.A., Talal, T.K., Armstrong, D.G., Najafi, B., 2015. Sensor-Based Interactive Balance Training with Visual Joint Movement Feedback for Improving Postural Stability in Diabetics with Peripheral Neuropathy: A Randomized Controlled Trial. GER 61, 567–574. https://doi.org/10.1159/000371846

Hall, K.T., Loscalzo, J., 2019. Drug-Placebo Additivity in Randomized Clinical Trials. Clinical Pharmacology & Therapeutics 106, 1191–1197. https://doi.org/10.1002/cpt.1626

Hamid, N., 2011. Effects of stress management training on glycemic control in women with type 2 diabetes. Iranian Journal of Endocrinology and Metabolism 13, 346–353.

Hart, A.J.P., Beckley, K., 2015. 5. Acceptance and Commitment Therapy, in: Formulation in ActionApplying Psychological Theory to Clinical Practice. De Gruyter, Berlin, Boston. https://doi.org/10.1515/9783110471014-007

Harvey, E., Burton, A.K., Moffett, J.K., Breen, A., 2003. Spinal manipulation for low-back pain: a treatment package agreed by the UK chiropractic, osteopathy and physiotherapy professional associations. Manual Therapy 8, 46–51. https://doi.org/10.1054/math.2002.0472

He, X., Li, J., Wang, B., Yao, Q., Li, L., Song, R., Shi, X., Zhang, J., 2017. Diabetes self-management education reduces risk of all-cause mortality in type 2 diabetes patients: a systematic review and meta-analysis. Endocrine 55, 712–731. https://doi.org/10.1007/s12020-016-1168-2

Hernández-Secorún, M., Vidal-Peracho, C., Márquez-Gonzalvo, S., Corral-de-Toro, J., Müller-Thyssen-Uriarte, J., Rodríguez-Sanz, J., Lucha-López, M.O., Tricás-Moreno, J.M., Hidalgo-García, C., 2021. Exercise and Manual Therapy for Diabetic Peripheral Neuropathy: A Systematic Review. Applied Sciences 11, 5665. https://doi.org/10.3390/app11125665

Hewston, P., Deshpande, N., 2018. Fear of Falling and Balance Confidence in Older Adults With Type 2 Diabetes Mellitus: A Scoping Review. Canadian Journal of Diabetes 42, 664–670. https://doi.org/10.1016/j.jcjd.2018.02.009

Hill-Briggs, F., Adler, N.E., Berkowitz, S.A., Chin, M.H., Gary-Webb, T.L., Navas-Acien, A., Thornton, P.L., Haire-Joshu, D., 2021. Social Determinants of Health and Diabetes: A Scientific Review. Diabetes Care 44, 258–279. https://doi.org/10.2337/dci20-0053

Hodkinson, A., Kontopantelis, E., Adeniji, C., van Marwijk, H., McMillian, B., Bower, P., Panagioti, M., 2021. Interventions Using Wearable Physical Activity Trackers Among Adults With Cardiometabolic Conditions: A Systematic Review and Meta-analysis. JAMA Netw Open 4, e2116382. https://doi.org/10.1001/jamanetworkopen.2021.16382

International Diabetes Federation, 2019. IDF Diabetes Atlas 9th edition [WWW Document]. URL https://diabetesatlas.org/en/ (accessed 9.8.21).

Izgu, N., Gok Metin, Z., Karadas, C., Ozdemir, L., Metinarikan, N., Corapcıoglu, D., 2020. Progressive Muscle Relaxation and Mindfulness Meditation on Neuropathic Pain, Fatigue, and Quality of Life in Patients With Type 2 Diabetes: A Randomized Clinical Trial. Journal of Nursing Scholarship 52, 476–487. https://doi.org/10.1111/jnu.12580

Jackson, W., Zale, E.L., Berman, S.J., Malacarne, A., Lapidow, A., Schatman, M.E., Kulich, R., Vranceanu, A.-M., 2019. Physical functioning and mindfulness skills training in chronic pain: a systematic review. J Pain Res 12, 179–189. https://doi.org/10.2147/JPR.S172733

Javed, S., Petropoulos, I.N., Alam, U., Malik, R.A., 2015. Treatment of painful diabetic neuropathy. Therapeutic Advances in Chronic Disease 6, 15–28. https://doi.org/10.1177/2040622314552071

Karran, E.L., Grant, A.R., Moseley, G.L., 2020. Low back pain and the social determinants of health: a systematic review and narrative synthesis. PAIN 161, 2476–2493. https://doi.org/10.1097/j.pain.0000000000001944

Kec, D., Rajdova, A., Raputova, J., Adamova, B., Srotova, I., Nekvapilova, E.K., Michalcakova, R.N., Horakova, M., Belobradkova, J., Olsovsky, J., Weber, P., Hajas, G., Kaiserova, M., Mazanec, R., Potockova, V., Ehler, E., Forgac, M., Birklein, F., Üçeyler, N., Sommer, C., Bednarik, J., Vlckova, E., 2021. Risk factors for depression and anxiety in painful and painless diabetic polyneuropathy: A multicentre observational cross-sectional study. European Journal of Pain n/a. https://doi.org/10.1002/ejp.1865

Keefe, F.J., Main, C.J., George, S.Z., 2018. Advancing Psychologically Informed Practice for Patients With Persistent Musculoskeletal Pain: Promise, Pitfalls, and Solutions. Phys Ther 98, 398–407. https://doi.org/10.1093/ptj/pzy024

Kerr, F., Wiechula, R., Feo, R., Schultz, T., Kitson, A., 2019. Neurophysiology of human touch and eye gaze in therapeutic relationships and healing: a scoping review. JBI Database System Rev Implement Rep 17, 209–247. https://doi.org/10.11124/JBISRIR-2017-003549

Khan, K.S., Andersen, H., 2022. The Impact of Diabetic Neuropathy on Activities of Daily Living, Postural Balance and Risk of Falls - A Systematic Review. J Diabetes Sci Technol 16, 289–294. https://doi.org/10.1177/1932296821997921

Kiegerl, G., 2007. Does Osteopathy Influence Diabetes Mellitus Type II? [WWW Document].

King, H.H., 2016. Manual Therapy Shown To Improve Diabetic Foot Ulcer Healing. Journal of Osteopathic Medicine 116, 685–686. https://doi.org/10.7556/jaoa.2016.135

Kioskli, K., Scott, W., Winkley, K., Godfrey, E., McCracken, L., 2020. Online Acceptance and Commitment Therapy for people with painful diabetic neuropathy in the United Kingdom: A single-arm feasibility trial. Pain Med 1–35.

Kioskli, K., Scott, W., Winkley, K., Kylakos, S., McCracken, L.M., 2019a. Psychosocial Factors in Painful Diabetic Neuropathy: A Systematic Review of Treatment Trials and Survey Studies. Pain Medicine 20, 1756–1773. https://doi.org/10.1093/pm/pnz071

Kioskli, K., Winkley, K., McCracken, L.M., 2019b. Might psychological flexibility processes and Acceptance and Commitment Therapy (ACT) apply in adults with painful diabetic neuropathy? A cross-sectional survey. Journal of Contextual Behavioral Science 13, 66–73. https://doi.org/10.1016/j.jcbs.2019.07.002

Kuhmann, O., 2008. The impact of osteopathic treatment on intraocular hypertension – an experimental study [WWW Document].

Lau, M.A., McMain, S.F., 2005. Integrating Mindfulness Meditation with Cognitive and Behavioural Therapies: The Challenge of Combining Acceptance- and Change-Based Strategies. Can J Psychiatry 50, 863–869. https://doi.org/10.1177/070674370505001310

Liampas, A., Rekatsina, M., Vadalouca, A., Paladini, A., Varrassi, G., Zis, P., 2020. Non-Pharmacological Management of Painful Peripheral Neuropathies: A Systematic Review. Adv Ther 37, 4096–4106. https://doi.org/10.1007/s12325-020-01462-3

Licciardone, J.C., Kearns, C.M., Hodge, L.M., Minotti, D.E., 2013. Osteopathic Manual Treatment in Patients With Diabetes Mellitus and Comorbid Chronic Low Back Pain: Subgroup Results From the OSTEOPATHIC Trial. Journal of Osteopathic Medicine 113, 468–478. https://doi.org/10.7556/jaoa.2013.113.6.468

Lin, T., Huang, F., Zhao, S., Qiu, M., Wen, J., Liu, M., 2021. Acupuncture for diabetic peripheral neuropathy: An overview of systematic reviews. Complementary Therapies in Clinical Practice 43, 101375. https://doi.org/10.1016/j.ctcp.2021.101375

Maggard-Gibbons, M., Maglione, M., Livhits, M., Ewing, B., Maher, A.R., Hu, J., Li, Z., Shekelle, P.G., 2013. Bariatric Surgery for Weight Loss and Glycemic Control in Nonmorbidly Obese Adults With Diabetes: A Systematic Review. JAMA 309, 2250–2261. https://doi.org/10.1001/jama.2013.4851

Marmot, M., 2020. Health equity in England: the Marmot review 10 years on. BMJ 368. https://doi.org/10.1136/bmj.m693

McClintock, A.S., McCarrick, S.M., Garland, E.L., Zeidan, F., Zgierska, A.E., 2019. Brief Mindfulness-Based Interventions for Acute and Chronic Pain: A Systematic Review. The Journal of Alternative and Complementary Medicine 25, 265–278. https://doi.org/10.1089/acm.2018.0351

McCracken, L.M., Morley, S., 2014. The Psychological Flexibility Model: A Basis for Integration and Progress in Psychological Approaches to Chronic Pain Management. The Journal of Pain 15, 221–234. https://doi.org/10.1016/j.jpain.2013.10.014

McCracken, L.M., Yu, L., Vowles, K.E., 2022. New generation psychological treatments in chronic pain. BMJ 376, e057212. https://doi.org/10.1136/bmj-2021-057212

McGuire, H., Longson, D., Adler, A., Farmer, A., Lewin, I., 2016. Management of type 2 diabetes in adults: summary of updated NICE guidance. BMJ 353, i1575. https://doi.org/10.1136/bmj.i1575

McGuirk, J., 2012. The place of touch in Counselling and Psychotherapy and the potential for healing within the therapeutic relationship. Inside Out 68.

Meisinger, C., Bongaerts, B.W.C., Heier, M., Amann, U., Kowall, B., Herder, C., Rückert-Eheberg, I.-M., Rathmann, W., Ziegler, D., 2018. Neuropathic pain is not adequately treated in the older general population: Results from the KORA F4 survey. Pharmacoepidemiol Drug Saf 27, 806–814. https://doi.org/10.1002/pds.4559

Moisset, X., Bouhassira, D., Avez Couturier, J., Alchaar, H., Conradi, S., Delmotte, M.H., Lanteri-Minet, M., Lefaucheur, J.P., Mick, G., Piano, V., Pickering, G., Piquet, E., Regis, C., Salvat, E., Attal, N., 2020. Pharmacological and non-pharmacological treatments for neuropathic pain: Systematic review and French recommendations. Rev Neurol (Paris) 176, 325–352. https://doi.org/10.1016/j.neurol.2020.01.361

Molsted, S., Tribler, J., Snorgaard, O., 2012. Musculoskeletal pain in patients with type 2 diabetes. Diabetes Research and Clinical Practice 96, 135–140. https://doi.org/10.1016/j.diabres.2011.12.022

Naranjo, C., Del Reguero, L., Moratalla, G., Hercberg, M., Valenzuela, M., Failde, I., 2019. Anxiety, depression and sleep disorders in patients with diabetic neuropathic pain: a systematic review. Expert Review of Neurotherapeutics 19, 1201–1209. https://doi.org/10.1080/14737175.2019.1653760

Nathan, H.J., Poulin, P., Wozny, D., Taljaard, M., Smyth, C., Gilron, I., Sorisky, A., Lochnan, H., Shergill, Y., 2017. Randomized Trial of the Effect of Mindfulness-Based Stress Reduction on Pain-Related Disability, Pain Intensity, Health-Related Quality of Life, and A1C in Patients With Painful Diabetic Peripheral Neuropathy. Clinical Diabetes 35, 294–304. https://doi.org/10.2337/cd17-0077

NICE, 2015. Overview | Type 2 diabetes in adults: management | Guidance | NICE [WWW Document]. URL https://www.nice.org.uk/guidance/ng28 (accessed 9.8.21).

Onifer, S.M., Sozio, R.S., DiCarlo, D.M., Li, Q., Donahue, R.R., Taylor, B.K., Long, C.R., 2018. Spinal Manipulative Therapy Reduces Peripheral Neuropathic Pain in the Rat. Neuroreport 29, 191–196. https://doi.org/10.1097/WNR.0000000000000949

Pandey, A., Tripathi, P., Pandey, R., Srivatava, R., Goswami, S., 2011. Alternative therapies useful in the management of diabetes: A systematic review. J Pharm Bioallied Sci 3, 504–512. https://doi.org/10.4103/0975-7406.90103

Parasoglou, P., Rao, S., Slade, J.M., 2017. Declining Skeletal Muscle Function in Diabetic Peripheral Neuropathy. Clinical Therapeutics 39, 1085–1103. https://doi.org/10.1016/j.clinthera.2017.05.001

Pêgas de Oliveira, A., 2008. VERIFICATION OF THE ALTERATIONS OF GLUCEMIA AND INSULINEMIA IN PATIENTS WITH DIABETES TYPE II SUBMISSIVE TO A PROTOCOL OF OSTEOPATHIC TREATMENT [WWW Document].

Plunkett, A., Fawkes, C., Carnes, D., 2021. UK osteopathic practice in 2019: a retrospective analysis of practice data. https://doi.org/10.1101/2021.01.28.21250601

Racaru, S., Sturt, J., Celik, A., 2021. The Effects of Psychological Interventions on Diabetic Peripheral Neuropathy: A Systematic Review and Meta-Analysis. Pain Management Nursing 22, 302–311. https://doi.org/10.1016/j.pmn.2020.11.001

Raveendran, A.V., Chacko, E.C., Pappachan, J.M., 2018. Non-pharmacological Treatment Options in the Management of Diabetes Mellitus. Eur Endocrinol 14, 31–39. https://doi.org/10.17925/EE.2018.14.2.31

Rossen, J., Larsson, K., Hagströmer, M., Yngve, A., Brismar, K., Ainsworth, B., Åberg, L., Johansson, U.-B., 2021. Effects of a three-armed randomised controlled trial using self-monitoring of daily steps with and without counselling in prediabetes and type 2 diabetes-the Sophia Step Study. Int J Behav Nutr Phys Act 18, 121. https://doi.org/10.1186/s12966-021-01193-w

Safren, S.A., Gonzalez, J.S., Wexler, D.J., Psaros, C., Delahanty, L.M., Blashill, A.J., Margolina, A.I., Cagliero, E., 2014. A Randomized Controlled Trial of Cognitive Behavioral Therapy for Adherence and Depression (CBT-AD) in Patients With Uncontrolled Type 2 Diabetes. Diabetes Care 37, 625–633. https://doi.org/10.2337/dc13-0816

Said, G., 2007. Diabetic neuropathy—a review. Nat Rev Neurol 3, 331–340. https://doi.org/10.1038/ncpneuro0504

Scott, W., Badenoch, J., Solar, M.G.C.M. del, Brown, D.A., Kemp, H., McCracken, L., Williams, A.C. de C., Rice, A.S., 2020. Acceptability of psychologically-based pain management and online delivery for people living with HIV and chronic neuropathic pain: a qualitative study. Scandinavian Journal of Pain.

Scott, W., Guildford, B.J., Badenoch, J., Driscoll, E., Chilcot, J., Norton, S., Kemp, H.I., Lee, M.J., Lwanga, J., Boffito, M., Moyle, G., Post, F.A., Campbell, L., Josh, J., Clift, P., Williams, A.C. de C., Rice, A., McCracken, L.M., 2021. Feasibility Randomized-Controlled Trial of Online Acceptance and Commitment Therapy for Painful Peripheral Neuropathy in People Living with HIV: The OPEN Study. Eur J Pain 25, 1493–1507. https://doi.org/10.1002/ejp.1762

Scott, W., Hann, K.E.J., McCracken, L.M., 2016. A Comprehensive Examination of Changes in Psychological Flexibility Following Acceptance and Commitment Therapy for Chronic Pain. J Contemp Psychother 46, 139–148. https://doi.org/10.1007/s10879-016-9328-5

Shafer, D., Gooing, J., Lee, V., Seffinger, M.A., 2020. Musculoskeletal Conditions in Patients With Diabetes: A Narrative Review. Journal of Osteopathic Medicine 120, 660–664. https://doi.org/10.7556/jaoa.2020.123

Singh, S.B., Singh, P.P., Singh, S., Aggarwal, R., Singh, J., 2012. Effect of Nerve Mobilization on Vibration Perception Threshold in Diabetic Peripheral Neuropathy. Indian Journal of Physiotherapy & Occupational Therapy-An International Journal 6, 189–195.

Skivington, K., Matthews, L., Simpson, S.A., Craig, P., Baird, J., Blazeby, J.M., Boyd, K.A., Craig, N., French, D.P., McIntosh, E., Petticrew, M., Rycroft-Malone, J., White, M., Moore, L., 2021. A new framework for developing and evaluating complex interventions: update of Medical Research Council guidance. BMJ 374, n2061. https://doi.org/10.1136/bmj.n2061

Sobhani, S., Asayesh, H., Sharifi, F., Djalalinia, S., Baradaran, H.R., Arzaghi, S.M., Mansourian, M., Rezapoor, A., Ansari, H., Masoud, M.P., 2014. Prevalence of diabetic peripheral neuropathy in Iran: a systematic review and meta-analysis. Journal of Diabetes & Metabolic Disorders 13, 1–7.

Song, X.-J., Huang, Z.-J., Song, W.B., Song, X.-S., Fuhr, A.F., Rosner, A.L., Ndtan, H., Rupert, R.L., 2016. Attenuation Effect of Spinal Manipulation on Neuropathic and Postoperative Pain Through Activating Endogenous Anti-Inflammatory Cytokine Interleukin 10 in Rat Spinal Cord. Journal of Manipulative and Physiological Therapeutics 39, 42–53. https://doi.org/10.1016/j.jmpt.2015.12.004

Sözen, T., Başaran, N.Ç., Tınazlı, M., Özışık, L., 2018. Musculoskeletal problems in diabetes mellitus. Eur J Rheumatol 5, 258–265. https://doi.org/10.5152/eurjrheum.2018.18044

Staniszewska, S., Brett, J., Simera, I., Seers, K., Mockford, C., Goodlad, S., Altman, D.G., Moher, D., Barber, R., Denegri, S., Entwistle, A., Littlejohns, P., Morris, C., Suleman, R., Thomas, V., Tysall, C., 2017. GRIPP2 reporting checklists: tools to improve reporting of patient and public involvement in research. BMJ 358, j3453. https://doi.org/10.1136/bmj.j3453

Tan, T., Barry, P., Reken, S., Baker, M., 2010. Pharmacological management of neuropathic pain in non-specialist settings: summary of NICE guidance. BMJ 340, c1079. https://doi.org/10.1136/bmj.c1079

Teixeira, E., 2010. The Effect of Mindfulness Meditation on Painful Diabetic Peripheral Neuropathy in Adults Older Than 50 Years. Holistic Nursing Practice 24, 277–283. https://doi.org/10.1097/HNP.0b013e3181f1add2

Themistocleous, A.C., Ramirez, J.D., Shillo, P.R., Lees, J.G., Selvarajah, D., Orengo, C., Tesfaye, S., Rice, A.S.C., Bennett, D.L.H., 2016. The Pain in Neuropathy Study (PiNS): a cross-sectional observational study determining the somatosensory phenotype of painful and painless diabetic neuropathy. Pain 157, 1132–1145. https://doi.org/10.1097/j.pain.0000000000000491

Tölle, T., Xu, X., Sadosky, A.B., 2006. Painful diabetic neuropathy: a cross-sectional survey of health state impairment and treatment patterns. Journal of Diabetes and its Complications 20, 26–33. https://doi.org/10.1016/j.jdiacomp.2005.09.007

Touma, C., Pannain, S., 2011. Does lack of sleep cause diabetes? Cleve Clin J Med 78, 549–558. https://doi.org/10.3949/ccjm.78a.10165

Underwood, M.R., Morton, V., Farrin, A., on behalf of the UK BEAM trial team, 2007. Do baseline characteristics predict response to treatment for low back pain? Secondary analysis of the UK BEAM dataset [ISRCTN32683578]. Rheumatology 46, 1297–1302. https://doi.org/10.1093/rheumatology/kem113

Vermeersch, A., Quaghebeur, J., 2018. The effect of an osteopathic treatment on the blood glucose level in diabetic patients. [WWW Document].

Wagner, J.A., Bermudez-Millan, A., Damio, G., Segura-Perez, S., Chhabra, J., Vergara, C., Feinn, R., Perez-Escamilla, R., 2016. A randomized, controlled trial of a stress management intervention for Latinos with type 2 diabetes delivered by community health workers: Outcomes for psychological wellbeing, glycemic control, and cortisol. Diabetes Research and Clinical Practice 120, 162–170. https://doi.org/10.1016/j.diabres.2016.07.022

Watson, J.A., Ryan, C.G., Cooper, L., Ellington, D., Whittle, R., Lavender, M., Dixon, J., Atkinson, G., Cooper, K., Martin, D.J., 2019. Pain Neuroscience Education for Adults With Chronic Musculoskeletal Pain: A Mixed-Methods Systematic Review and Meta-Analysis. J Pain 20, P1140.e1-1140.e2. https://doi.org/10.1016/j.jpain.2019.02.011

Whicher, C.A., O’Neill, S., Holt, R.I.G., 2020. Diabetes in the UK: 2019. Diabetic Medicine 37, 242–247. https://doi.org/10.1111/dme.14225

Williams, A.C. de C., Fisher, E., Hearn, L., Eccleston, C., 2020. Psychological therapies for the management of chronic pain (excluding headache) in adults. Cochrane Database of Systematic Reviews. https://doi.org/10.1002/14651858.CD007407.pub4

Yamamoto, J.M., Kellett, J.E., Balsells, M., García-Patterson, A., Hadar, E., Solà, I., Gich, I., Beek, E.M. van der, Castañeda-Gutiérrez, E., Heinonen, S., Hod, M., Laitinen, K., Olsen, S.F., Poston, L., Rueda, R., Rust, P., Lieshout, L. van, Schelkle, B., Murphy, H.R., Corcoy, R., 2018. Gestational Diabetes Mellitus and Diet: A Systematic Review and Meta-analysis of Randomized Controlled Trials Examining the Impact of Modified Dietary Interventions on Maternal Glucose Control and Neonatal Birth Weight. Diabetes Care 41, 1346–1361. https://doi.org/10.2337/dc18-0102

Ziegler, D., Tesfaye, S., Spallone, V., Gurieva, I., Al Kaabi, J., Mankovsky, B., Martinka, E., Radulian, G., Thy Nguyen, K., Stirban, A.O., Tankova, T., Varkonyi, T., Freeman, R., Kempler, P., Jm Boulton, A., 2021. Screening, diagnosis and management of diabetic sensorimotor polyneuropathy in clinical practice: International expert consensus recommendations. Diabetes Res Clin Pract 109063. https://doi.org/10.1016/j.diabres.2021.109063
