# Supplementary material for: A multimodal intervention of manual therapy, exercise, and psychological management for painful diabetic neuropathy: intervention development and feasibility trial protocol
Source: Pain Manag. 2025 Jun 11;15(7):387–99. doi: 10.1080/17581869.2025.2515010 (PMC12218422; doi:10.1080/17581869.2025.2515010)
Supplement: Supplemental Material [file IPMT_A_2515010_SM6913.zip › suppl_data/S4 TIDieR-Placebo_CoPPS_hybrid_NeuOst_feasibility_trial.docx]

**Completed for the NeuOst feasibility trial: TIDieR-Placebo Checklist with added items from the Recommendations for the Development, Implementation, and Reporting of Control Interventions in Efficacy Trials of Physical, Psychological, and Self-Management Therapies (CoPPS) statement.**

Note: In the below table, Appendix 1 refers to the document ‘Development and description of the NeuOst intervention’, and Appendix 2 refers to the document ‘Development and description of the control intervention’.

| **Item** | **Where located** | |  | **Where located** | |
| --- | --- | --- | --- | --- | --- |
|  | **Primary paper (page or appendix number)** | **Other (details)** |  | **Primary paper (page or appendix number)** | **Other (details)** |
| **Active intervention** |  |  | **Control intervention** |  |  |
| ***0 Processes of sham intervention development*** |  |  |  |  | For all items, also see Appendix 2 of RCT protocol publication |
|  |  |  | *Sources and processes that informed the development of the control intervention.* | Results (section “Control intervention”) | Appendix 2 (section “Control intervention rationale and development”) |
| **1 Brief Name** |  |  |  |  |  |
| Provide the name or a phrase that describes the intervention | Abstract and Introduction | For all items, also see Appendix 1 of RCT protocol publication | Provide the name or a phrase that describes the control intervention | Abstract and Results (“study objectives”) |  |
| **2 Why** |  |  |  |  |  |
| Describe any rationale, theory, or goal of the elements essential to the intervention | Results (“The NeuOst intervention”) | Appendix 1 (section “Intervention theory”) | Describe any rationale, theory, or goal of the elements essential to the control intervention*  *Theoretical considerations underlying the control intervention (including explicit mechanistic rationales and objectives of the control intervention)* | Results (“Interventions - Control intervention”) | Appendix 2 (sections “Control intervention rationale and development” and “detailed description of control intervention”) |
| **3 What (materials)** |  |  |  |  |  |
|  |  |  | *A highly detailed description of the content of the control intervention (covering all components listed in table 2 of the CoPPS publication and including resemblance or differences to the test intervention)* | - | Appendix 2 (section “Detailed description of control intervention” and control intervention manual) |
| Describe any physical or informational materials used in the intervention, including those provided to participants or used in intervention delivery or in training of intervention providers. Provide information on where the materials can be accessed (such as online appendix, URL) | - | Appendix 1 (“Elaboration of individual programme components”) | Describe any physical or informational materials used in the control intervention, including those provided to participants or used in intervention delivery or in training of intervention providers. Provide information on where the materials can be accessed (such as an online appendix, URL) | - | Appendix 1 (“Elaboration of individual programme components”), and Appendix 2 (“Detailed description of control intervention”) |
| **4 What (procedures)** |  |  |  |  |  |
| Describe each of the procedures, activities, and/or processes used in the intervention, including any enabling or support activities | Results (sections “The NeuOst intervention” and “Interventions - Tested intervention (NeuOst treatment)”) | Appendix 1 (“Elaboration of individual programme components”) | Describe each of the procedures, activities, and/or processes used in the control intervention, including any enabling or support activities | Results (“Interventions - Control intervention”) | Appendix 1 (“Elaboration of individual programme components”), and Appendix 2 (“Detailed description of control intervention |
| **5 Who provided** |  |  |  |  |  |
| For each category of intervention provider (such as psychologist, nursing assistant), describe their expertise, background, and any specific training given  *Should also include a description of provider behaviour, verbal and non-verbal communication, and issues of equipoise as detailed in the text of the CoPPS guideline; as well as means to control these provider-related factors.* | Results (“Trial provider training and eligibility”)  Demographic information of trial providers not reported due to the protocol nature of the present publication. | Appendices 1 and 2 (sections “provider training course” and “providers and provider training”) | For each category of control intervention provider (such as psychologist, nursing assistant), describe their expertise, background, and any specific training given  *Should also include a description of provider behaviour, verbal and non-verbal communication, and issues of equipoise as detailed in the text of the CoPPS guideline; as well as means to control these provider-related factors.* | Results (“Trial provider training and eligibility”) | Appendices 1 and 2 (sections “provider training course” and “providers and provider training”) |
| *Additional provider-related information*  *Reporting should further include how issues of equipoise and provider expectancy were addressed; and if and how provider behaviour and verbal and non-verbal communication were controlled in each group. If different sets of providers were employed to report test and control interventions, this needs to be reported along with differences in their characteristics.* | - | Appendices 1 and 2 (sections “provider training course” and “providers and provider training”) | *Additional provider-related information*  *Reporting should further include how issues of equipoise and provider expectancy were addressed; and if and how provider behaviour and verbal and non-verbal communication were controlled in each group. If different sets of providers were employed to report test and control interventions, this needs to be reported along with differences in their characteristics.* | - | Appendices 1 and 2 (sections “provider training course” and “providers and provider training”) |
| **6 How** |  |  |  |  |  |
| Describe the modes of delivery (such as face to face or by some other mechanism, such as internet or telephone) of the intervention and whether it was provided individually or in a group | Results (sections “The NeuOst intervention” and “Interventions - Tested intervention (NeuOst treatment)”) | Appendix 1 (“Intervention theory” and “Elaboration of programme components” sections) | Describe the modes of delivery (such as face to face or by some other mechanism, such as internet or telephone) of the control intervention and whether it was provided individually or in a group | Results (“Interventions - Control intervention”) | Appendix 2 (section “Detailed description of control intervention”, and “Matched components”) |
| **7 Where** |  |  |  |  |  |
| Describe the type(s) of location(s) where the intervention occurred, including any necessary infrastructure or relevant features | Results: (“Study setting”) | - | Describe the type(s) of locations(s) and settings where the control intervention occurred, including any necessary infrastructure or relevant features | Results: (“Study setting”) | - |
| **8 When and how much** |  |  |  |  |  |
| Describe the number of times the intervention was delivered and over what period of time including the number of sessions, their schedule, and their duration, intensity, or dose | Results: (“Interventions”) | Appendix 1 (“Intervention theory”, “Elaboration of programme components”, and “Treatment protocol for feasibility trial” sections) | Describe the number of times the control intervention was delivered and over what period of time including the number of sessions, their schedule, and their duration, intensity, or dose. If relevant, include the duration of the pre-, and post-randomisation consultations | Results: (“Interventions”) | Appendix 2 (“Control intervention protocol per trial session”) |
| **9 Tailoring** |  |  |  |  |  |
| If the intervention was planned to be personalised, titrated or adapted, then describe what, why, when, and how | Results (“The NeuOst intervention”) | Appendix 1 (“Treatment protocol for feasibility trial”) | If the control intervention was planned to be personalised, titrated or adapted, then describe what, why, when, and how | - | Appendix 2 (“Detailed description of control intervention”, “Matched components” and “Control intervention protocol per trial session”) |
| **10 Modifications** |  |  |  |  |  |
| If the intervention was modified during the course of the study, describe the changes (what, why, when, and how) | N/A (due to protocol nature of publication) | - | If the control intervention was modified during the course of the study, describe the changes (what, why, when, and how) | N/A (due to protocol nature of publication) | - |
| **11 How well: planned** |  |  |  |  |  |
| Planned: If intervention adherence or fidelity was assessed, describe how and by whom, and if any strategies were used to maintain or improve fidelity, describe them | Results “Primary (feasibility) outcomes”) | Full protocol (Open Science Framework, osf.io/jyftb) | Planned: If control intervention adherence or fidelity was assessed, describe how and by whom, and if any strategies were used to maintain or improve fidelity, describe them | Results “Primary (feasibility) outcomes”) | Full protocol (Open Science Framework, osf.io/jyftb) |
| **12 How well: actual** |  |  |  |  |  |
| Actual: If intervention adherence or fidelity was assessed, describe the extent to which the intervention was delivered as planned | N/A (due to protocol nature of publication) | - | Actual: If control intervention adherence or fidelity was assessed, describe the extent to which the intervention was delivered as planned | N/A (due to protocol nature of publication) | - |
|  |  |  | *Whether any reasons for loss to follow-up (participant attrition) during the trial were related to the control intervention.* | N/A (due to protocol nature of publication) | - |
| **13 Measuring the success of blinding** |  |  |  |  |  |
| Was blinding measured, and if so: how, and what were the results of such measurement?  *Blinding should always be assessed if it was an objective of the control intervention, and that results should be reported as summary statistics per group, allowing independent calculation of blinding indices.* | Results (“Primary (feasibility) outcomes”) | Full protocol (Open Science Framework, osf.io/jyftb) | *Blinding should always be assessed if it was an objective of the control intervention, and that results should be reported as summary statistics per group, allowing independent calculation of blinding indices.* | Results (“Primary (feasibility) outcomes”) | Full protocol (Open Science Framework, osf.io/jyftb) |
| ***14 Assessment of participant expectation*** |  |  |  |  |  |
| *Report the method of assessment, timepoints, and results as summary statistics per group.* | Results (“Exploratory outcomes”) | Full protocol (Open Science Framework, osf.io/jyftb) | *Report the method of assessment, timepoints, and results as summary statistics per group.* | Results (“Exploratory outcomes”) | Full protocol (Open Science Framework, osf.io/jyftb) |
