# Supplementary material for: A multimodal intervention of manual therapy, exercise, and psychological management for painful diabetic neuropathy: intervention development and feasibility trial protocol
Source: Pain Manag. 2025 Jun 11;15(7):387–99. doi: 10.1080/17581869.2025.2515010 (PMC12218422; doi:10.1080/17581869.2025.2515010)
Supplement: Supplemental Material [file IPMT_A_2515010_SM6913.zip › suppl_data/S5 Trial_Recruitment_Flyer_NeuOst_Nov_2024.pdf]

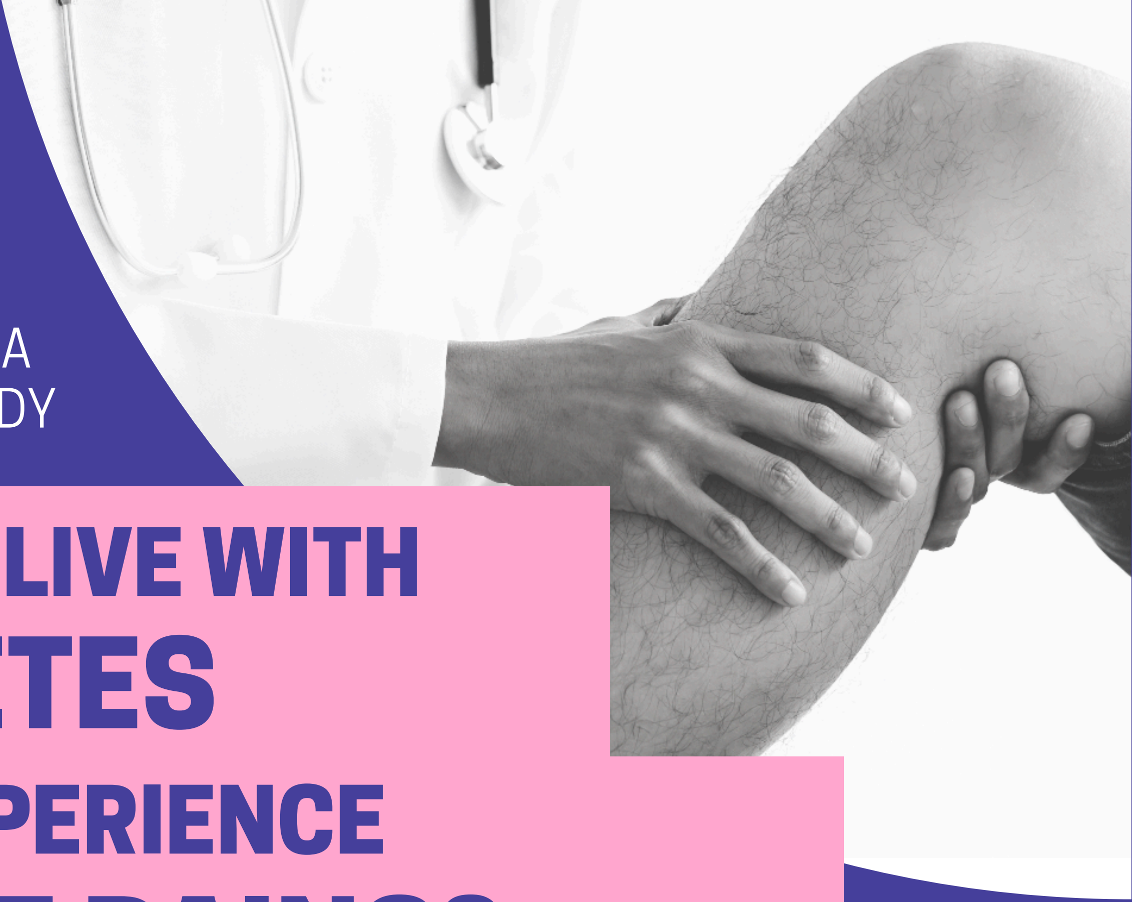

LOOKING FOR PATIENTS
TO TAKE PART IN A TREATMENT STUDY

DO YOU LIVE WITH DIABETES AND EXPERIENCE NERVE PAINS?

Help us test a new drug-free approach for people like yourself!

Are you looking for

RELIEF FOR DIABETIC NEUROPATHY?

Get involved in a clinical trial.

- Opportunity for 5 free treatments
- 4 months duration

We reimburse costs and pay for your time.

Find out more on the back of the flyer or at www.uco.ac.uk/NeuOst

Imperial College London

NeuOst

HSU HEALTH SCIENCES UNIVERSITY

WHAT'S THIS ABOUT?

If you live with painful diabetic neuropathy or diabetes-related foot pain, we invite you to be part of our research.

We're testing NeuOst, a new drug-free therapy, to find a better solution for your pain. NeuOst involves manual therapy, exercises, and strategies to help you cope better with pain. This therapy was developed with leading experts and patients like you!

In this research, we want to find out how patients like this new treatment and if a larger trial is possible.

WHO CAN GET INVOLVED?

We are looking for people with diabetes and foot or nerve pains.
Contact us now if you think you qualify!

WHAT WOULD I BE DOING?

Participate in a 16-week study at the
Health Sciences University, UCO School of Osteopathy clinic
in Southwark Bridge Road, SE1 0BQ.

If you are assigned to one of the treatment groups, trained osteopaths will administer treatments and look after you during up to 5 visits in our clinic. You will then have 3 phone calls to answer questions about your symptoms and your study experience.
Your input matters, and you'll be compensated for your time and expenses.

GET IN TOUCH!

The OSTEOPATHIC FOUNDATION

Email: David.Schmidt@uco.ac.uk

Call: 07523 629 286

To find out more, visit www.uco.ac.uk/NeuOst or scan this code:

NeuOst
