# Supplementary material for: A multimodal intervention of manual therapy, exercise, and psychological management for painful diabetic neuropathy: intervention development and feasibility trial protocol
Source: Pain Manag. 2025 Jun 11;15(7):387–99. doi: 10.1080/17581869.2025.2515010 (PMC12218422; doi:10.1080/17581869.2025.2515010)
Supplement: Supplemental Material [file IPMT_A_2515010_SM6913.zip › suppl_data/S6 Participant_Information_NeuOst_PIS_V1.1.pdf]

Participant Information Sheet

Version 1.1; 6 September 2024

Study Title:

‘NeuOst’: A Multimodal Manual Therapy-Based Intervention for People with Painful Diabetic Neuropathy: Feasibility of a Randomised Controlled Efficacy Trial

For a video and audio version of this information, please visit:
https://youtu.be/_NKlo4wOqaI ,
Search “Participant Information Sheet: 'NeuOst' Study” on YouTube,
or scan this QR code with your phone:

Invitation

We would like to invite you to be part of this research study. Before you decide, we want to explain why we are doing this research and what it would mean for you. You can talk to others about the study if you want and ask us any questions if something is not clear. We want you to feel comfortable and informed before you make your decision.

Please take time to read the following information and to decide whether you wish to take part.

Thank you and kind regards,

Dr David Hohenschurz-Schmidt (Principal Researcher) & the NeuOst Team

What is the purpose of the study?

We want to find a better way to help people who have a health problem called "painful diabetic neuropathy." This happens when someone with diabetes has a lot of pain in their feet. Many people with diabetes suffer from this pain, and it can make life difficult. There are not many good treatments available for this pain, and some of the medicines that doctors use can have side effects. We want to try a new treatment called "NeuOst" to see if it can make the situation better.

NeuOst is a combination of different drug-free therapies that aim at improving wellbeing. This approach includes hands-on manual therapy, exercises, and learning ways to address feelings and thoughts about living with painful neuropathy.

Before we can try NeuOst with a lot of people, we need to see if it works well and if people can use it easily. So, we are doing a small test first. We taught trained therapists (osteopaths) how to do NeuOst, and now they will treat a few people with painful diabetic neuropathy. We will compare this new treatment to another treatment and the usual care received by participants. The other treatment is a modified version of NeuOst which lacks some of the parts we are interested in, allowing us to compare them.

We also want to see if people like NeuOst and if they find it helpful. This information will help us decide if we should do a bigger study with more people later. We hope that NeuOst could become a good way to help many people with this painful problem.

Why have I been invited?

You are invited to be part of this study because you have painful diabetic neuropathy or have diabetes and pain in your feet. You are not the only one chosen for this study; there will be about 35 other participants.

Do I have to take part?

No. It is up to you to decide whether or not to take part. If you do decide to take part, you will be given this information sheet to keep and be asked to sign a consent form. If you decide to take part, you are still free to stop your participation at any time and without giving a reason. A decision to stop the study or not to take part will *not* affect the standard of care you receive at the Health Sciences University (HSU) - UCO School of Osteopathy or elsewhere.

When and where will this study take place?

If you decide to participate in this study, you will be part of a research trial called NeuOst. From April 2024 on, the trial takes place at the clinic of Health Sciences University (HSU) - UCO School of Osteopathy, located in Southwark in central London (118 Southwark Bridge Road, London SE1 0BQ). The clinic provides a comfortable and accessible environment, with

individual treatment rooms and wheelchair access. It is located 500m from Borough tube station, 850m from London Bridge Station, and local busses stop directly at the clinic.

Treatments during the trial will be delivered by fully qualified and registered osteopaths who have been specially trained to deliver NeuOst. There are up to 5 study visits, followed by two phone calls - all over the course of about 16 weeks. Appointments can be scheduled flexibly from Monday to Saturday.

What exactly will happen to me if I take part?

The study involves the following steps over the course of around 16 weeks:

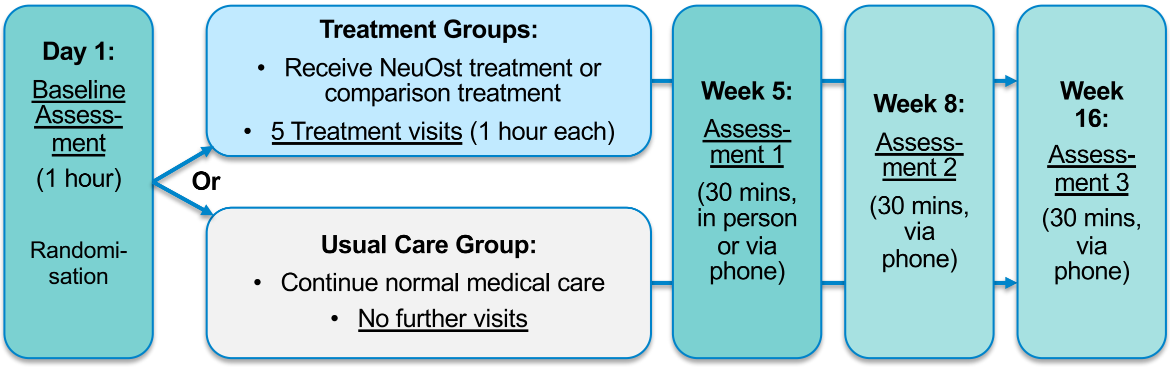

Before the baseline assessment, you would have had (or may already have had) a phone call to determine if you qualify to participate. You will then be sent this Participant Information Sheet and a Consent Form. You can talk to others about the study if you want and please ask us any questions if something is not clear. We want you to feel comfortable and informed before you make your decision.

If you decide to go ahead, you will have to sign the Consent Form and return it to us, either via the post or when you come in for a baseline assessment. After this, the study starts for you.

The '<u>Baseline assessment</u> can be done via the phone or on a videocall, or in person at the clinic. For this assessment, a member of the study team will ask you various health-related questions. The questions will cover topics such as pain, diabetes, medications, physical activity, general health, mental health, sleep quality, and social functioning. This assessment should take about 1 hour.

During the clinical interview or any of your study visits, if any reasons are found that suggest you should not have been included in the study, such as safety concerns with manual therapy or exercises, you will be informed, and you will not continue with the study.

If you are found eligible and choose to participate, you will be randomly assigned to one of three groups ('<u>Randomisation</u>'). You will either receive: The NeuOst intervention; a specially designed control intervention; or no further treatment as part of the study. If you're in the last group, called the 'Usual Care Group,' you will continue with your normal medical care

only. With us, you will have regular phone calls to check in on how you're doing (‘Assessments’), but no treatment visits. This Usual Care Group is important so we can compare our treatments to the normal NHS care.

Patients assigned to the other groups will receive the NeuOst treatment or a similar, comparison treatment during 5 treatment visits (with one treatment every week).

The control or comparison treatment is used because we are still in the process of testing if the new NeuOst treatment is helpful, so we need a comparison. However, the control treatment is the same as the NeuOst intervention apart from a few components, the effects of which we aim to study. Neither you nor clinic staff (other than your direct treatment provider) will know which of the two interventions you receive. This is important so that we can compare the effects of the treatments irrespective of your expectations and beliefs about them.

Your first treatment visit with one of the study osteopaths will take about 90 minutes as they will need to establish more about your health and get to know you. Ideally, this first treatment will take place only a few days after the baseline assessment, and can be scheduled to fit your schedule. Treatments consist of hands-on manual therapy, talking about your experiences, learning important things about diabetes and its complications, and you may also be given exercises to do at home.

After the first treatment, up to 4 more treatment visits will be scheduled ideally one week apart ('Treatment visits’), each lasting about 1 hour. During these visits, you will complete short weekly questionnaires about your health and daily activities, and you will receive the trial intervention based on your random assignment. Please note that some treatment sessions may also be audio- and video-recorded for quality control reasons. You may, however, opt out of such recordings.

After 5 weeks patients in all groups will be asked to complete the same questionnaires as during the baseline testing (‘Assessment 1'). Additionally, after 8 and after 16 weeks, a study team member will call you to go through these questionnaires again (‘Assessment 2 & 3'). Attending these phone calls is crucial for the study to gather important information about how you feel regarding the study treatment. If you wish, you can also participate in an interview and provide feedback for future development of NeuOst.

Throughout the study, appointment scheduling will be flexible, and the clinic's reception team will work with you to find suitable times for your visits. There is no minimum number of required visits, and if needed, appointments can be rescheduled within certain time frames. Ideally, however, you attend all 5 clinical visits if you are allocated to a treatment group. It is also important to take part in the testing phone calls so that we can learn about your progress.

Your participation in this study will help us understand if the NeuOst intervention is feasible and safe, and if it could be a promising treatment for people with painful diabetic neuropathy. Your well-being and comfort are essential to us throughout the trial, and we will make every effort to accommodate your needs during this research.

At the end of the study, you will be informed of the findings, which treatment you received, and you can ask any questions you may have.

What do I have to do to take part?

If you would like to take part in the study or would like further information, please contact the principal researcher by phone on 07523629286 or email via David.Schmidt@uco.ac.uk. If visiting the clinic, ask a member of the study team for the consent form, and sign to enter the study.

You will then be expected to complete the above steps, although you can leave the study at any point without giving reasons.

What are the possible disadvantages and risks of taking part?

To make an informed decision about taking part, it is important that you understand the possible risks involved in this study. Please read this section carefully and ask questions if you would like further information or have any concerns.

Overall, we do not expect any major risks to you personally from this study.

One disadvantage is the time required from you to attend study testing sessions and treatments of which we don't know if they work. As described below, this is often the case in research studies.

You might share personal or sensitive information with the study staff or your treating osteopath, and they will handle it confidentially. Your information and clinical notes will be protected according to the regulatory requirements of the osteopathic profession and UK scientific research standards. You may also be asked to partially undress for the treatment. Where desired, privacy gowns can be provided, and you can ask for a therapist of the same gender. Please let the clinic staff know if you'd prefer that. Generally, bringing loose-fitting clothing, such as trousers that can be rolled up over your knees, is advisable.

Some people can experience soreness after osteopathic treatments, but this is usually short-term and self-limiting. Also, you will be asked to perform exercises in the clinic and at home. These exercises may also cause some muscle soreness and slightly increase your risk of falling. However, your osteopath will practise these exercises with you. To make them safe, all exercises will be adapted to your needs and abilities.

As you are aware, people with diabetes may have low-sugar episodes that can pose a health risk. We don't expect this study to increase this risk, and all study clinicians are trained to recognise and manage these hypoglycaemic episodes.

This study does not affect the care you otherwise receive from your doctors or change the medications you take. We only ask you to not see other osteopaths, chiropractors, massage

therapists, or physiotherapists during the study period. This is necessary for us to be able to tell that any changes you may experience are due to our treatment, not somebody else's.

What are the possible benefits of taking part?

We want to be upfront with you and let you know that participating in this study might not directly benefit you personally. The treatments we are testing may or may not bring immediate benefits to you, and there is a 33% chance that you may not receive a treatment at all. We are conducting this study because we don't have enough information yet to know if the treatment will be helpful.

However, by joining this study, you'll play a role in advancing scientific knowledge. While there's a possibility that the research could lead to a new and effective treatment for people with painful diabetic neuropathy, we can't guarantee that outcome at this stage. However, by participating, you will contribute to the broader understanding of this condition and potentially pave the way for better treatments in the future. We truly appreciate your willingness to be a part of this research journey!

Will I be reimbursed for my expenses and compensated for my time?

You will be reimbursed for your travel expenses to each in-person clinic visit. The travel reimbursement is limited to £11.40, which is the maximum pay-as-you-go travel cost within Zones 1-4 of the TFL network (as of late 2023). Additionally, you will be compensated for your time with payments of £15 per testing session. Together, you can receive a maximum of 4 payments for testing, or £60, regardless of how long each appointment takes.

These payments are there to support you to take part in this study but should not be seen as the only reasons to participate. If needed, you may receive the payments after each testing appointment, or they can be given together at the end of the study.

What if there is a problem?

If you wish to stop participating and withdraw from the study, you are free to do so at any time and without giving a reason. Leaving the study will have no negative effects on your legal rights or your medical care from your GP or specialist providers. Study-related treatments and assessments would stop after withdrawing.

If you have any concerns about the nature or conduct of this research study- or the treatment providers, please contact the Principal Researcher Dr David Hohenschurz-Schmidt or another member of the research team. If you feel David cannot adequately assist you, you can also contact Steven Vogel, who represents the study's host institution (S.Vogel@uco.ac.uk) or Dr David Evans (dwe@backpainclinic.co.uk), the chair of the independent study oversight committee.

Will my participation in the study remain confidential?

Yes. All data collected during the study will be treated as strictly confidential. Apart from your clinical notes, any information gathered for research purposes will be anonymised to protect your identity. Only the research team will have access to the data, including the Principal Researcher and staff involved in data analysis. The study has received approval from Health Sciences University - UCO School of Osteopathy Research Ethics Committee, which ensured that UK ethical and data protection standards are followed. Your personally identifiable information will be securely stored on password-protected servers at HSU-UCO School of Osteopathy. At the study's conclusion, these data will be securely stored at the HSU-UCO School of Osteopathy and later destroyed in accordance with research guidelines. Non-identifiable data will be shared publicly online for other researchers to use freely, for example to check our results or conduct similar research.

What will happen to the results from the study?

The final study results will be shared with the funders of the project and disseminated to the public as part of academic conferences and research articles. There may also be magazine articles, blog and social media posts, videos, or podcasts about what we have found in this study, but your identity and personal information will be always protected. Depending on the results, next steps may include conducting a larger clinical trial, modifying the project, or abandoning it. We will also share any unidentifiable data publicly, to facilitate further research.

You and all other participating patients will receive a summary of the study result - if you tick the corresponding box on the consent form. Participating osteopaths and research team members will also receive a summary of the full study results.

Who is organising the research?

The study is led and coordinated by Dr David Hohenschurz-Schmidt, who is a researcher at HSU-UCO School of Osteopathy and at Imperial College London. The study's host institution (the UCO) is represented by Prof Steven Vogel, who is Professor of Musculoskeletal Health and Care at HSU-UCO School of Osteopathy.

The study was developed and will be analysed by Dr Hohenschurz-Schmidt and a team of researchers, including Sasha Smith (Imperial College London), Prof Annina Schmid (University of Oxford), Dr Esther Williamson (University of Oxford), Dr Whitney Scott (King's College London), Dr Jan Vollert (University of Exeter), Prof Esther Pogatzki-Zahn (Muenster University Hospital), and Prof Andrew SC Rice (Imperial College London).

There is an independent Trial Steering Committee, monitoring the quality and ethical conduct of the study. The Chair of this committee is Dr David W. Evans (University of Birmingham) and the patient representative is Elizabeth Pigott.

Were other people with painful diabetic neuropathy involved in developing this study?

Yes. Trial procedures, information materials, and the NeuOst treatment and the control intervention were developed and pilot-tested with our patient partners: lay people living with painful diabetic neuropathy. One of our patient partners is also part of the trial monitoring committee.

Thank you for taking the time to read this information sheet. Contact details are given below should you have any questions or want further information.

Contact details of the study team

**Principal Researcher**	Dr David Hohenschurz-Schmidt<br>David.Schmidt@uco.ac.uk; Health Sciences University – UCO School of Osteopathy, 275 Borough High Street, London SE1 1JE, UK<br>NeuOst Study Phone: 07523629286
**Sponsor**	Prof Steven Vogel DO<br><br>Steven.vogel@uco.ac.uk; Health Sciences University – UCO School of Osteopathy, 275 Borough High Street, London SE1 1JE, UK
**Funder(s)**	The Osteopathic Foundation / Institute of Osteopathy<br><br>Manjeet Kaur (Grants and Projects Manager); Manjeet@iosteopathy.org; 3 Park Terrace, Manor Road, Luton LU1 3HN, UK
**Trial Steering and Ethical Oversight Committee**	Dr David W. Evans (Chair of Trial Steering Committee)<br><br>Mrs Elizabeth Pigott (Patient Partner)<br><br>D.W.Evans@bham.ac.uk / dwe@backpainclinic.co.uk; Centre of Precision Rehabilitation for Spinal Pain (CPR Spine), University of Birmingham, Edgbaston, Birmingham, B15 2TT, UK<br><br>(Contact to the patient partner can be established through the Chair if required)

Please find the consent form on the following page. Two forms are attached; one should be filled out for the research team, another for you.

You can sign the consent forms in the presence of a NeuOst researcher or in your own time and post them to the below address. If desired, please contact us for a pre-paid envelope.

To:
Dr David Hohenschurz-Schmidt – NeuOst
Health Sciences University – UCO School of Osteopathy
275 Borough High Street
London SE1 1JE, UK
