# Supplementary material for: A multimodal intervention of manual therapy, exercise, and psychological management for painful diabetic neuropathy: intervention development and feasibility trial protocol
Source: Pain Manag. 2025 Jun 11;15(7):387–99. doi: 10.1080/17581869.2025.2515010 (PMC12218422; doi:10.1080/17581869.2025.2515010)
Supplement: Supplemental Material [file IPMT_A_2515010_SM6913.zip › suppl_data/S7 Consent_form_NeuOst_V1.1_6_Sept_2024.docx]

# Consent Form for Participants Able to Give Consent

V1.1, 6 September 2024

Centre name: Health Sciences University - UCO School of Osteopathy, London, UK

Study Protocol number: N/A

**Full Title of Project:** A Multimodal Manual Therapy-Based Intervention for People with Painful Diabetic Neuropathy: Feasibility of a Randomised Controlled Trial (NeuOst)

Name of Principal Investigator: Dr David Hohenschurz-Schmidt

Contact details:

Phone: 07523629286

Email: [David.Schmidt@uco.ac.uk](mailto:David.Schmidt@uco.ac.uk)

Postal: Health Sciences University - UCO School of Osteopathy

**Please initial boxes to indicate your consent to individual items.**

275 Borough High Street

London SE1 1JE, UK

| 1. I confirm that I have read and understand the participant information sheet version 1.1 dated 06 September 2024 for the research project titled ‘NeuOst: A Multimodal Manual Therapy-Based Intervention for People with Painful Diabetic Neuropathy: Feasibility of a Randomised Controlled Efficacy Trial’ and have had the opportunity to ask questions which have been answered to my satisfaction. 2. **(Consent required for participation.)** |  |
| --- | --- |
| 1. I understand that my participation is voluntary, and I am free to withdraw at any time, without giving any reason and without my legal rights nor treatment / healthcare being affected.   **(Required for participation.)** |  |
| 1. I understand that sections of any of my medical notes may be looked at by responsible individuals from Health Sciences University – UCO School of Osteopathy, London, or from regulatory authorities where it is relevant to my taking part in this research.   **(Required for participation.)** |  |
| 1. I understand that data collected from me are a gift donated to Health Sciences University – UCO School of Osteopathy and that I will not personally benefit financially if this research leads to an invention and/or the successful development of a new test, medication treatment, product or service.   **(Required for participation.)** |  |
| 1. I consent to the open sharing of any data collected during this trial that cannot be traced back to me.   **(Required for participation.)** |  |
| 1. I consent to take part in the research project titled ‘NeuOst: A Multimodal Manual Therapy-Based Intervention for People with Painful Diabetic Neuropathy: Feasibility of a Randomised Controlled Efficacy Trial’. **(Required for participation.)** |  |
| 1. I give consent for unidentifiable information collected from me to be used to support other research or in the development of a new test, medical device or treatment by an academic institution or commercial company in the future, including those outside of the United Kingdom (which Health Sciences University – UCO School of Osteopathy has ensured will keep this information secure).   **(Optional item. Only initial the box if you agree.)** |  |
| 1. I give consent to my treatment sessions being audio- and video-recorded for quality assurance purposes.   **(Optional item. Only initial the box if you agree.)** |  |
| 1. I give consent to my treatment sessions being observed by medical or research staff (in addition to the treating osteopath) for quality assurance purposes.   **(Optional item. Only initial the box if you agree.)** |  |
| 1. I give consent to being contacted at the end of the study about the possibility to take part in interviews with the research team about my experiences in the present study. **(Optional item. Only initial the box if you agree.)** |  |
| 1. I give consent to being contacted about the possibility to take part in any other research studies.   **(Optional item. Only initial the box if you agree.)** |  |

Please note that while you can withdraw your consent for data collection and study participation at any time, this will not preclude the collection of information about serious health-related events, which we will be required to collect for several months after study initiation for legal purposes.

I would like to receive a summary of the study results.

________________________ ________________ ________________

Name of participant Signature Date

_________________________ ________________ ________________

Name of person taking consent Signature Date

(if different from Principal Investigator)

*1 copy for participant; 1 copy for Principal Investigator; 1 copy for clinical notes*

*To ensure confidence in the process and minimise risk of loss, all consent forms must be printed, presented, and stored in double sided format.*
